# Insights Into the Detection Selectivity of Redox and Non-redox Based Probes for the Superoxide Anion Using Coumarin and Chromone as the Fluorophores

**DOI:** 10.3389/fchem.2021.753621

**Published:** 2021-11-25

**Authors:** Yuchen Wang, Shumi Jia, Zhenyan Yu, Hui Wen, Huaqing Cui

**Affiliations:** State Key Laboratory of Bioactive Substances and Function of Natural Medicine, Institute of Materia Medica, Peking Union Medical College and Chinese Academy of Medical Sciences, Beijing, China

**Keywords:** coumarin, ROS, superoxide anion, redox-based probes, non–redox-based probes, selectivity

## Abstract

In this study, we evaluated the applicability of various superoxide anion sensors which were designed based on either redox or non-redox mechanisms. Firstly, both redox- and non–redox-based superoxide anion probes were designed and synthesized using either coumarin or chromone as the fluorophores, and the photophysical properties of these probes were measured. Subsequently, the sensing preference of both types of probes toward various reactive oxygen species (ROS) was evaluated. We found that non–redox-based O_2_^•−^ probes exhibited broad sensing ability toward various ROS. By contrast, redox based O_2_^•−^ probes showed a clear reactivity hierarchy which was well correlated to the oxidizing strength of the ROS. Lastly, the detection selectivity of redox-based O_2_^•−^ recognizing probes was also observed when balancing various factors, such as reactant ROS concentrations, temperature, and changing reaction transformation rates. Herein, we concluded the selectivity advantage of redox-based O_2_^•−^ probes.

## Introduction

Reactive oxygen species (ROS) are a group of important oxidizing agents within biological systems, which play a key role in the regulation of homeostasis (Juan et al., 2021; Yang et al., 2019; D’Autréaux and Toledano, 2007). The concentrations of ROS generally remain balanced, and any interruption of this balance results in a cascade of unwanted biological events (Yang et al., 2019; Juan et al., 2021). Therefore, in the clinical setting, it is of utmost importance to accurately detect the concentrations of these ROS, as well as probe the underlying biological mechanism of this dysregulation.

The oxygen of ROS is in a highly oxidizing state, which results in all ROS being highly reactive toward a range of biological substances (Jiao et al., 2018). Thus, ROS are usually found in low concentrations in tissues under regular conditions, and therefore traditionally, it has been difficult to accurately quantify the concentration of ROS. The mitochondria and NADPH oxidase produce the major ROS, superoxide anion, and H_2_O_2_ in the cells (Dröge, 2002; Woolley et al., 2013). Usually, under normal conditions, the concentration of superoxide anion and H_2_O_2_ is estimated to be about 10^−10^ and 5 × 10^−9^ M, respectively (Dröge, 2002; Turrens, 2003; Woolley et al., 2013). However, the concentration of these at the cellular level can change in a wide range under stimulated conditions. Moreover, the oxidizing state of ROS ranges from 2 to 0, with degradation from high-oxidizing ROS forming additional low-oxidizing ROS (Jiao et al., 2018). Therefore, several ROS might coexist within a single system, and it will be important to distinguish each ROS during detection (Jiao et al., 2018). Among the ROS, the oxygen of the superoxide anion is in the highest oxidation state, and the superoxide anion is the precursor to several other ROS (Cadenas and Davies, 2000; Jiao et al., 2018). Thus, in regard to superoxide anion detection, selectivity would be a key parameter to be considered.

**FIGURE 1:**
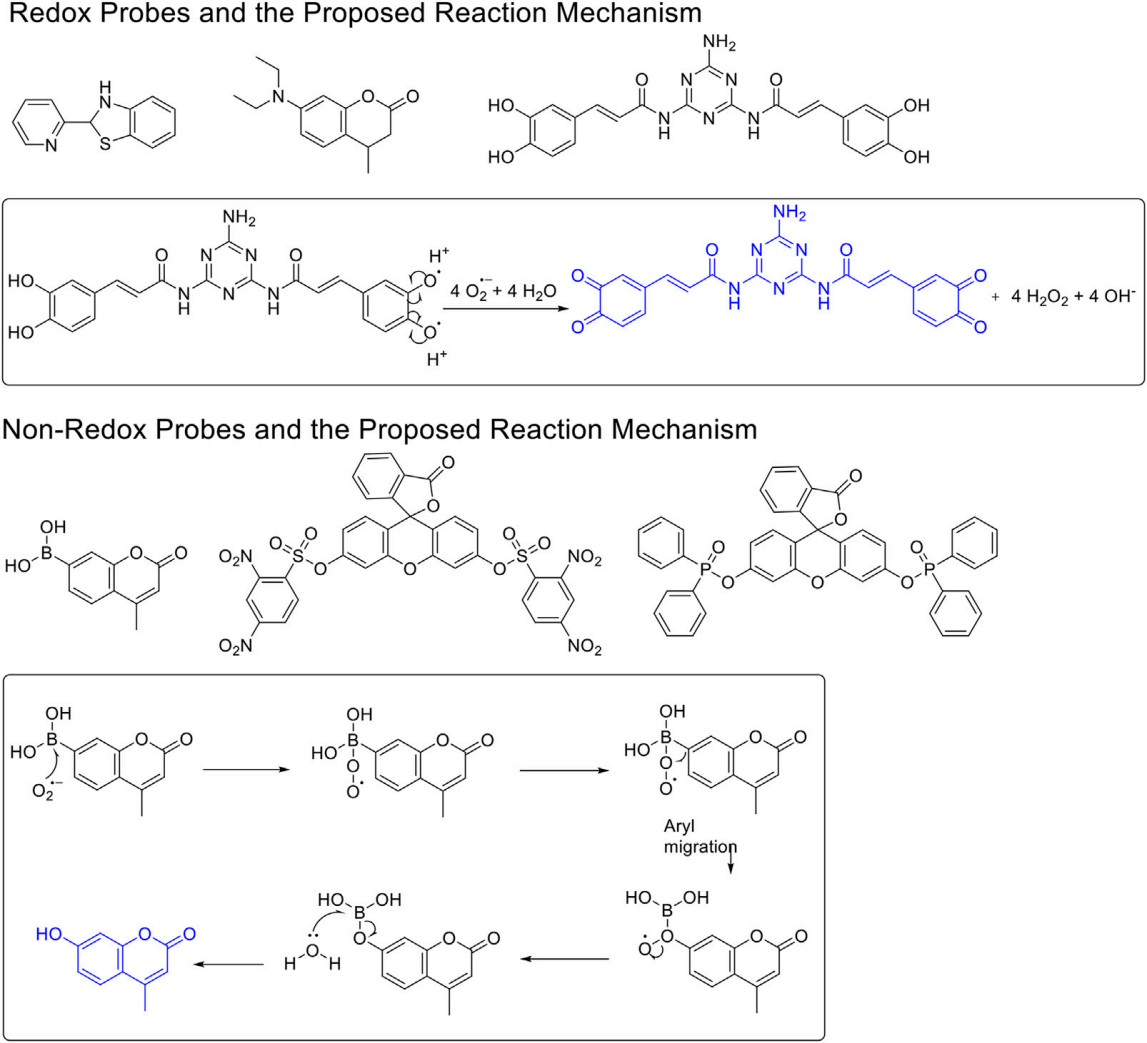
The redox- and non–redox-based superoxide anion probes. The reaction mechanisms of the selected probes were also proposed to help to understand this study.

To this end, various methods have been developed for ROS detection, including the fluorescent dye method, nanoprobe technology, electrochemical biosensors, electron spin resonance method, genetic encoded ROS reporter, and others (Woolley et al., 2013; Mamone et al., 2016) Among them, fluorescent techniques have been widely used in sensing and detecting these biologically important species under certain biological conditions(Fuloria et al., 2021; Duanghathaipornsuk et al., 2021; Wu et al., 2019; Cheng et al., 2019). To date, a range of fluorescent sensors have been developed for various ROS (Chen et al., 2016; Yang et al., 2020). For O_2_^•−^ sensors, based on design principles, these can be classified into two categories: redox and non-redox mechanisms–based O_2_^•−^ fluorescent probes (Figure 1) (Jiao et al., 2018; Xiao et al., 2020). The redox-based fluorescent probes have been designed based on the oxidizing ability of O_2_^•−^ (Tang et al., 2004; Zhang et al., 2013; Wang et al., 2020), and the non–redox-based fluorescent probes were designed on the nucleophilicity or other inherent reactivity of O_2_^•−^ (Maeda et al., 2005; Xu et al., 2007; Zielonka et al., 2010). Although these descriptors exist, there has been no systematic study to understand their key differences in relation to their applicability, particularly, their sensing selectivity.

## Materials and Methods

### Chemical Synthesis General

^1^H-NMR and ^13^C-NMR spectra were recorded with a Varian Mercury 400 or 500 spectrometer using tetramethylsilane as the internal standard in methanol-*d*_*4*_, DMSO-*d*_*6*_, or chloroform-*d*. High-resolution mass spectrometry (HRMS) data were measured on a Thermo Exactive Orbitrap Plus spectrometer. Liquid chromatography–mass spectrometry (LC-MS) was conducted on an Agilent 1100 series HPLC and an Agilent LC/MSD TOF. All of the solvents and chemicals were purchased from commercial sources: Sigma-Aldrich Chemical Co., Beijing Ou-he Reagents Co., Beijing Shiji-Aoke Biotechnology Co., and Shanghai Jingke Chemistry Technology Co. with a purity of more than 95% (LC-MS). All chemicals and solvents used were of reagent grade without further purification or drying before use. All the reactions were monitored by thin-layer chromatography (TLC) under a UV lamp at 254 nm. Column chromatography separations were performed using silica gel (200–300 mesh).

#### General Procedure for Preparation of Compounds R1–R3

To a solution of compound **1** or **2** or **3** (1.40 mmol) in absolute alcohol (10 ml), palladium on carbon (10% Pd/C, 10 wt% of the compound **1** or **2** or **3**) was added and the suspension was hydrogenated (1 atm, balloon) at RT for 22 h. TLC indicated the completion. The suspension was filtered through a pad of Celite and the filtered solid was rinsed with ethyl acetate (3 × 10 ml). The combined filtrate and rinses were concentrated. The products **R1–R3** were purified by silica gel column chromatography.

7-Hydroxy-4-methylchroman-2-one (**R1**). White solid, yield 23.5%. ^1^H-NMR (400 MHz, methanol-*d*_*4*_): δ 7.09 (d, *J* = 8.2 Hz, 1H), 6.58 (dd, *J* = 8.2, 2.2 Hz, 1H), 6.45 (d, *J* = 2.1 Hz, 1H), 3.10 (dd, *J* = 12.2, 6.0 Hz, 1H), 2.83 (dd, *J* = 15.6, 5.0 Hz, 1H), 2.53 (dd, *J* = 15.6, 6.7 Hz, 1H), 1.26 (d, *J* = 6.8 Hz, 3H). ^13^C-NMR (100 MHz, methanol-*d*_*4*_): δ 170.64, 158.72, 153.22, 128.37, 120.11, 112.75, 104.56, 37.94, 30.03, 20.53. HRMS (ESI): *m/z* calculated for C10H11O3 (M + H)^+^, 179.07027; found, 179.07010.

7-Amino-4-methylchroman-2-one (**R2**). White solid, yield 15.6%. ^1^H-NMR (400 MHz, methanol-*d*_*4*_): δ 6.99 (d, *J* = 8.2 Hz, 1H), 6.49 (dd, *J* = 8.1, 2.1 Hz, 1H), 6.38 (d, *J* = 2.1 Hz, 1H), 3.12–2.97 (m, 1H), 2.81 (dd, *J* = 15.7, 5.5 Hz, 1H), 2.51 (dd, *J* = 15.7, 7.0 Hz, 1H), 1.24 (d, *J* = 6.9 Hz, 3H). ^13^C-NMR (100 MHz, methanol-*d*_*4*_): δ 171.05, 153.25, 149.46, 128.14, 118.15, 112.78, 104.03, 38.19, 30.00, 20.61. HRMS (ESI): *m/z* calculated for C10H12NO2 (M + H)^+^, 178.08626; found, 178.08673.

7-(Diethylamino)-4-methylchroman-2-one (**R3**). Colorless oily liquid, yield 30.0%. ^1^H-NMR (400 MHz, DMSO-*d*_*6*_): δ 7.01 (d, *J* = 8.6 Hz, 1H), 6.39 (dd, *J* = 8.5, 2.6 Hz, 1H), 6.24 (d, *J* = 2.6 Hz, 1H), 3.26 (q, *J* = 7.0 Hz, 4H), 3.05–2.94 (m, 1H), 2.77 (dd, *J* = 15.7, 5.5 Hz, 1H), 2.45 (dd, *J* = 15.7, 7.5 Hz, 1H), 1.15–1.08 (m, 3H), 1.04–0.96 (m, 6H). ^13^C-NMR (100 MHz, DMSO-*d*_*6*_): δ 169.04, 152.48, 147.95, 127.62, 114.46, 108.15, 99.46, 44.21 (2C), 37.36, 28.20, 20.55, 12.80 (2C). HRMS (ESI): *m/z* calculated for C14H20NO2 (M + H]^+^, 234.14886; found, 234.14853.

#### General Procedure for the Preparation of Compounds **Ra–Rc**

The compound 4H-chromen-4-one derivative **4** or **5** or **6** (0.41 mmol) was added to dry THF (15 ml), and then the mixture was stirred and cooled to −20°C. A solution of LiAlH_4_ (0.45 ml, 1.0 M solution in THF) diluted with 5 ml dry THF was added dropwise to the above with stirring at −20°C. The mixture was stirred for 2 h at −20°C. The reaction was analyzed by TLC for completion. Then the reaction was quenched with 2 M NH_4_Cl aqueous solution (20 ml), and the solvent was removed in vacuo. The mixture was extracted with ethyl acetate (3 × 20 ml), and the combined organic layers were washed with saturated NaCl aqueous solution (2 × 20 ml). The organic layer was then dried (Na_2_SO_4_), filtered, and the solvent was removed in vacuo. The products **Ra–Rc** were purified by silica gel column chromatography.

7-Hydroxy-3-methylchroman-4-one (**Ra**). White solid, yield 46.2%. ^1^H-NMR (400 MHz, methanol-*d*_*4*_): δ 7.69 (d, *J* = 8.7 Hz, 1H), 6.47 (dd, *J* = 8.6, 2.0 Hz, 1H), 6.29 (d, *J* = 1.9 Hz, 1H), 4.47 (dd, *J* = 11.2, 5.0 Hz, 1H), 4.10 (t, *J* = 10.7 Hz, 1H), 2.84–2.65 (m, 1H), 1.15 (d, *J* = 7.0 Hz, 3H). ^13^C-NMR (100 MHz, methanol-*d*_*4*_): δ 196.05, 166.37, 165.58, 130.14, 114.52, 111.64, 103.46, 73.48, 41.50, 11.24. HRMS (ESI): *m/z* calculated for C10H11O3 (M + H)^+^, 179.07027; found, 179.07027.

7-Amino-3-methylchroman-4-one (**Rb**). Yellow solid, yield 29.3%. ^1^H-NMR (400 MHz, methanol-*d*_*4*_): δ 7.56 (d, *J* = 8.7 Hz, 1H), 6.29 (dd, *J* = 8.7, 2.1 Hz, 1H), 6.06 (d, *J* = 2.1 Hz, 1H), 4.41 (dd, *J* = 11.1, 4.8 Hz, 1H), 4.05 (dd, *J* = 11.1, 9.6 Hz, 1H), 2.67 (qd, *J* = 9.6, 7.1, 4.9 Hz, 1H), 1.15 (d, *J* = 7.1 Hz, 3H). ^13^C-NMR (100 MHz, methanol-*d*_*4*_): δ 195.64, 165.75, 158.12, 130.01, 111.47, 110.39, 99.84, 73.28, 41.32, 11.71. HRMS (ESI): *m/z* calculated for C10H12NO2 (M + H)^+^, 178.08626; found, 178.08649.

7-(Azetidin-1-yl)-3-propylchroman-4-one (**Rc**). White solid, yield 60.3%. ^1^H-NMR (400 MHz, DMSO-*d*_*6*_): δ 7.55 (d, *J* = 8.6 Hz, 1H), 6.07 (dd, *J* = 8.7, 2.1 Hz, 1H), 5.76 (d, *J* = 2.1 Hz, 1H), 4.43 (dd, *J* = 11.3, 4.4 Hz, 1H), 4.17 (dd, *J* = 11.3, 8.1 Hz, 1H), 3.92 (t, *J* = 7.4 Hz, 4H), 2.46 (h, *J* = 3.2 Hz, 1H), 2.33 (p, *J* = 7.3 Hz, 2H), 1.72–1.57 (m, 1H), 1.44–1.37 (m, 1H), 1.33 (ddd, *J* = 13.1, 9.2, 5.6 Hz, 2H), 0.88 (t, *J* = 7.1 Hz, 3H). ^13^C-NMR (100 MHz, DMSO-*d*_*6*_): δ 191.99, 163.07, 156.62, 128.73, 110.65, 105.66, 95.99, 70.51, 51.61, 44.84, 29.02, 20.13, 16.28, 14.49. HRMS (ESI): *m/z* calculated for C15H20O2N (M + H]^+^, 246.14886; found, 246.14819.

#### General Procedure for Preparation of Compounds **N1**, **N2**

To a solution of compound **10** or **11** (0.75 mmol) in 1,4-dioxane (3 ml), Et_3_N (304 mg, 3.00 mmol) and PdCl_2_ (dppf) (23 mg, 0.03 mmol) were added. Then 5,5,5′,5′-tetramethyl-2,2′-bi (1,3,2-dioxaborinane) (509 mg, 2.25 mmol) was added dropwise to the above with stirring. The mixture was stirred and heated to 120°C and refluxed. The reaction was analyzed by TLC for completion. The mixture was cooled to room temperature and 3 ml saturated NH_4_Cl aqueous solution added. The mixture was extracted with ethyl acetate (3 × 10 ml), and the combined organic layers were washed with saturated NaCl aqueous solution (2 × 10 ml). The organic layer was then dried (Na_2_SO_4_), filtered, and the solvent was removed in vacuo. The products **N1**, **N2** were purified by silica gel column chromatography.

7-(5,5-Dimethyl-1,3,2-dioxaborinan-2-yl)-4H-chromen-4-one (**N1**). White solid, yield 75.6%. ^1^H-NMR (400 MHz, chloroform-*d*): δ 8.17 (d, *J* = 7.9 Hz, 1H), 7.89–7.85 (m, 2H), 7.80 (d, *J* = 7.8 Hz, 1H), 6.35 (d, *J* = 5.6 Hz, 1H), 3.81 (s, 4H), 1.04 (s, 6H). ^13^C-NMR (100 MHz, chloroform-*d*): δ 177.98, 156.10, 155.50, 129.99, 126.19, 124.58, 123.63, 116.59, 112.99, 72.46 (2C), 31.92, 21.85 (2C). HRMS (ESI): *m/z* calculated for C14H16O4B (M + H)^+^, 259.11362; found, 259.11300.

7-(5,5-Dimethyl-1,3,2-dioxaborinan-2-yl)-3-propyl-4H-chromen-4-one (**N2**). White solid, yield 82.2%. ^1^H-NMR (400 MHz, chloroform-*d*): δ 8.18 (d, *J* = 7.9 Hz, 1H), 7.85 (s, 1H), 7.76 (t, *J* = 3.9 Hz, 2H), 3.80 (s, 4H), 2.49–2.41 (m, 2H), 1.62 (dd, *J* = 14.9, 7.4 Hz, 2H), 1.04 (s, 6H), 0.97 (t, *J* = 7.3 Hz, 3H). ^13^C-NMR (100 MHz, chloroform-*d*): δ 178.14, 156.03, 152.22, 152.04, 129.46, 125.20, 124.65, 124.47, 123.54, 72.44 (2C), 31.91, 27.87, 21.86 (2C), 21.55, 13.83. HRMS (ESI): *m/z* calculated for C17H22O4B (M + H)^+^, 301.16057; found, 301.16030.

#### General Procedure for Preparation of Compounds **N3**, **N4**

To a solution of compound **1** or **9** (0.57 mmol) in dry DCM (10 ml), DIPEA (220 mg, 1.70 mmol) and 2,4-dinitrobenzenesulfonyl chloride (151 mg, 0.57 mmol) were added. The mixture was stirred at room temperature. The reaction was analyzed by TLC for completion, and the products **N3** and **N4** were purified by silica gel column chromatography.

4-Methyl-2-oxo-2H-chromen-7-yl 2,4-dinitrobenzenesulfonate (**N3**). White solid, yield 45.6%. ^1^H-NMR (400 MHz, DMSO-*d*_*6*_): δ 9.12 (d, *J* = 2.2 Hz, 1H), 8.61 (dd, *J* = 8.7, 2.3 Hz, 1H), 8.32 (d, *J* = 8.7 Hz, 1H), 7.85 (d, *J* = 8.8 Hz, 1H), 7.34 (d, *J* = 2.4 Hz, 1H), 7.23 (dd, *J* = 8.7, 2.4 Hz, 1H), 6.46 (s, 1H), 2.42 (s, 3H). ^13^C-NMR (100 MHz, DMSO-*d*_*6*_): δ 159.60, 154.00, 153.00, 152.10, 150.44, 148.61, 134.13, 131.04, 128.15, 127.97, 121.75, 120.05, 118.61, 115.43, 110.93, 18.64. HRMS (ESI): *m/z* calculated for C16H11N2O9S (M + H)^+^, 407.01798; found, 407.01721.

6-Methoxy-4-oxo-3-propyl-4H-chromen-7-yl 2,4-dinitrobenzenesulfonate (**N4**). White solid, yield 55.2%. ^1^H-NMR (400 MHz, chloroform-*d*): δ 8.90 (d, *J* = 2.7 Hz, 1H), 8.32 (dd, *J* = 9.2, 2.7 Hz, 1H), 7.77 (s, 2H), 7.31 (s, 1H), 6.94 (d, *J* = 9.2 Hz, 1H), 3.85 (s, 3H), 2.52–2.39 (m, 2H), 1.70–1.59 (m, 2H), 0.99 (t, *J* = 7.4 Hz, 3H). ^13^C-NMR (100 MHz, chloroform-*d*) δ 176.65, 155.10, 152.23, 151.09, 148.59, 146.01, 141.92, 139.31, 128.76, 124.31, 122.73, 122.21, 117.95, 111.88, 107.48, 56.55, 27.77, 21.50, 13.80. HRMS (ESI): *m/z* calculated for C19H17N2O10S (M + H)^+^, 465.05984; found, 465.05988.

### Measurement of Photophysical Properties of the Probes

The photophysical properties of all compounds were measured. The measurement of the photophysical properties of various compounds was carried out as we described before (Miao et al., 2015; Wen et al., 2018; Yan et al., 2018). All compounds were dissolved in 0.1 M Tris-HCl buffer, pH 8.0 at the concentration of 10 μM. SHIMADU UV-2700, UV-visible spectrophotometer was used to measure UV-visible spectra. HITACHI F-7000 fluorescence spectrophotometer was used to measure excitation and emission spectra. For fluorescence quantum yield calculation, compounds were dissolved in 0.1 M Tris-HCl buffer (pH 8.0) at the concentration of 0.5 μg/ml or less using quinine sulfate (0.5 μg/L in 0.1 M H_2_SO_4_, Ф = 0.54) as a reference (Williams et al., 1983).

### Detection of the pH Stability of the Probes

We also investigated the stability of these probes under various pHs. 0.2 M phosphate buffers with desired pHs (pH 3, pH 7, and pH13) were prepared. A 10 μM solution of each probe at different pHs (pH 3, pH 7, and pH13) was prepared, and their fluorescence was scanned (*E*_x_, *E*_m_) to see the change.

### Determination of the Reactivity Between Fluorescent Probes and Various ROS

Various ROS were also prepared as literature in 0.1 M phosphate buffer, 0.15 M NaCl, pH 7.4, or anhydrous DMSO (Wang et al., 2020; Chen et al., 2021). Each probe was dissolved in these ROS solutions at the final concentration of 10 μM. After being incubated at 37°C for 5 min, the mixture was scanned for the preferred *E*_x_ of the desired fluorophore to check if the desired fluorophore was formed. In order to detect the fluorescence change, the *E*_x_ was set as 340 nm, and the *E*_m_ was measured between 380 and 600 nm. In addition, the final products of the reactions were also analyzed by LC-MS for confirmation (ESI S2).

### Fluorescence Response of Various Probes Toward XO/HPX System

The enzymatic assay was performed in 0.1 M HEPES buffer, pH 7.4. Please refer to our previous publications (Wang et al., 2020). Initially, we began this study at a relatively low concentration of 0.25 U/ml XO enzyme and observed a real-time fluorescence change for non–redox-based probes **N1**, **N2**, **N3**, and **N4**. However, under these conditions, we did not observe a fluorescence change for redox-based probes **R1**, **R2**, and **R3**. We further increased the concentration of the XO enzymes (0.6 U/ml) to produce more O_2_^•−^ in the system and observed a relatively low fluorescence increase for probes **R2** and **R3**, but no fluorescence change was observed for probe **R1**.

## Results and Discussion

### Synthesis of Various 3,4-Dihydrocoumarin and Chromanone-Derived Probes

#### Series 1

**SCHEME 1:**
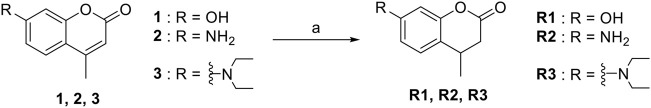
The synthetic route of compounds **R1**, **R2**, **R3**. Reagent and conditions: 10% Pd/C, H_2_, EtOH, RT, Yield: 23.5% **(R1)**, 15.6% **(R2)**, 30% **(R3)**.

The synthesis of probes **R1–R3** is depicted in Scheme 1. Compounds **1**, **2**, and **3** are coumarin derivatives and were prepared as described previously (Wang et al., 2020; Chen et al., 2021). **1**, **2**, and **3** were reduced to probes **R1**–**R3** via hydrogenation, employing 10% Pd/C.

#### Series 2

**SCHEME 2:**
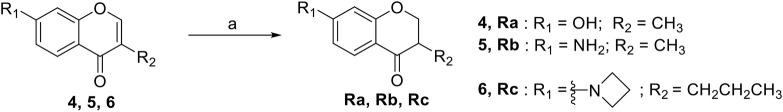
The synthetic routes of compounds **Ra**, **Rb**, **Rc**. Reagent and conditions: LiAlH_4_, dry THF, −20°C. Yield: 46.2% **(Ra)**, 29.3% **(Rb)**, 60.3% **(Rc)**.

The synthetic routes of compounds **Ra**, **Rb**, and **Rc** are outlined in Scheme 2. Three chromones **4**, **5**, and **6** were prepared as previously described (Chen et al., 2021). For the reduction, we used LiAlH_4_ to reduce the chromones to yield the desired chromanones under a low temperature.

#### Series 3

**SCHEME 3:**
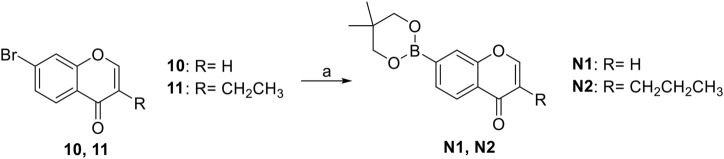
The synthetic route of compounds **N1**, **N2**. Reagent and conditions: (a) Et_3_N, (dppf)PdCl_2_, 1,4-dioxane, 2-(2,2-dimethyl-1,3,5-dioxaborinan-5-yl)-5,5-dimethyl-1,3,2-dioxaborinane, 120°C, reflux, Yields: 75.6% **(N1)** and 82.2% **(N2)**.

The synthetic strategy towards **N1** and **N2** is shown in Scheme 3. Compounds **10** and **11** are commercially available. The compounds **N1** and **N2** were obtained following a previously reported Miyaura borylation protocol (Jana et al., 2014).

#### Series 4

**SCHEME 4:**
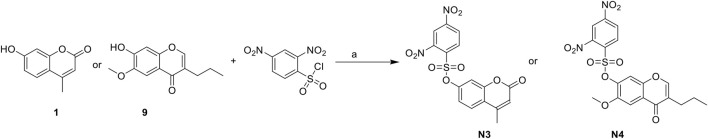
The synthetic routes of N3 and N4. Reagent and conditions: (a) DIPEA, dry DCM, RT, Yield: 45.6% (N3) and 55.2% (N4).

The synthesis of **N3** and **N4** is shown in Scheme 4 and was achieved via a simple one-step procedure from the corresponding phenol and sulfonyl chloride. Compound **1** (100 mg, 0.568 mmol) or compound **9** (100 mg, 0.427 mmol) reacted with 2,4-dinitrobenzene-1-sulfonyl chloride (151 mg, 0.568 mmol) in anhydrous DCM (10 ml) and DIPEA (1.704 mmol, 3 eq). The mixture was stirred at room temperature. The reaction was analyzed by TLC for completion. The yields of **N3** and **N4** were 45.6% or 55.2%.

**FIGURE 2:**
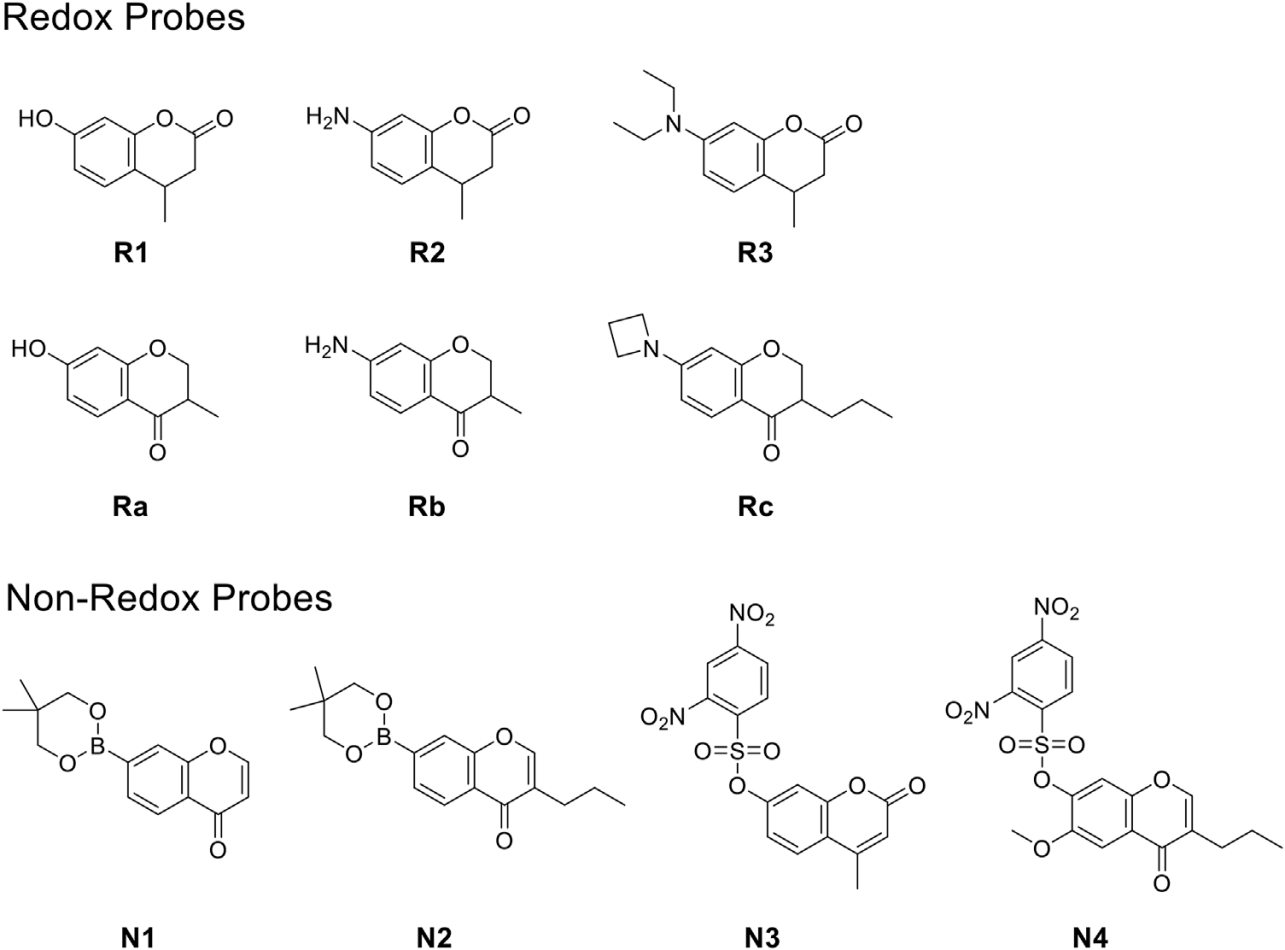
The redox- and non–redox-based superoxide anion probes designed and synthesized in this study. Coumarin and chromone were selected as the core fluorophores.

We, therefore, set out to conduct this study. First, we worked to synthesize ten O_2_^•-^ sensors of both redox- and non–redox-based probes (Figure 2 and Table 1), and 7-donor coumarin and 7-donor chromone were chosen as the fluorophoric portion of the molecule. Coumarin is a known fluorophore and has been frequently used in various studies to a high level of success (Cao et al., 2019). Additionally, chromone derivatives have also been found to exhibit a range of interesting fluorescent properties (Miao et al., 2015; Chen et al., 2021). Series 1 (**R1**, **R2**, **R3**) and series 2 (**Ra**, **Rb**, **Rc**) were designed as redox mechanism–based probes. For these two series, the broken aromatization of either coumarin or chromone quenched the fluorescence. For the detection, the oxidation of them by certain ROS was expected to recover the aromatization of the coumarin or chromone, and this would in accordance turn on the fluorescence (Doura et al., 2012; Wang et al., 2020). Series 3 (**N1**, **N2**) and series 4 (**N3**, **N4**) are the probes that were designed with a non-redox mechanism, and the 7-donor groups were modified with the boronate group (series 3) or sulfonyl ester group (series 4). The modification of the 7-donor group of the coumarin and chromone broke the electron transfer between donor and *p*-conjugated-acceptor, and this dramatically quenched the fluorescence of probes. For the detection, certain ROS will react with the probes to either replace or remove the modified group, and finally turn on the fluorescence (Maeda et al., 2005; Castro-Godoy et al., 2019).

**TABLE 1:** The photophysical properties of various O_2_^•-^ probes and their fluorophores.

Probe^a^	ε_max_ ^b^	Ф^c^	Fluorophore^a^	λ_ex_ ^d^	λ_em_ ^d^	ε_max_ ^b^	Ф^c^	Ratio
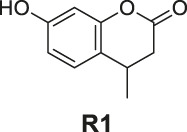	804	0.09	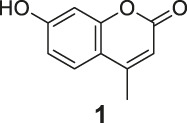	330	450	15,120	0.92	192
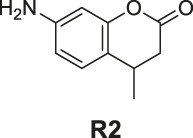	2,356	0.02	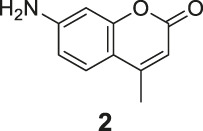	346	446	13,627	0.93	269
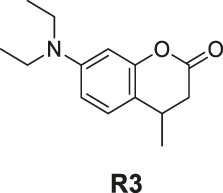	8,787	0.01	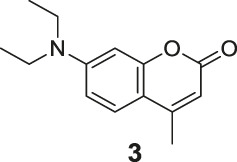	390	476	23,130	0.07	18
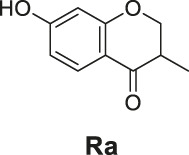	24,310	0.07	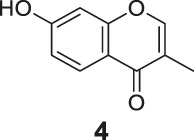	330	476	14,100	0.21	2
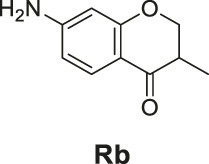	35,500	0.01	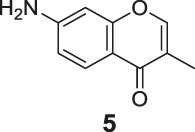	330	450	11,900	0.57	19
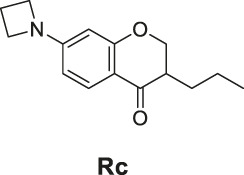	23,765	0.01	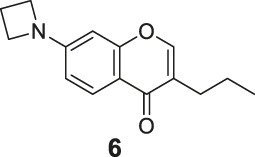	348	482	10,800	0.29	13
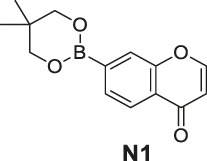	15,361	0.01	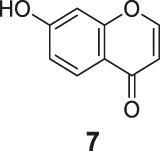	340	480	13,527	0.13	11
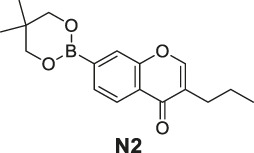	13,904	0.02	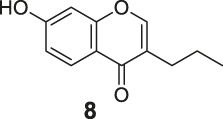	340	468	11,600	0.23	10
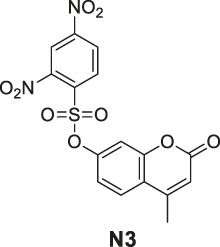	11,765	0.07	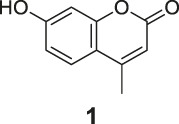	330	450	15,120	0.92	17
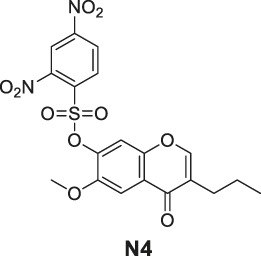	6,727	0.11	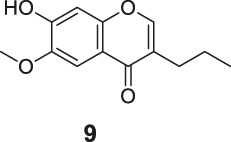	348	450	17,900	0.48	12

a The measurements were taken in 0.1 M Tris-HCl, pH 8.0.

b Unit: M^−1^•cm^−1^.

c Determined with quinine sulfate (Ф = 0.54, 0.1 M H_2_SO_4_); Williams et al. (1983).

d Unit: nm.

### Measurement of Photophysical Properties and pH Stability of the Probes

**FIGURE 3:**
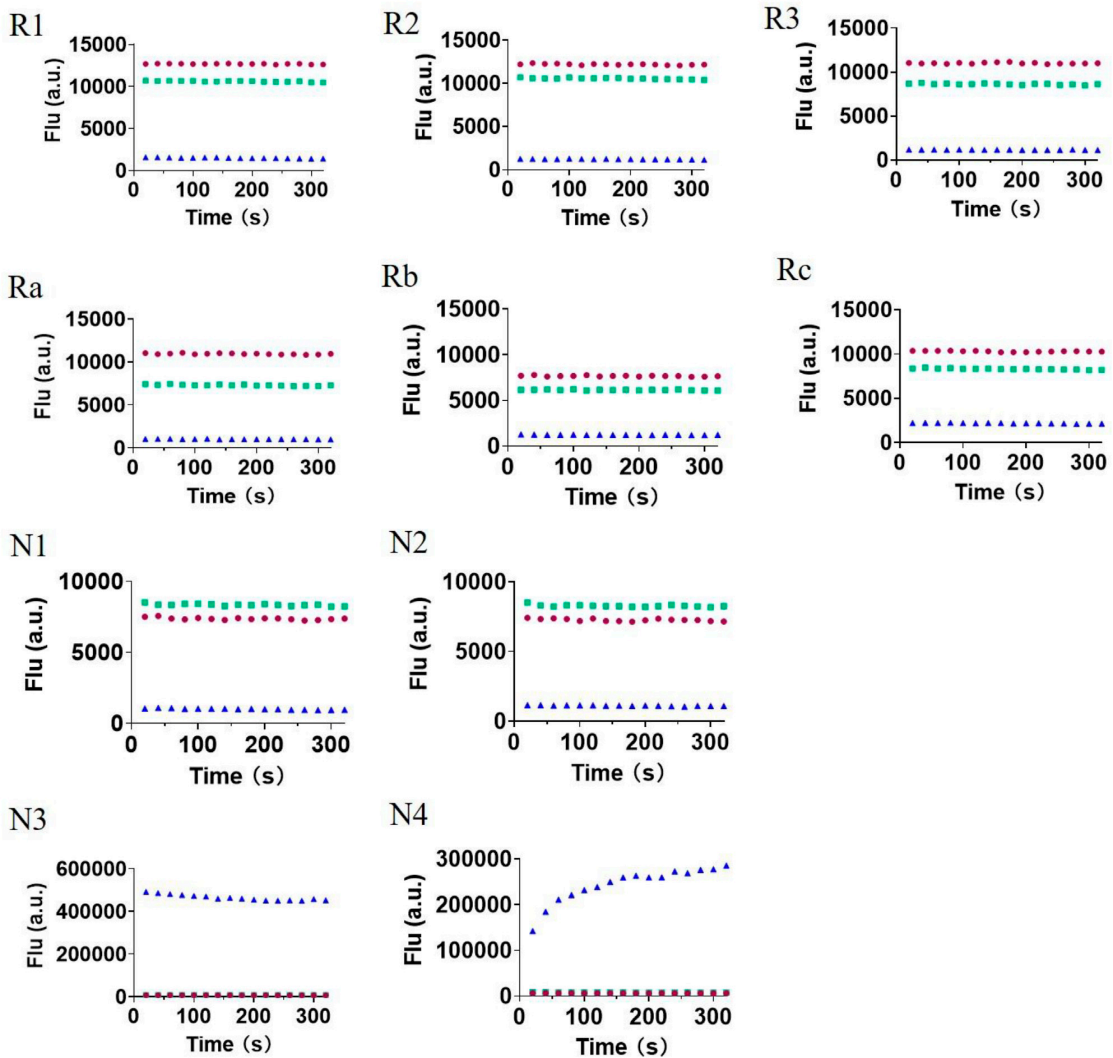
The fluorescence intensity of all 10 probes (**R1–N4**) in various phosphate buffers with different pHs (pH3, red; pH7, green; and pH13, blue). Once the compounds were dissolved, fluorescence intensity was immediately measured for several minutes.

Next, we measured the photophysical properties of all probes (Table 1). As expected, the majority of the fluorophores (**1–9**) exhibited moderate to high quantum yields, while the designed probes (**R1–N4**) had relatively low quantum yields (0.01–0.11). Moreover, given that the fluorescence intensity of a compound corresponds to the quantum yield and the molar extinction coefficient, we calculated the turn-on ratio for each matched pair of probe and fluorophore. As expected, the majority of the synthesized probes have a useful fluorescence turn-on ratio ranging from 10 to several hundred, and are therefore perfectly suited to being used as fluorescence turn-on probes. We further investigated the stability of these probes under various pHs (Figure 3). A 10 μM solution of each probe at different pHs (pH 3, pH 7 and pH13) was prepared and their fluorescence was measured. We can see that redox based O_2_^•−^ probes (series 1, 2) have stable fluorescence intensity in various buffers at differing pH. For the non-redox based O_2_^•−^ probes, we observed a stable but low fluorescence intensity of series 3 in various buffers (pH 3, pH 7, and pH13). However, we found that probes of series 4 (**N3** and **N4**) exhibited strong fluorescence intensity in a basic buffer (pH13), which was 30–40 times stronger than that of other buffers (pH3 and pH7). This suggests that probes **N3** and **N4** degraded under basic conditions to turn on the fluorescence. Since **N3** and **N4** were designed to react with ROS to remove the sulfonyl ester group via nucleophilic substitution, the OH- group in a basic buffer can also react with them to eliminate the modification on 7-hydroxyl (Tampieri et al., 2019). In summary, redox based O_2_^•−^ probes are rather stable at differing pHs. However, in basic conditions, some non-redox based O_2_^•−^ probes might turn the fluorescence on by OH- group *via* nucleophilic substitution.

### Determination of the Reactivity Between Fluorescent Probes and Various ROS

Now that we have a primary understanding of the probes, we would like to explore the reactivity between the probes and various ROS [e.g., tert-butyl hydroperoxide(TBHP), H_2_O_2_, •OH, ^1^O_2_, ClO^−^, O_2_^•−^] (Table 2, ESI). Probes were incubated with various oxidizing agents (TBHP, H_2_O_2_, •OH, ^1^O_2_, ClO^−^) in 0.1 M phosphate buffer with 0.15 M NaCl (pH 7.4) at 37°C for 5 min (Doura et al., 2012; Xing et al., 2016; Zhan et al., 2017). Because O_2_^•−^ cannot exist in an aqueous buffer, the reaction between the probes and O_2_^•−^ was carried out in anhydrous DMSO at 37°C for 5 min (Wang et al., 2020). The concentrations of the probes were set as 10 μM, but the amounts of various ROS were excessive to promote the reaction (ESI). It was agreed that if the desired fluorophore was detected, regardless of the reaction transformation rate, the reactivity between the probe and ROS would be deemed successful in the study. The results (Table 2) showed that under our conditions: (1) **R2** was the most reactive probe in series 1, which can react with ^1^O_2_, ClO^−^, and O_2_^•−^. The other two probes, **R1** and **R3**, can only react with ClO^−^ and O_2_^•−^. (2) Interestingly, chromone-derived probes (series 2) showed much lower reactivity when compared to series 1, and we unfortunately only saw the reactivity between **Rb** and O_2_^•−^. (3) However, we observed that the probes from series 3 and 4 were much more reactive toward various ROS than series 1 and 2. The boronate probes (**N1** and **N2**) were successful in sensing TBHP, H_2_O_2_, ^1^O_2_, •OH, ClO^−^, and O_2_^•−^. The sulfonyl ester (**N3** and **N4**) successfully reacted with TBHP, H_2_O_2_, and O_2_^•−^, but not with ^1^O_2_, •OH, and ClO^−^.

**TABLE 2:** The reactivity between the probes and various ROS.

Compounds	TBHP	H_2_O_2_	•OH	^1^O_2_	ClO^−^	O_2_^•−^
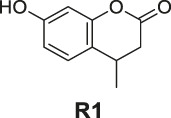	−	−	−	−	+	+
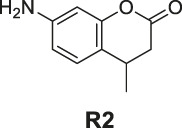	−	−	−	+	+	+
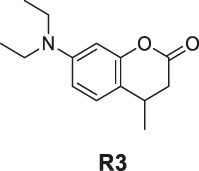	−	−	−	−	+	+
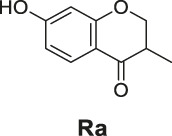	−	−	−	−	−	−
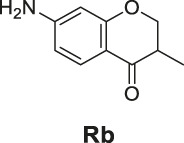	−	−	−	−	−	+
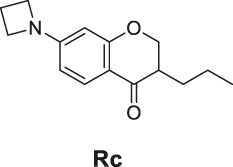	−	−	−	−	−	−
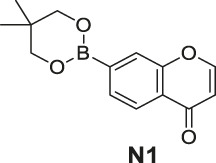	+	+	+	+	+	+
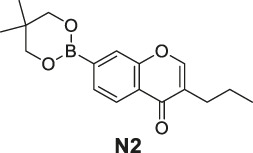	+	+	+	+	+	+
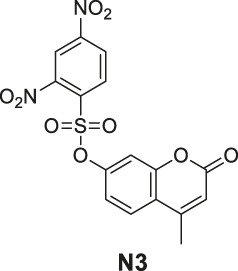	+	+	−	−	−	+
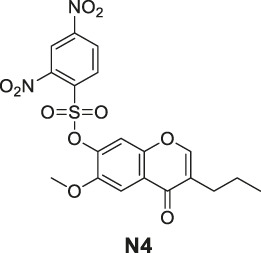	+	+	−	−	−	+

In this section, in order to rank the reactivity hierarchy toward various ROS among probes, we used excessive ROS to react with each probe. Generally speaking, redox-based O_2_^•−^ probes exhibited a strong reactivity hierarchy which was well correlated to the oxidizing state of the ROS. The reactivity order of redox-based O_2_^•−^ probes is **R2** > **R3** > **R1** > **Rb** > **Ra**, **Rc**. Interestingly, although similar reaction mechanisms (the aromatization) were used for series 1 and series 2, series 1 (coumarin derivatives) was more active than series 2 (chromone derivatives) toward various ROS. This indicates that both the reactive group and the structure of the chosen fluorophore affected the reactivity of the probes. This provides the opportunity to further optimize the reactivity of these probes via structural modification. Unfortunately, the non–redox-based probes reacted with almost all ROS without any clear correlation to the oxidizing ability of the ROS. This broad ROS reactivity obviously limits the application of these types of probes.

### Exploration of Detection Selectivity and Applicability of the Redox Based O_2_^•−^ Probes

Next, we explored the selectivity profiles of redox-based O_2_^•−^ probes (series 1 and 2). The selectivity of the probe was not only determined by the reactivity but also affected by the transformation rate. The transformation rate can be manipulated by adjusting ROS concentration, reaction temperature, and others*.* For example, in this study, we found that chromone-derived probe **Rb** can only react with O_2_^•−^ but not with other ROS although we further increased the ROS concentration, reaction time, and temperature. Thus, probe **Rb** was highly specific to O_2_^•−^. On the other hand, when we balanced the conditions of ROS concentration, and reaction temperature, the transformation rate between the probe and certain ROS was subsequently changed. If we control the transformation rate to allow the number of reaction products to be below or above the detection line, we can achieve detection selectivity. In this study, we proved the reactivity between **R3** and ClO^−^/O_2_^•−^. However, if we set the incubation time for less than 5 min at 37°C, probe **R3** can only turn the fluorescence on by O_2_^•−^ but not by ClO^−^ (ESI S1.5). Thus, probe **R3** can selectively detect O_2_^•−^ under certain conditions.

**FIGURE 4:**
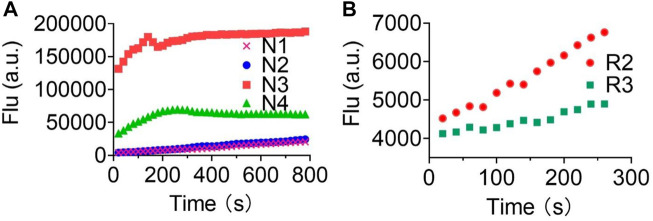
Fluorescence responses of various probes in the XO/HPX system. **(A)**, non–redox-based O_2_^•−^ probes: **N1**, **N2**, **N3**, and **N4**. **(B)**, redox-based O_2_^•−^ probes: **R2** and **R3**. Time course for the change in fluorescence intensity observed with various probes. 50 μM various probes **N1**, **N2**, **N3** and **N4** were dissolved in 0.1 mM HEPES buffer in 0.25 U/ml XO and 1 mM HPX, pH 7.4. 50 μM various probes **R2** and **R3** were dissolved in 0.1 mM HEPES buffer in 0.6 U/ml XO and 1 mM HPX, pH 7.4. Fluorescence intensity was measured with the preference *E*_x_ and *E*_m_ of the probes.

Lastly, we explored the applicability of our redox-based O_2_^•−^ probes in a biological system. The O_2_^•−^ was produced via the more biologically relevant xanthine oxidase (XO)/hypoxanthine (HPX) system (Figure 4). We began this study at a relatively low concentration of XO enzyme (0.25 U/ml) and 1 mM HPX. We observed a real-time fluorescence change for non–redox-based probes **N1**, **N2**, **N3**, and **N4**. The sulfonyl ester series (**N3**, **N4**) was particularly active under the XO/HPX system, and the fluorescence quickly reached a peak level after several minutes. While the boronate series (**N1**, **N2**) was successful, it was much slower than the sulfonyl ester series (Figure 4A). However, under this enzyme condition, we did not observe a fluorescence change for all redox-based O_2_^•−^ probes (series 1 and 2). We further increased the concentration of the XO enzymes (0.6 U/ml) to produce more concentrated O_2_^•−^ in the system. Then, we observed a slow fluorescence increase for probes **R2** and **R3** (Figure 4B), but no fluorescence change was observed for probes **R1**, **Ra**, **Rb**, and **Rc**.

Previous studies have shown that O_2_^•−^ reacts violently with H_2_O (Wang et al., 2020; Tampieri et al., 2019). Thus, in the XO/HPX system, the majority of the produced O_2_^•−^ will react with excessive H_2_O before they can reach the probes. The reaction between O_2_^•−^ and H_2_O produces H_2_O_2_ and OH- (Tampieri et al., 2019), both of which remain relatively stable in the aqueous solution. This will eventually cause the solution contain high concentrations of H_2_O_2_ and OH-, but rather low concentrations of freshly produced O_2_^•−^. Interestingly, we proved that non–redox-based O_2_^•−^ probes can react with almost all oxidizing levels of ROS (Table 2). Thus, both newly produced O_2_^•−^ and the degraded low oxidizing ROS can react with non-redox O_2_^•−^ probes to turn on the fluorescence. Notably, we also showed that probes of series 4 (the sulfonyl ester) but not series 3 (the boronate) can react with OH- to turn on the fluorescence (Figure 3) (Tampieri et al., 2019), this was accordingly reflected as probes of series 4 were more reactive than those of series 3 in the XO/HPX system (Figure 4A). By contrast, in the XO/HPX system, only O_2_^•−^ but not other low oxidizing ROS can turn on the fluorescence of redox-based O_2_^•−^ probes (series 1 and 2). Thus, redox-based O_2_^•−^ probes reacted more slowly toward O_2_^•−^ in the XO/HPX system (Figure 4). When we further increased the concentration of the XO enzyme, a small portion of **R2** and **R3** slowly turned on the fluorescence. Taken together, we found that in the XO/HPX system, non–redox-based O_2_^•−^ probes were more active than redox-based ones. Unfortunately, most of the non–redox-based O_2_^•−^ probes unselectively turned on fluorescence by the low oxidizing level of ROS. By contrast, redox-based O_2_^•−^ probes **R2** and **R3** can only be slowly oxidized by O_2_^•−^ to turn on fluorescence, and the fluorescence change was directly caused by O_2_^•−^ but not other low oxidizing ROS. In summary, we showed the advantage of redox-based O_2_^•−^ probes in the detection of O_2_^•−^ in a biological system.

## Conclusion

In this study, we explored the difference between redox- and non–redox-based superoxide anion probes. We found that redox-based probes showed clear detection preference correlating with the oxidation ability of the ROS, with non–redox-based probes reacting unselectively with a range of ROS. This indicated that further efforts to develop O_2_^•−^ sensors should pay attention to the redox-based mechanism. Interestingly, for the same type of redox-based probe, the detection selectivity toward superoxide anion can be optimized through the modification of the structure of the fluorophore, which will eventually provide the community with sensitive and highly selective sensors for O_2_^•−^.

## Data Availability

The fluorescence responses of all reactions, the LC-MS characterizations of the reactions, ^1^H and ^13^C NMR spectra for all final compounds can be found in the Supplementary Material.
