# Supplementary material for: Insights Into the Detection Selectivity of Redox and Non-redox Based Probes for the Superoxide Anion Using Coumarin and Chromone as the Fluorophores
Source: Front Chem. 2021 Nov 25;9:753621. doi: 10.3389/fchem.2021.753621 (PMC8667960; doi:10.3389/fchem.2021.753621)
Supplement: Supplementary file 1 [file Table1.DOCX]

Insights into the detection selectivity of redox and non-redox based probes for the superoxide anion using coumarin and chromone as the fluorophores

Yuchen Wang‡, Shumi Jia‡, Zhenyan Yu, Hui Wen, Huaqing Cui*

State Key Laboratory of Bioactive Substances and Function of Natural Medicine, Institute of Materia Medica, Peking Union Medical College and Chinese Academy of Medical Sciences. 1 Xiannongtan Street, Beijing, 100050, China

* Correspondence: [hcui@imm.ac.cn](mailto:hcui@imm.ac.cn) (H.C.)

‡ These two authors contributed equally to this study.

S1, Determination of the reactivity between fluorescent probes and various ROS

Various ROS was also prepared as literature in 0.1 M phosphate buffer, 0.15 M NaCl, pH 7.4 or anhydrous DMSO [1, 2]. Each probe was dissolved in these ROS solution as the final concentration of 10 μM. After incubated at 37 ^o^C for 5 minutes, the mixture was scanned the preferred *E*_x_ of desired fluorophore to check if the desired fluorophore were formed. In order to detect the fluorescence change, the *E*_x_ was set as 340nm, the *E*_m_ was measured between 380 to 600 nm.

ROS agents: [*tert*-butyl hydroperoxide](https://pubchem.ncbi.nlm.nih.gov/compound/Tert-butyl-hydroperoxide) : TBHP, H_2_O_2_, •OH, ^1^O_2_, ClO^-^, O_2_^•−^.

**S1.1** H_2_O_2_: Purchased commercial available H_2_O_2_ (~8.8 M) was used and dissolved in 0.1 M phosphate buffer, 0.15 M NaCl, pH 7.4 as the final concentration of 100 μM. Probe 1 was added into the solution as the final concentration of 10 μM. The reaction was vortexed for 10 s, and incubated for 5 min at 37^o^C for measurement. If there is no desired fluorescence found, the concentration of H_2_O_2_ will be increased to 1mM and 20mM. Black line stands for the fluorescence of the control, the red line shows the spectra of the reaction mixture.

**100 μM H_2_O_2_**


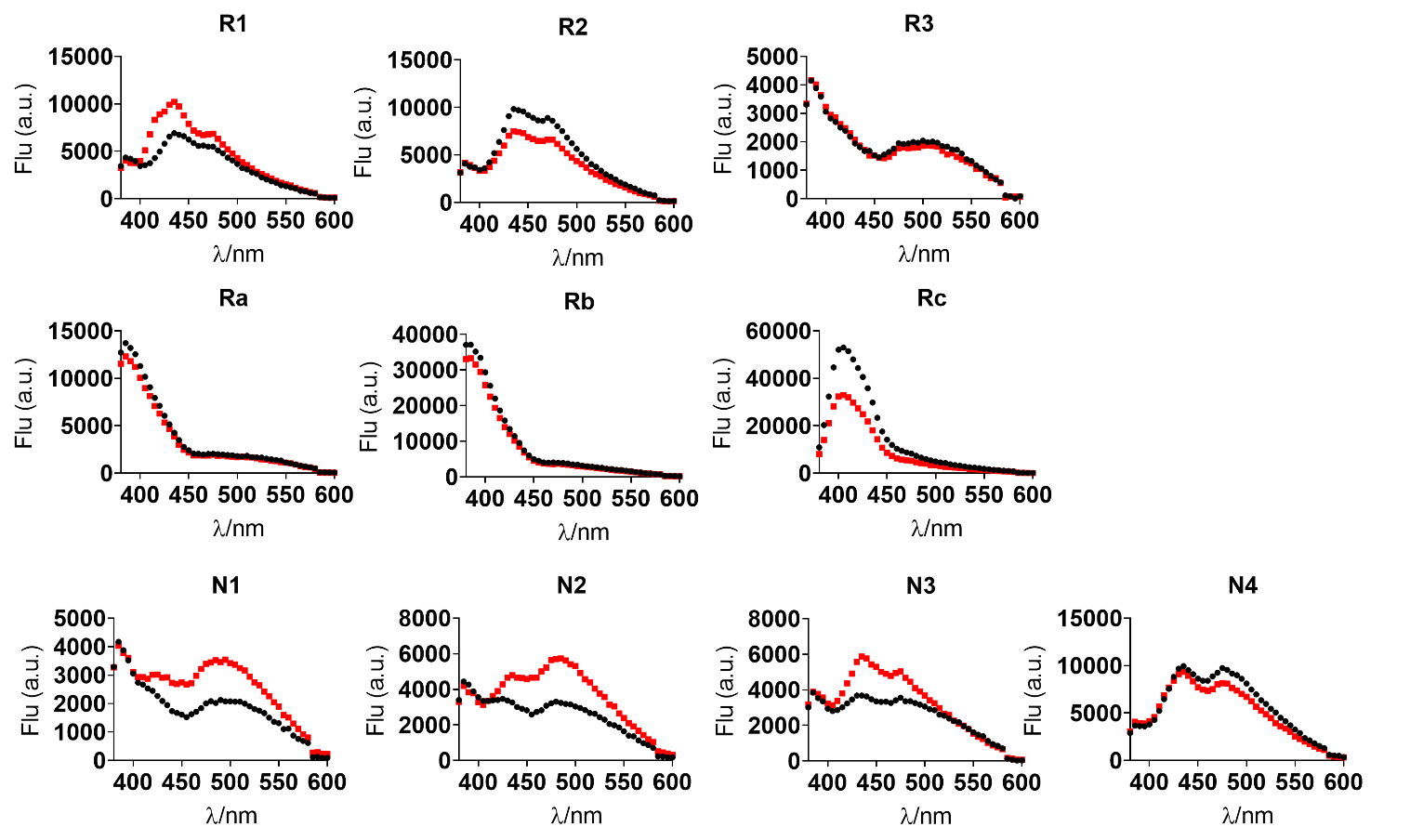


**1 mM H_2_O_2_**

_
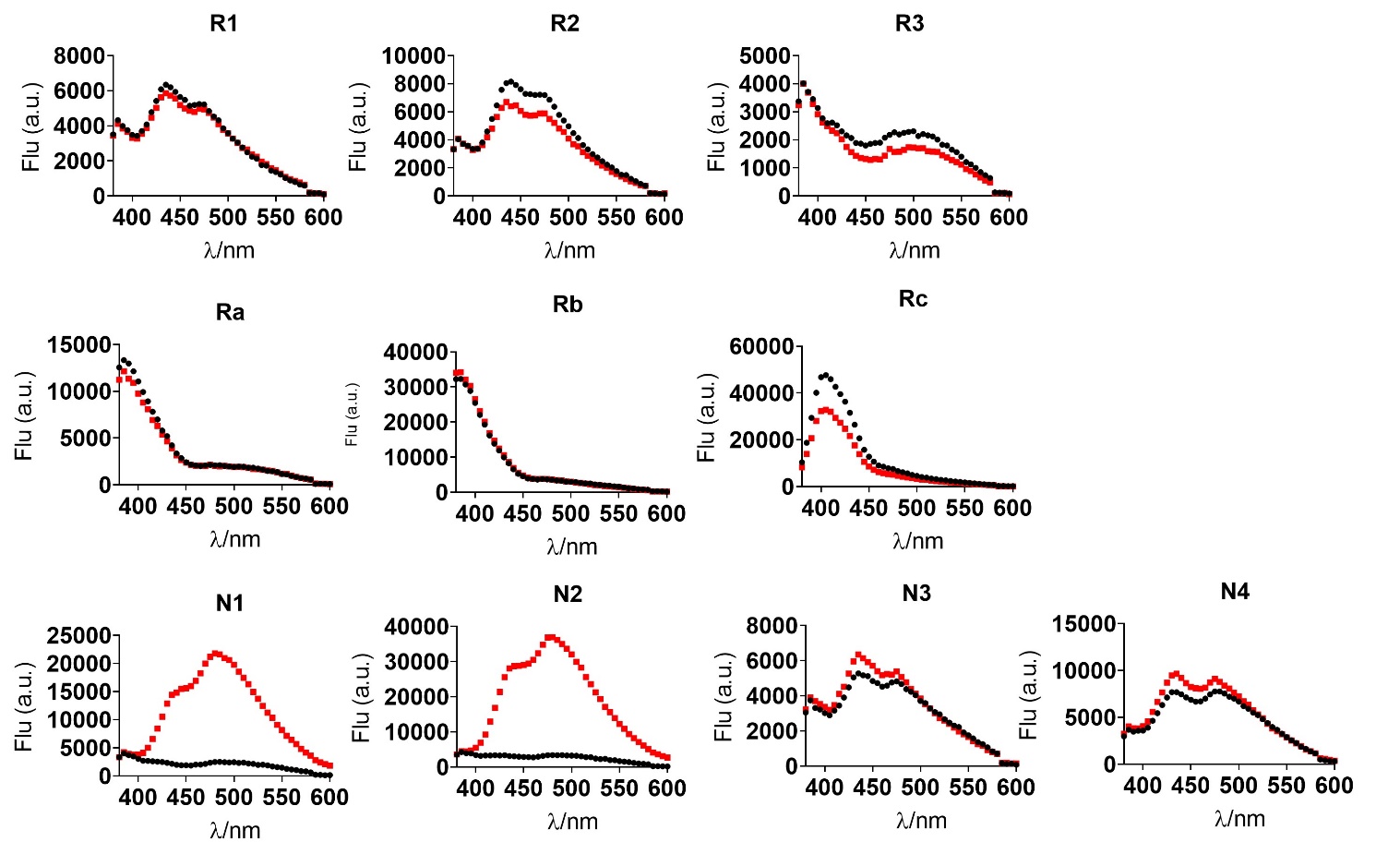
_

**20 mM H_2_O_2_**


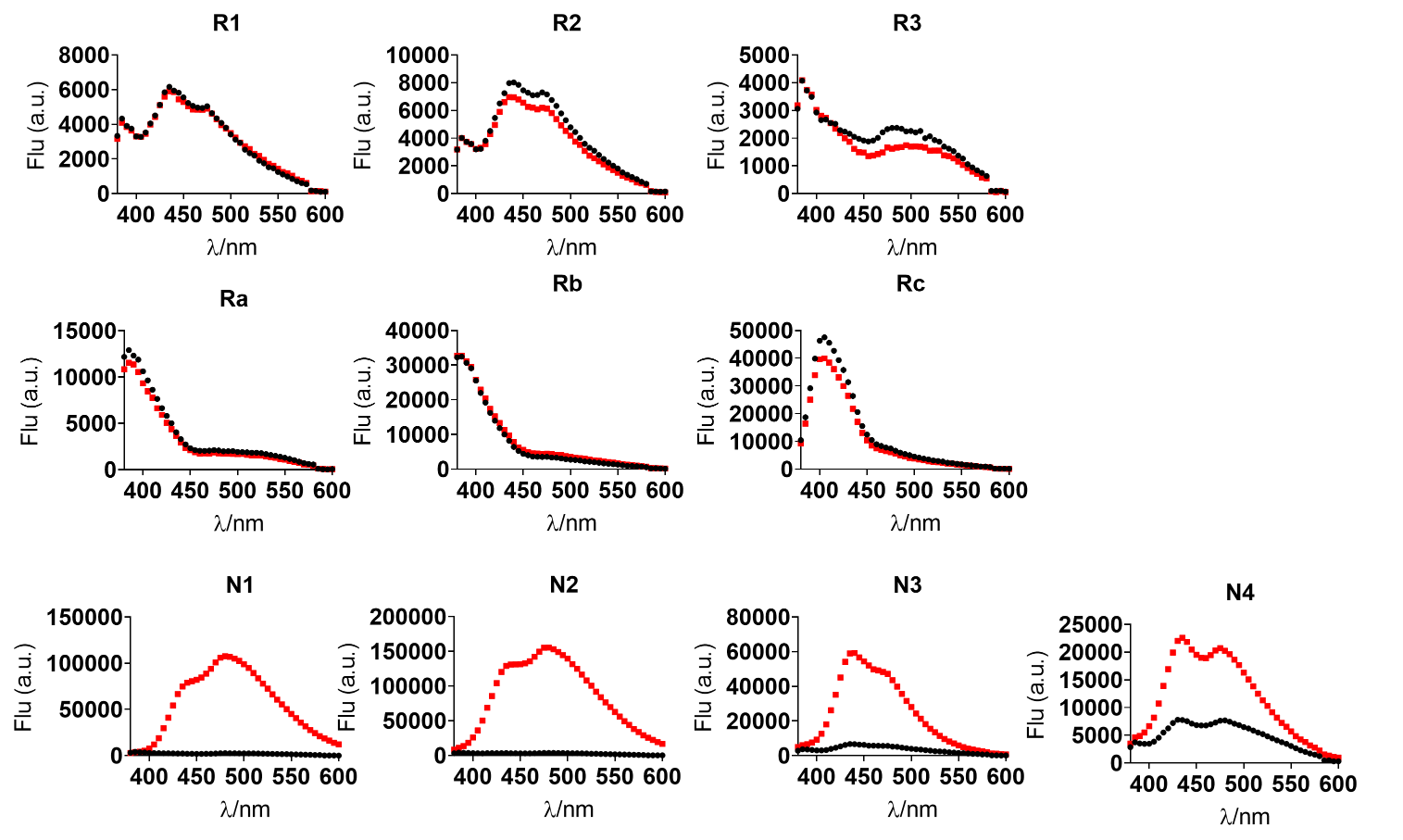


**S1.2** TBHP: Purchased commercial available TBHP (~6.0 M) was used and dissolved in 0.1 M phosphate buffer, 0.15 M NaCl, pH 7.4 as the final concentration of 100 μM. Probe 1 was added into the solution as the final concentration of 10 μM. The reaction was vortexed for 10 s, and incubated for 5 min at 37^o^C for measurement. If there is no desired fluorescence found, the concentration of TBHP will be increased to 1mM and 20mM. Black line stands for the fluorescence of the control, the red line shows the spectra of the reaction mixture.

**100μM TBHP**


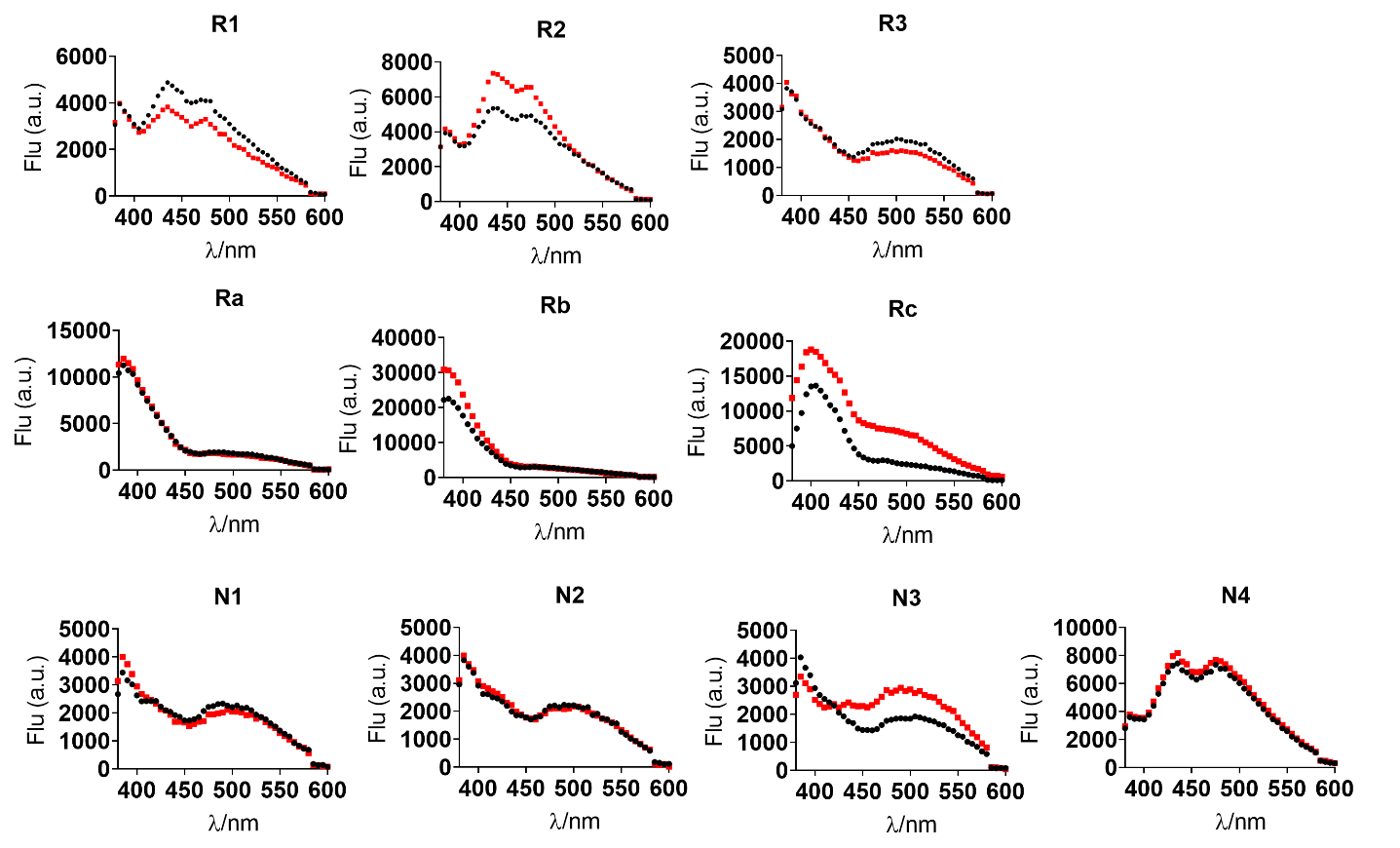


**1mM TBHP**

**
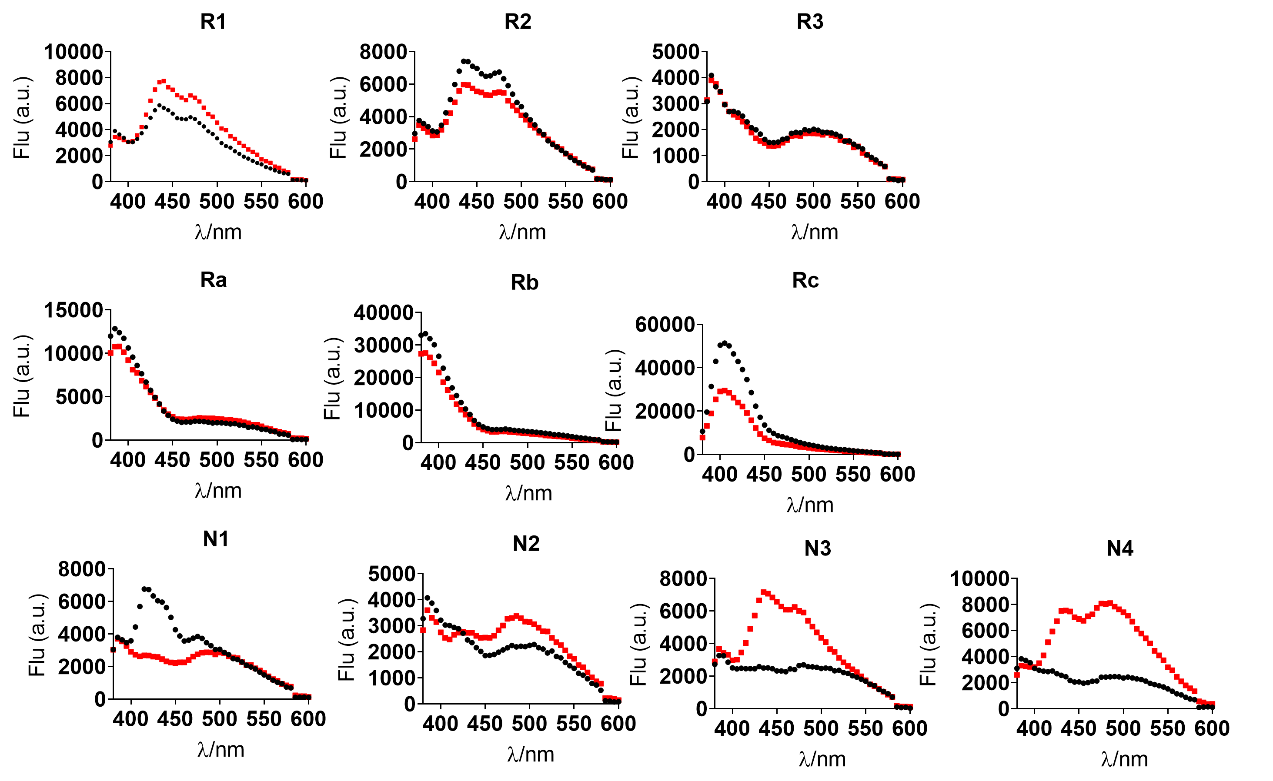
**

**20mM TBHP**


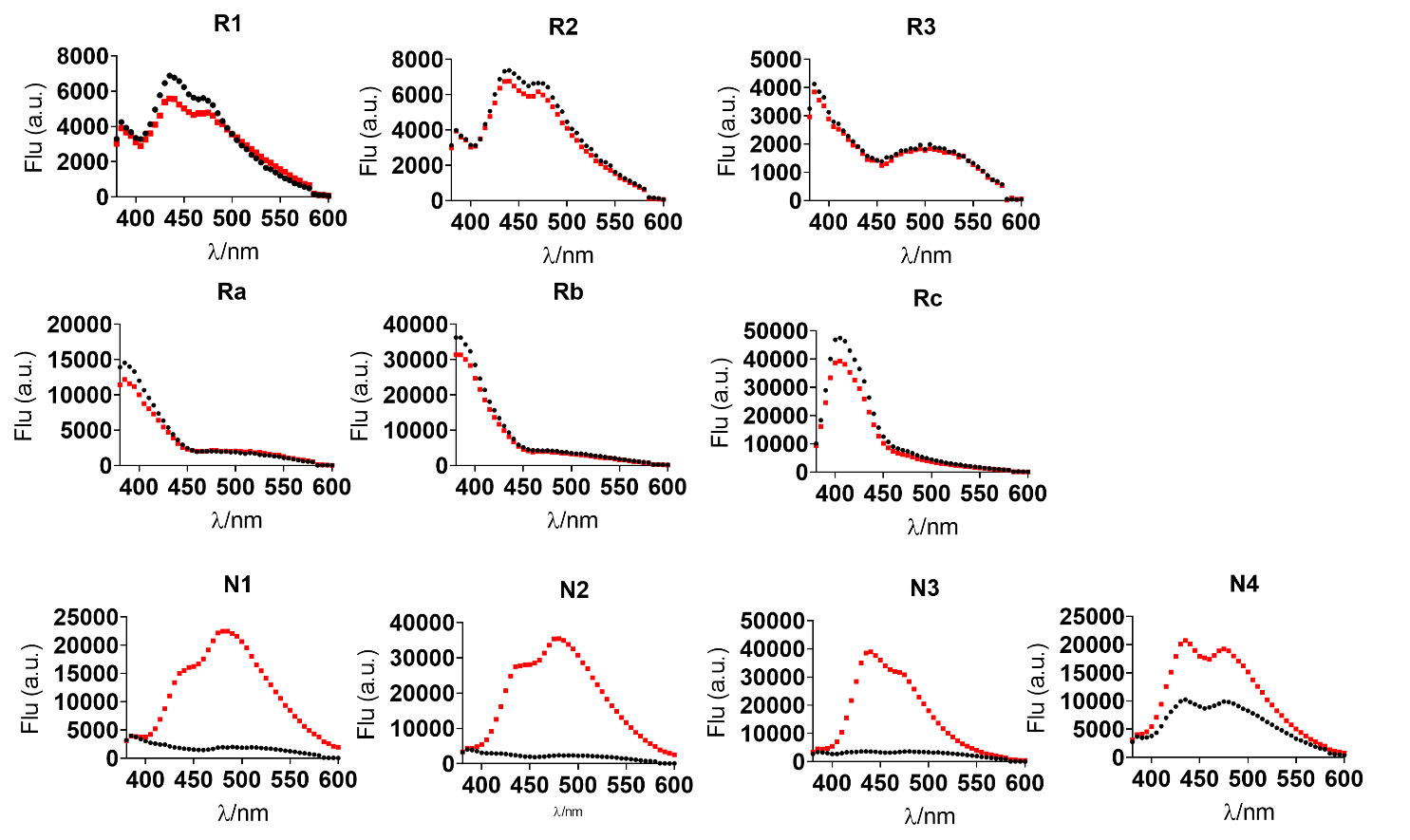


**S1.3** ^1^O_2_: Purchased commercial available NaOCl (~1.4 M) was used and dissolved in 0.1 M phosphate buffer, 0.15 M NaCl, pH 7.4 as the final concentration of 100 μM. Twice dose of H_2_O_2_ was also added to the solution to make the final concentration of 200 μM. The solution was mixed, and probe 1 was added into the solution as the final concentration of 10 μM. The reaction was vortexed for 10 s, and incubated for 5 min at 37^o^C for measurement. However, with increased concentration of OCl^-^, the fluorescent intensity started to decrease, so the detection only performed with 100 μM. Black line stands for the fluorescence of the control, the red line shows the spectra of the reaction mixture.

**100 μM ^1^O_2_**


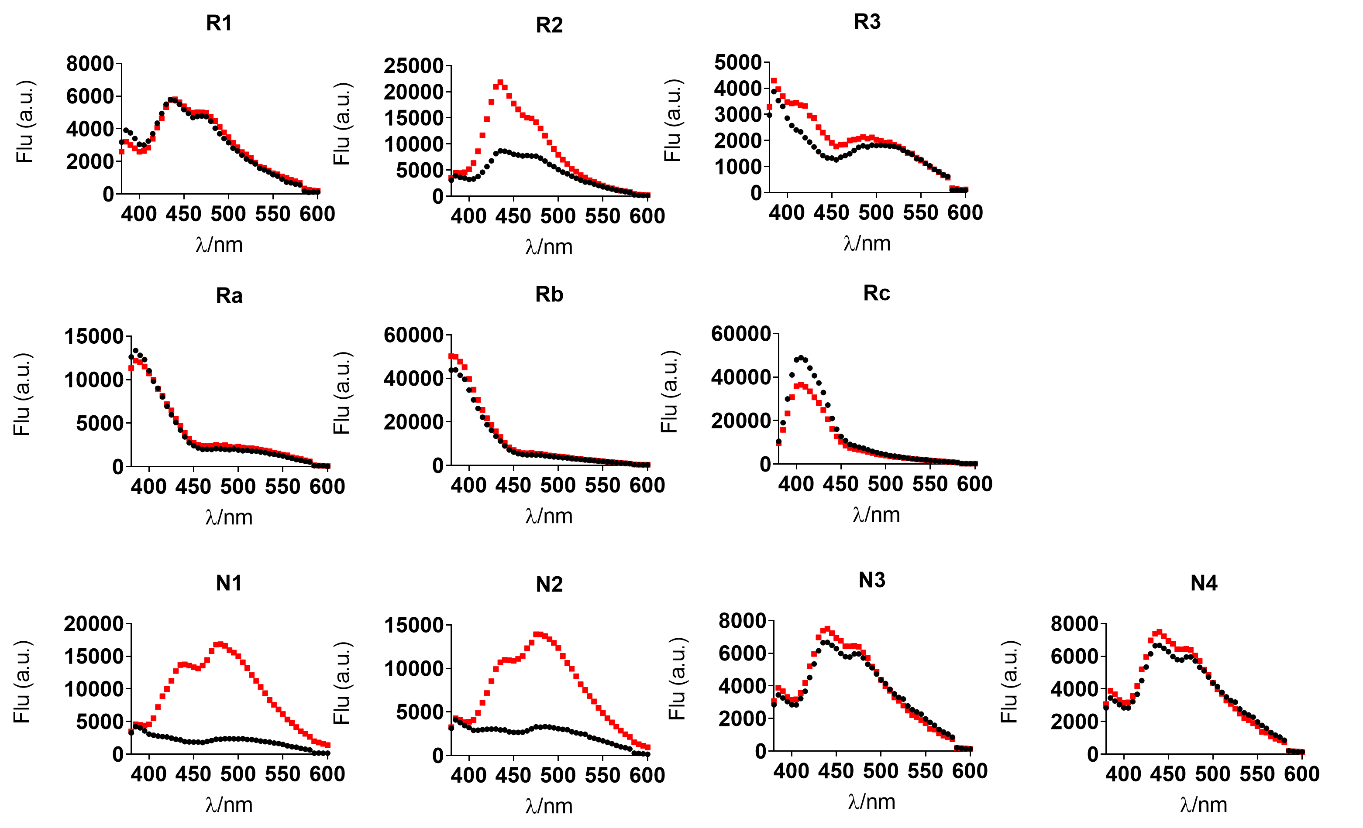


**S1.4** •OH: Purchased commercial available FeSO_4_ was dissolved in 0.1 M phosphate buffer, 0.15 M NaCl, pH 7.4 as the final concentration of 100 μM. Twice dose of H_2_O_2_ was also added to the solution to make the final concentration of 200 μM. The solution was mixed, and probe 1 was added into the solution as the final concentration of 10 μM. The reaction was vortexed for 10 s, and incubated for 5 min at 37^o^C for measurement. If there is no desired fluorescence found, the concentration of •OH will be increased to 1mM. However, when we increased the concentration of FeSO_4_ to 20 mM, the precipitation of FeSO_4_ affect the measurement of the fluorescence. Black line stands for the fluorescence of the control, the red line shows the spectra of the reaction mixture.

**100 μM •OH**


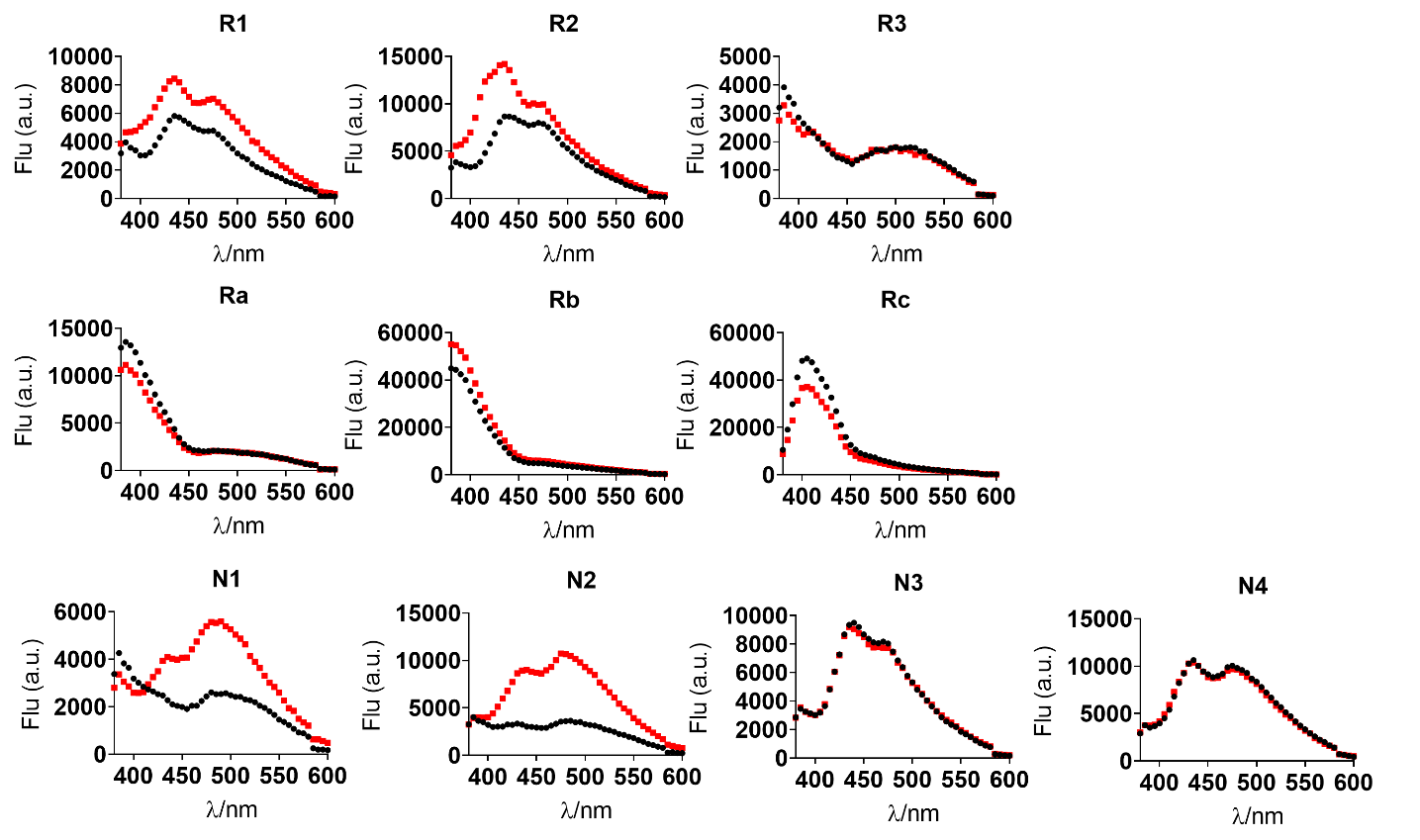


**1mM •OH**


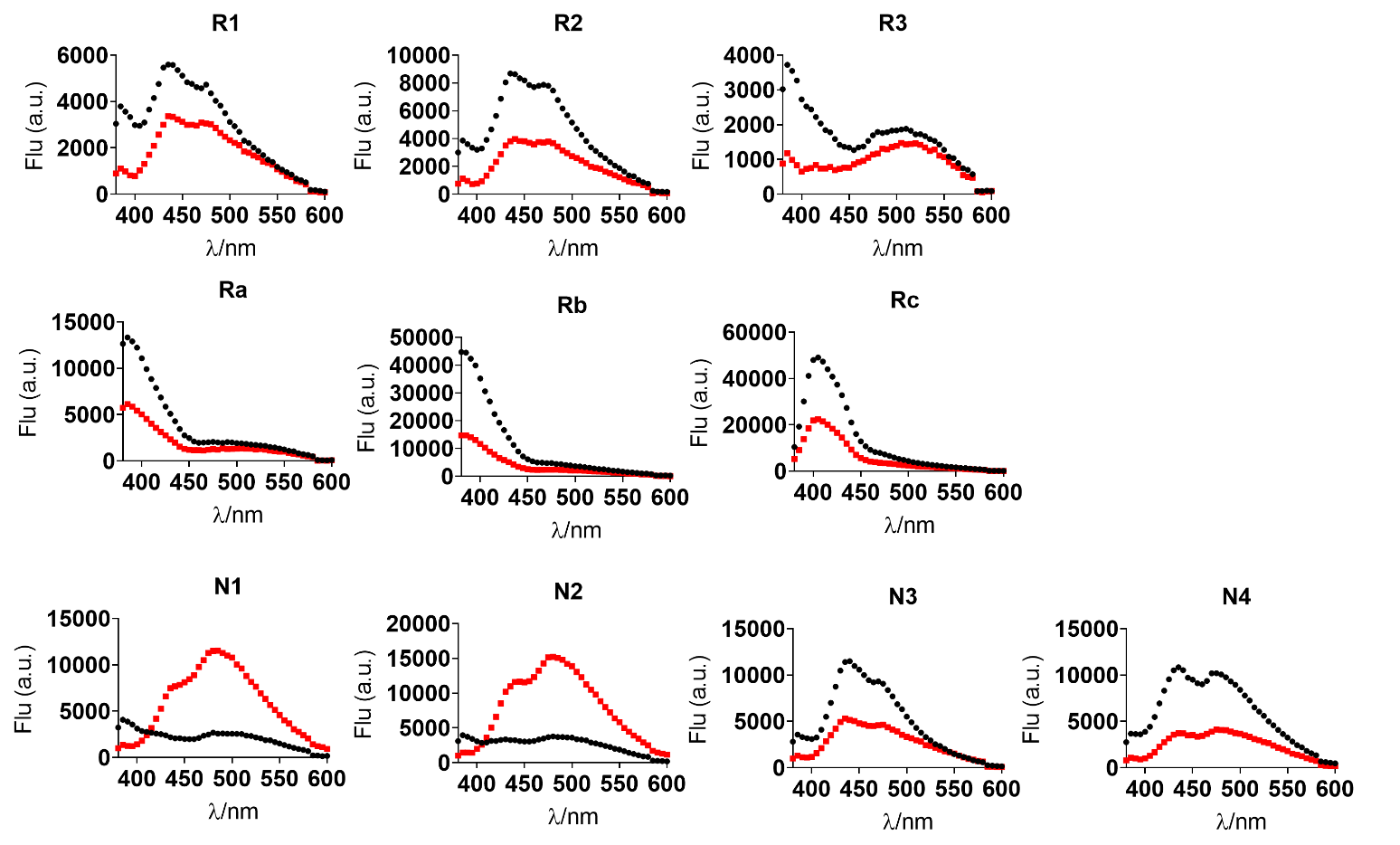


**S1.5** OCl^-^: Purchased commercial available NaOCl (~1.4 M) was used and dissolved in 0.1 M phosphate buffer, 0.15 M NaCl, pH 7.4 as the final concentration of 100 μM. Probe 1 was added into the solution as the final concentration of 10 μM. The reaction was vortexed for 10 s, and incubated for 5 min at 37^o^C. However, with increased concentration of OCl^-^, the fluorescent intensity was measured to be decreased, so the detection only performed with 100 μM of OCl^-^. Black line stands for the fluorescence of the control, the red line shows the spectra of the reaction mixture. Notably, we found that if the incubation was set as exactly 5 minutes, we only observed the fluorescence increase for probe R2, N1, N2. But if we slightly increased the incubation time to 10 minutes, the fluorescence increase of R1 and R3 was also observed. Since we cannot increase the concentration of OCl^-^ to evaluate the reactivity between R1, R3 and OCl^-^, we considered the reactivity between R1, R3 and OCl^-^ were successful.

**100 μM OCl^-^, and incubation for 5 minutes**


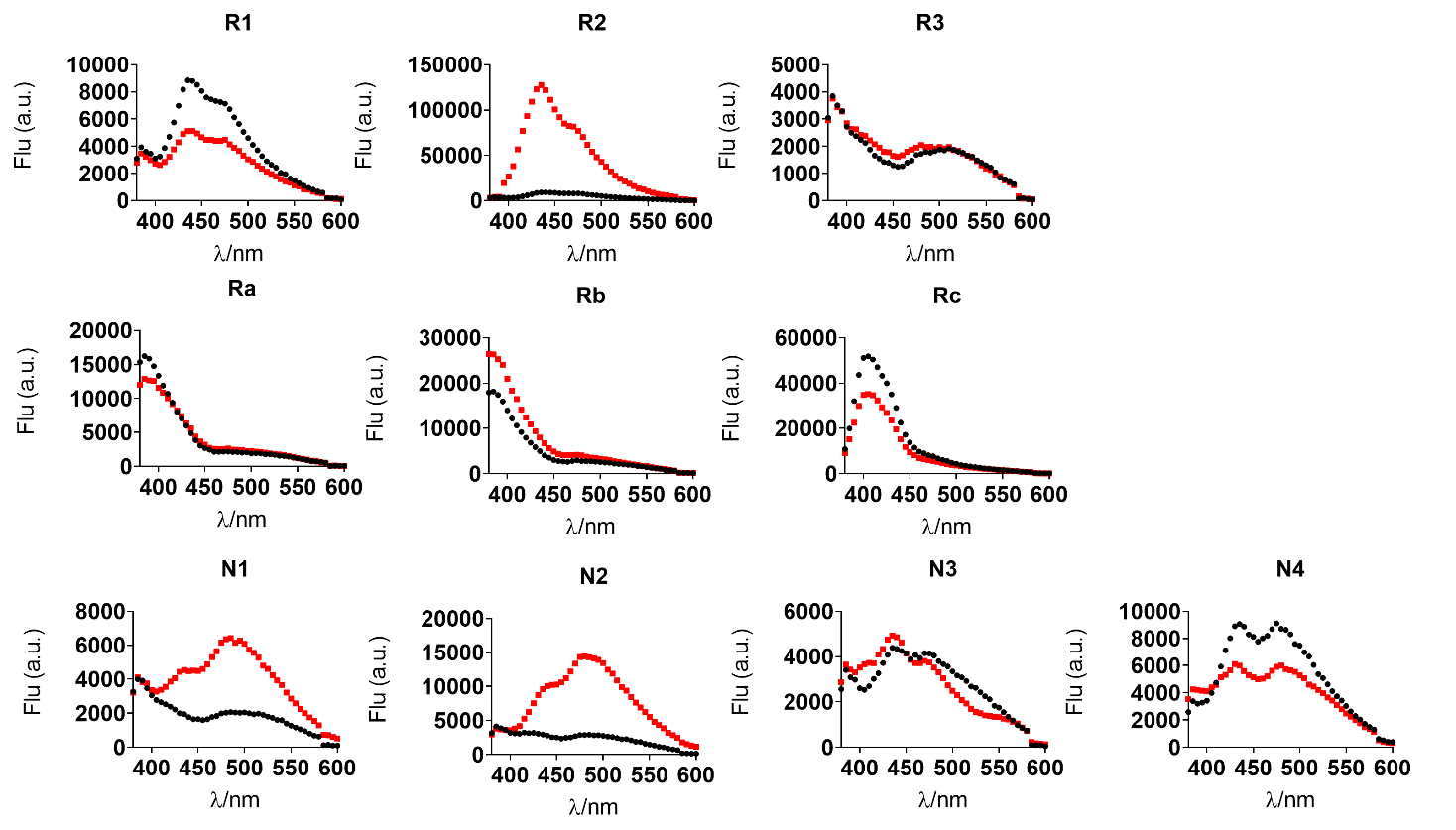


**100 μM OCl^-^, and incubation for 10 minutes**


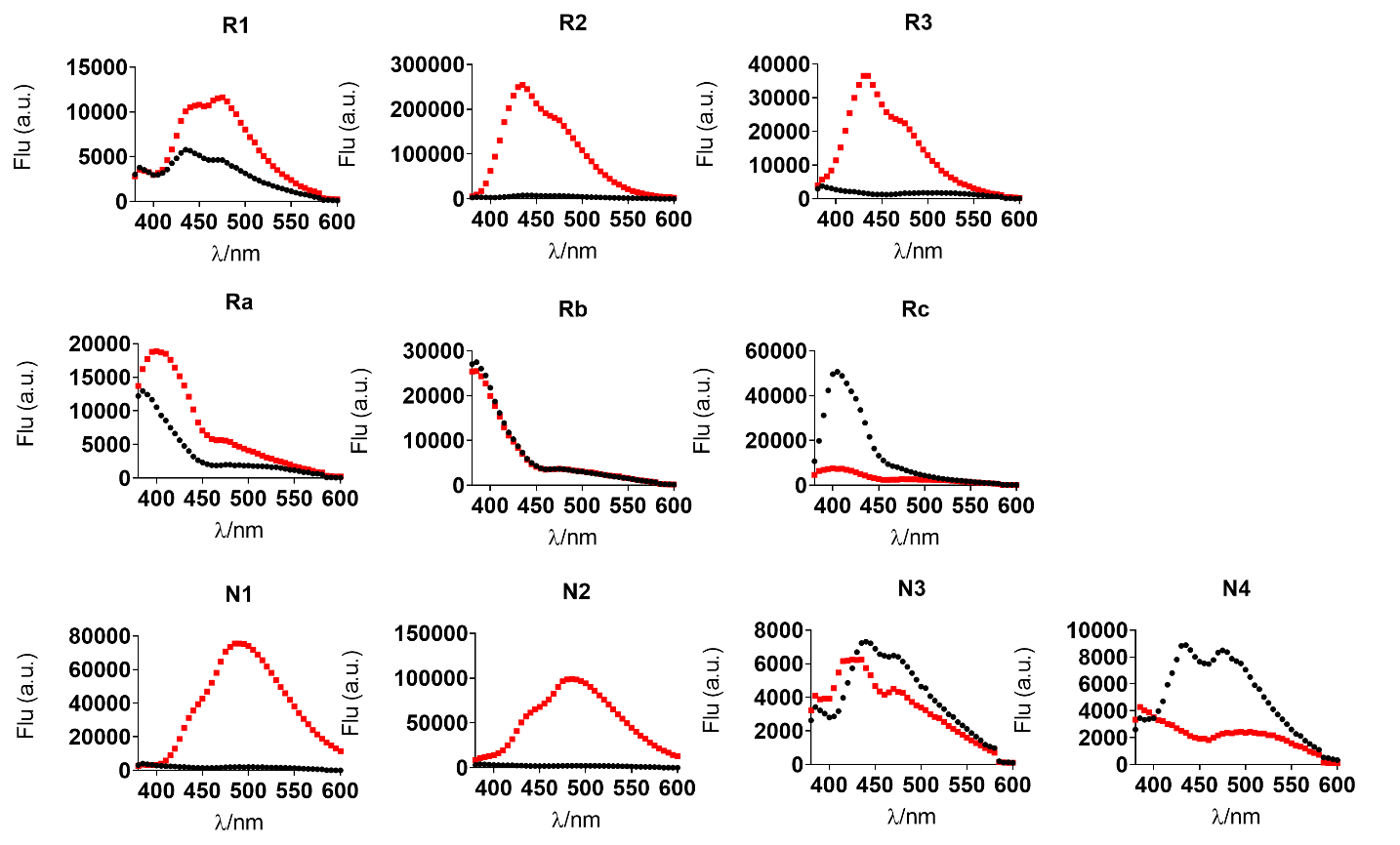


**S1.6** O_2_^•-^: KO_2_ salt was dissolved into anhydrous DMSO to make the final concentration of 100μM, and probe 1 was added into the solution as the final concentration of 10 μM. The reaction was vortexed for 10 s, and incubated for 5 min at 37^o^C for measurement. If there is no desired fluorescence found, the concentration of O_2_^•-^ will be increased to 1mM and 20mM. The fluorescence change of the probes under different concentration of O_2_^•-^ (100μM, 1mM, 20 mM) were compared together to see the reactivity. Moreover, the *E*_x_ for probes of R1, R2 and R3 was set as 370 nm, *E*_x_ for probe Ra, Rb, Rc, N1, N2, N3, N4 were set as 340 nm, the *E*_m_ between 380nm or 400nm to 600 nm were measured.

All **R1**, **R2** and **R3** can react with O_2_^•-^ to turn on the fluorescence.


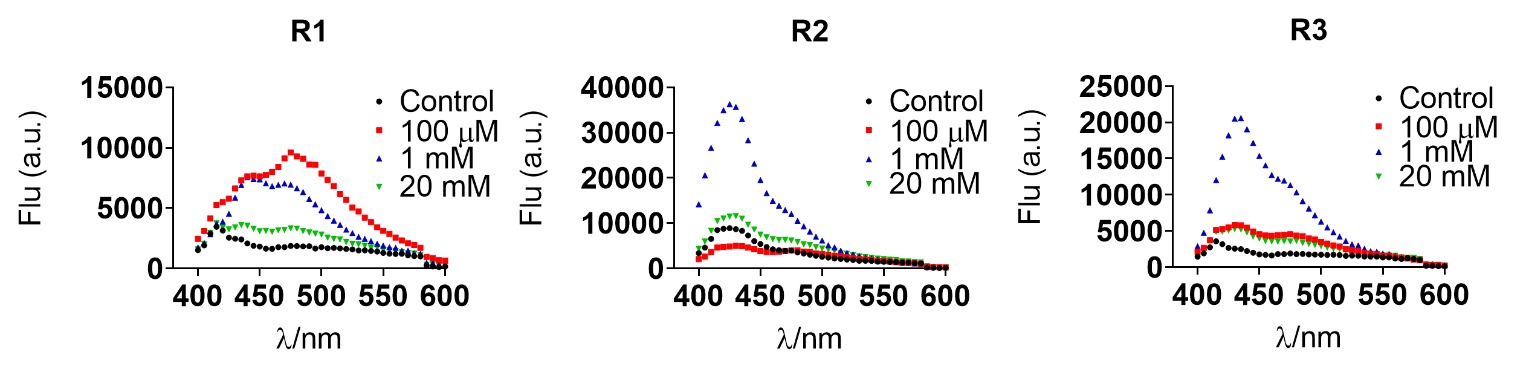


Only **Rb** can react with O_2_^•-^ to turn on the fluorescence.


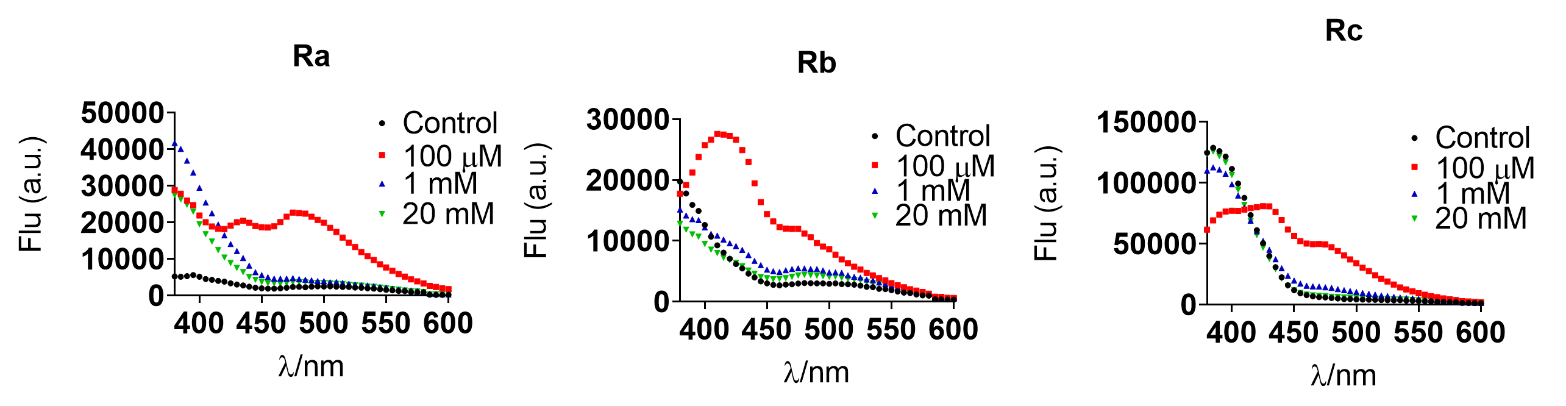


All **N1**, **N2**, **N3** and **N4** can react with O_2_^•-^ to turn on the fluorescence.


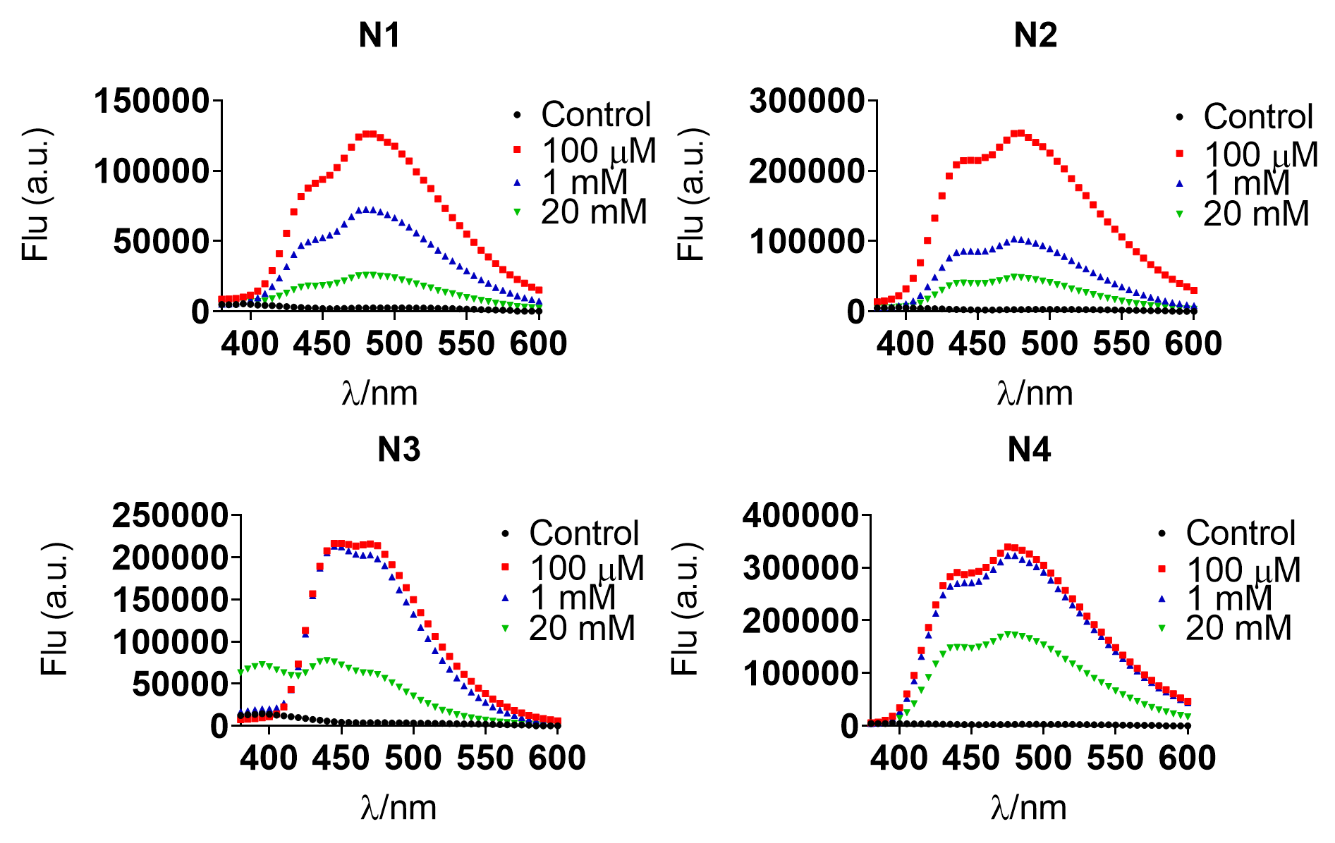


S2, Reaction Characterization by LC-MS

We used LC-MS to confirm the products of the reactions between each probe and various ROS. The MS analysis were carried out and presented wither as [M-H]^-^ or [M+H]^+^.


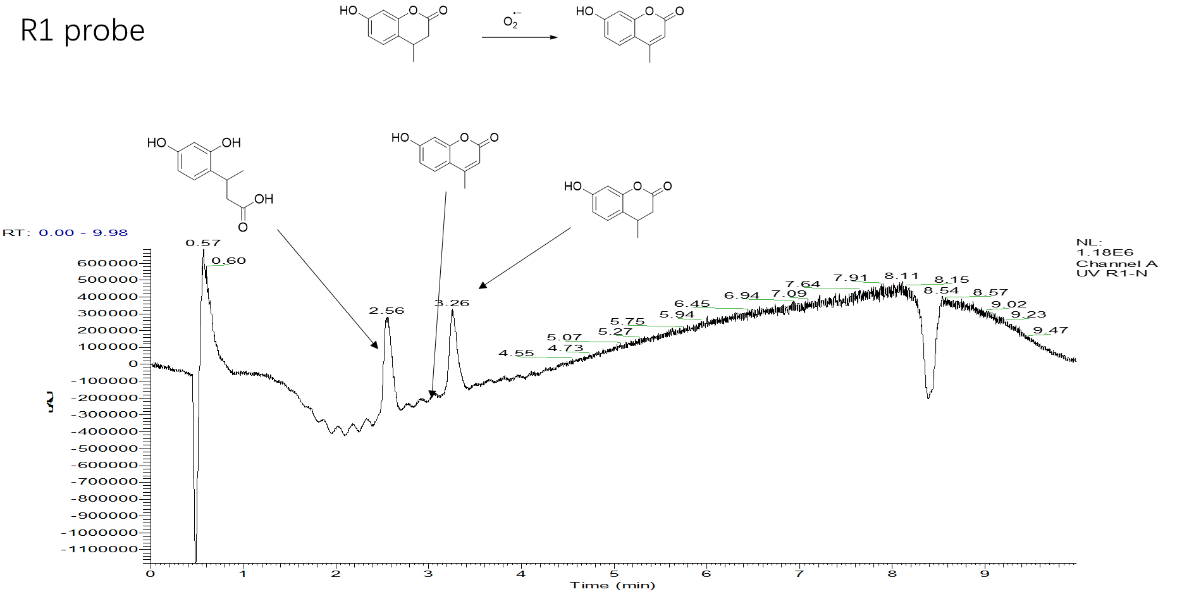


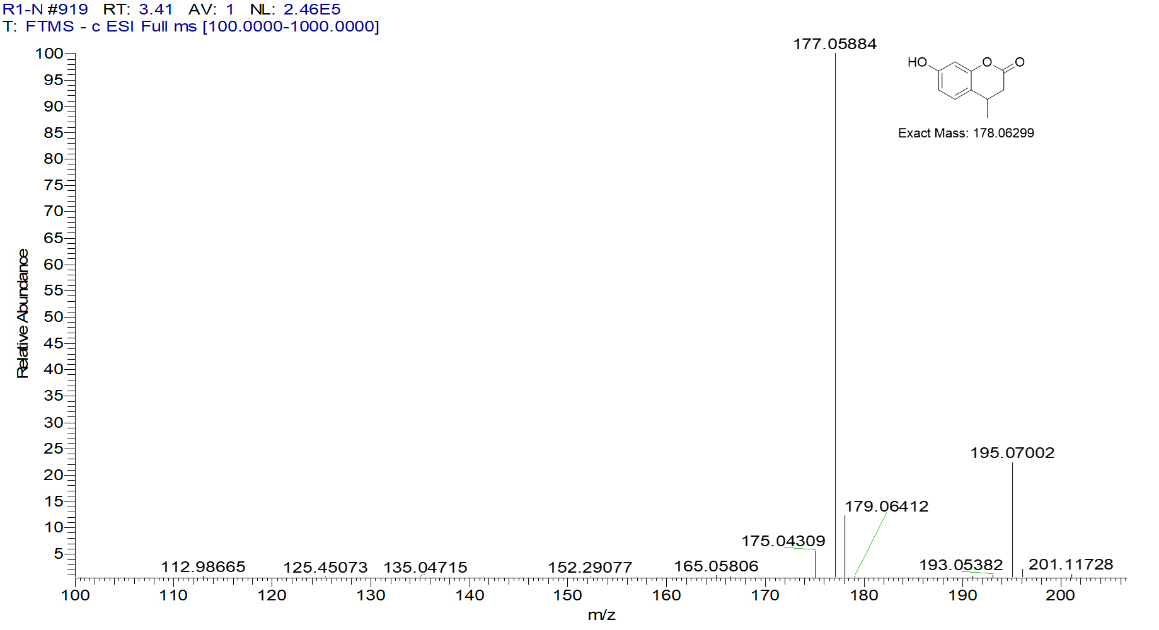


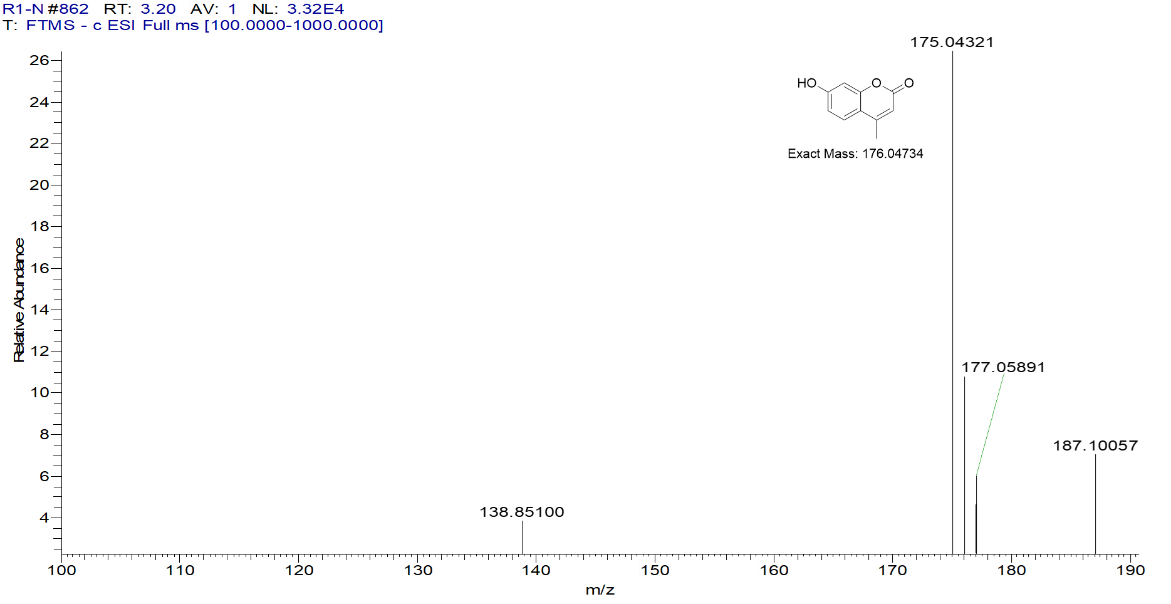


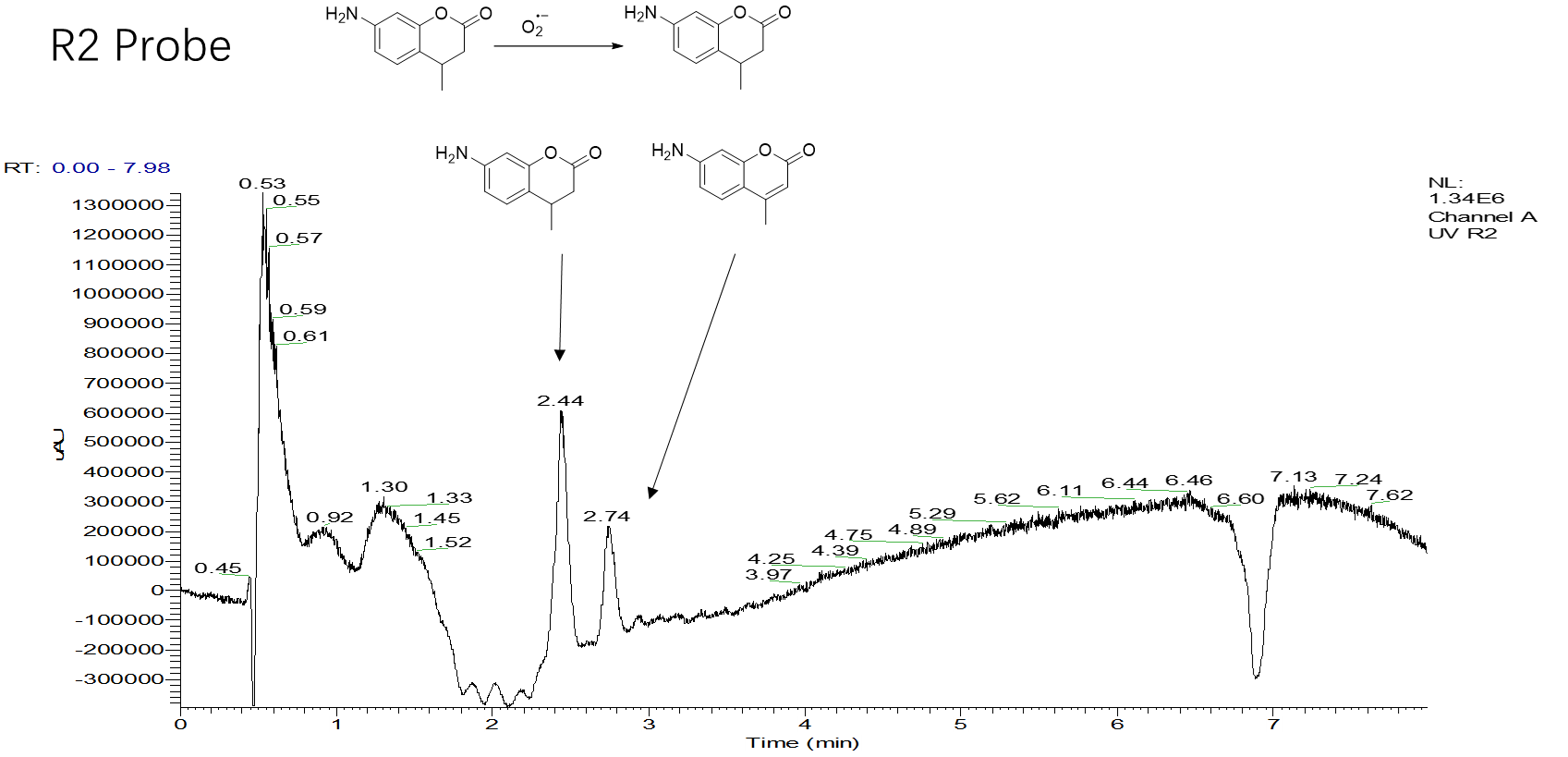


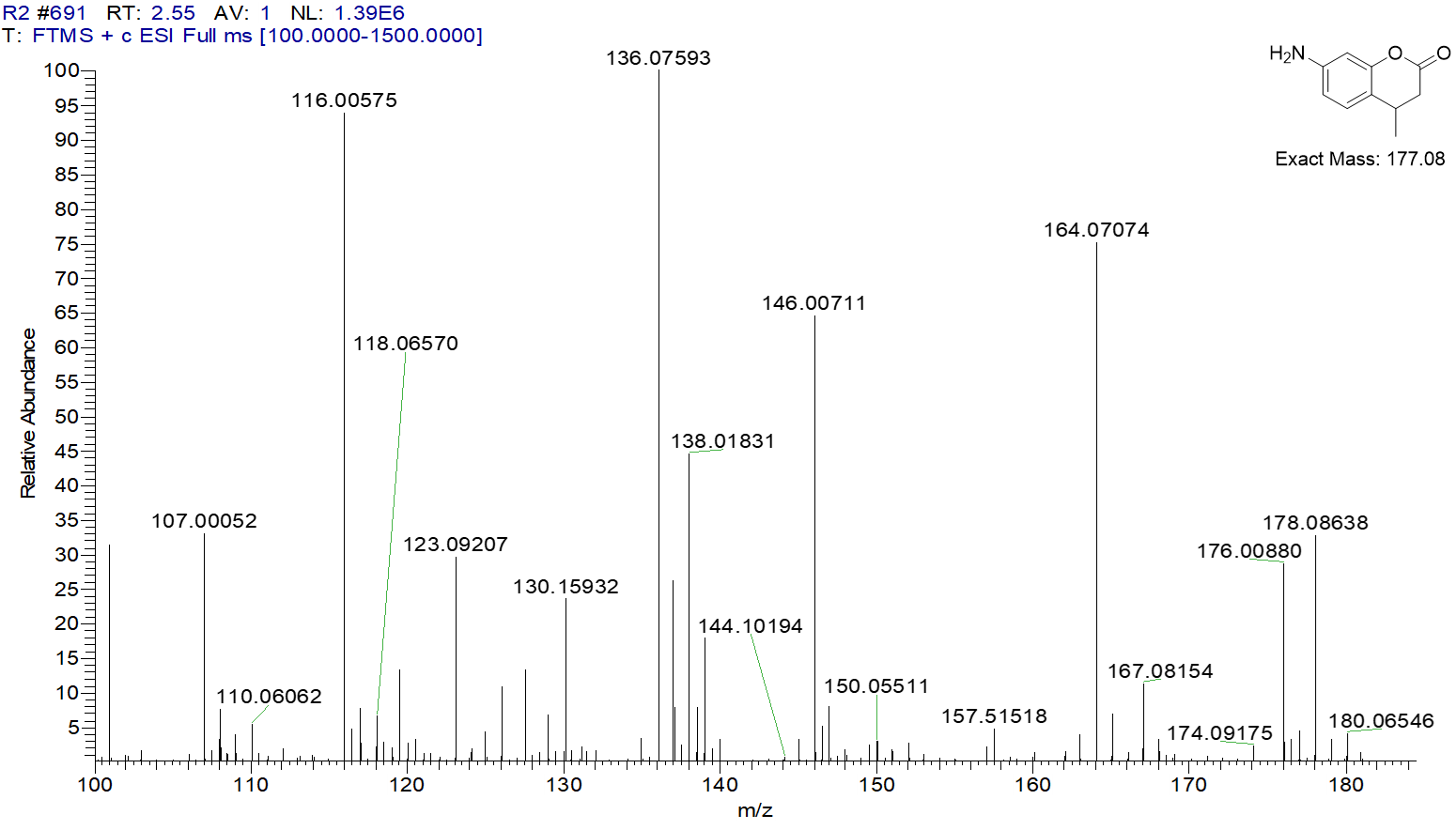


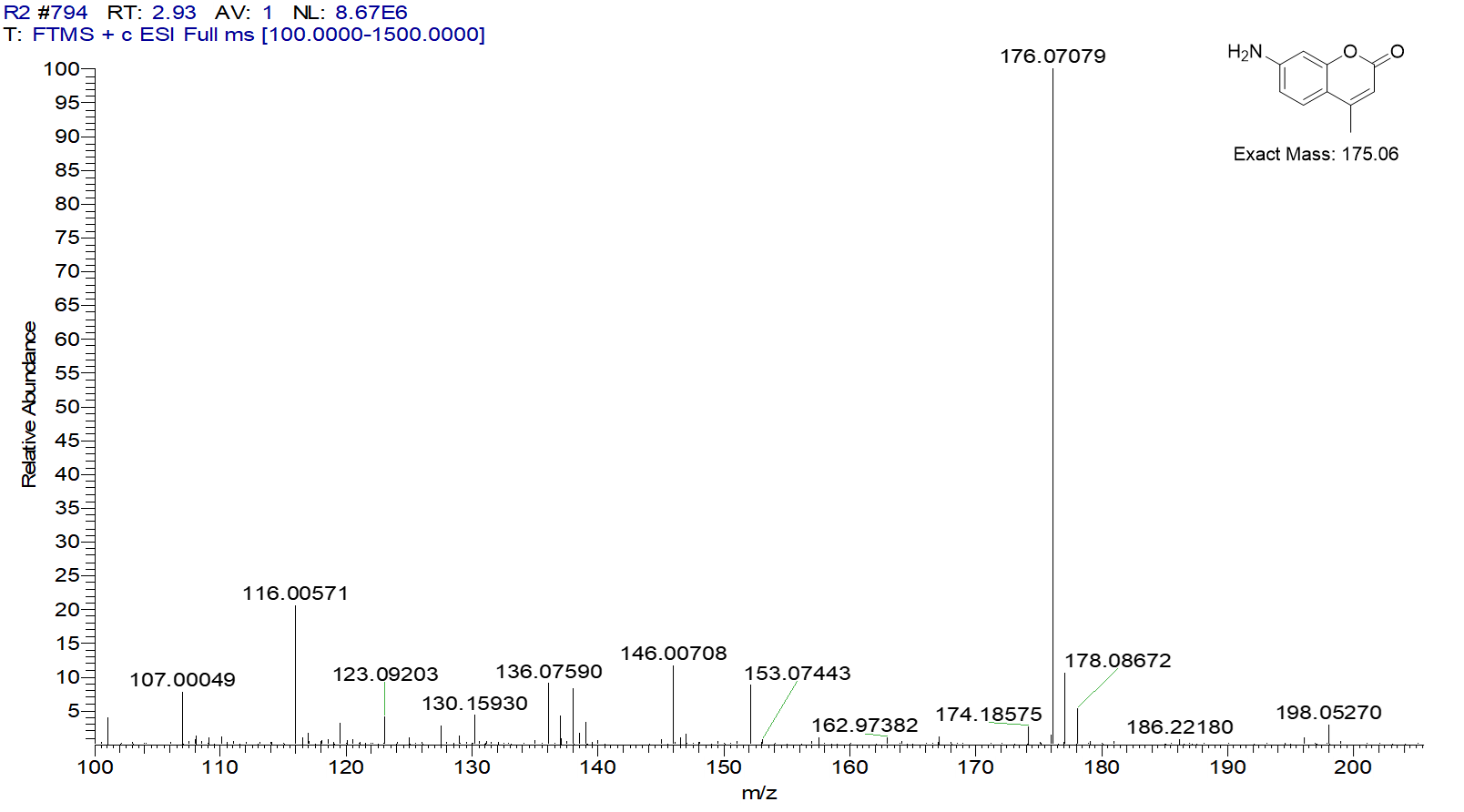


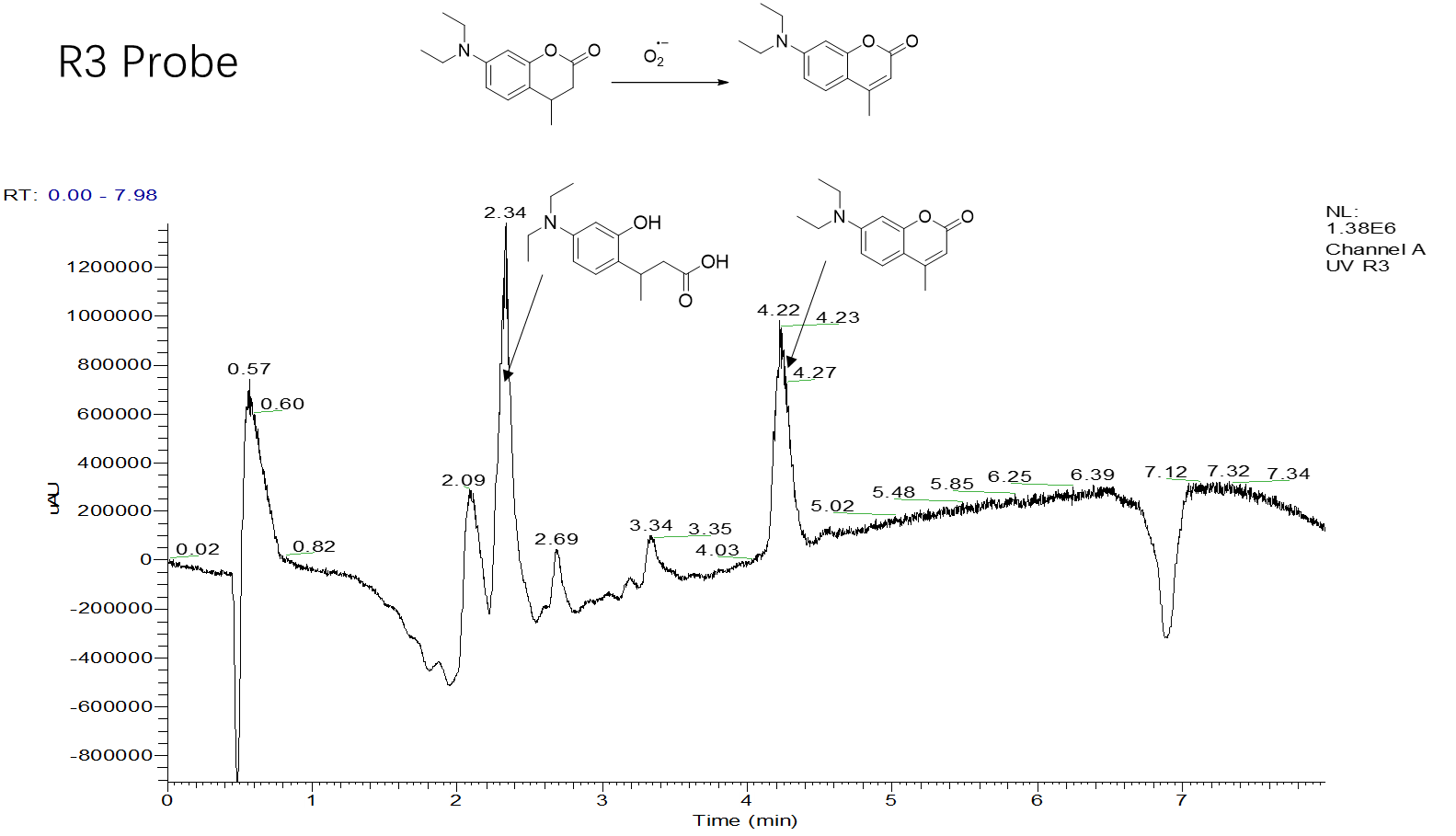


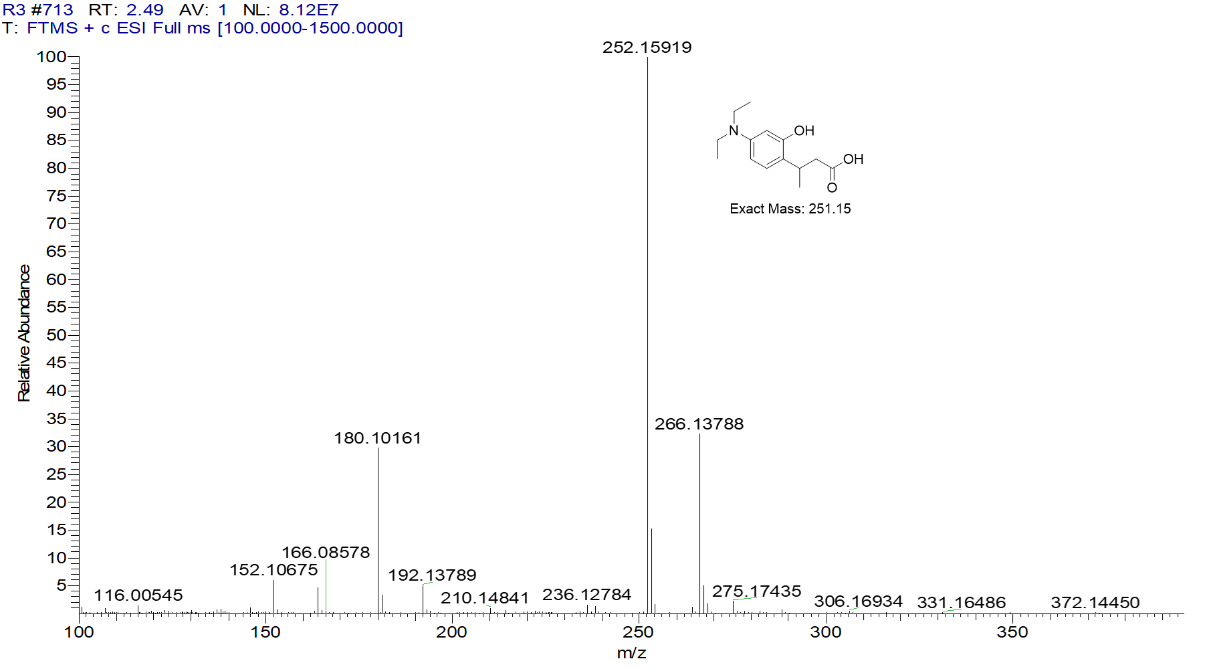


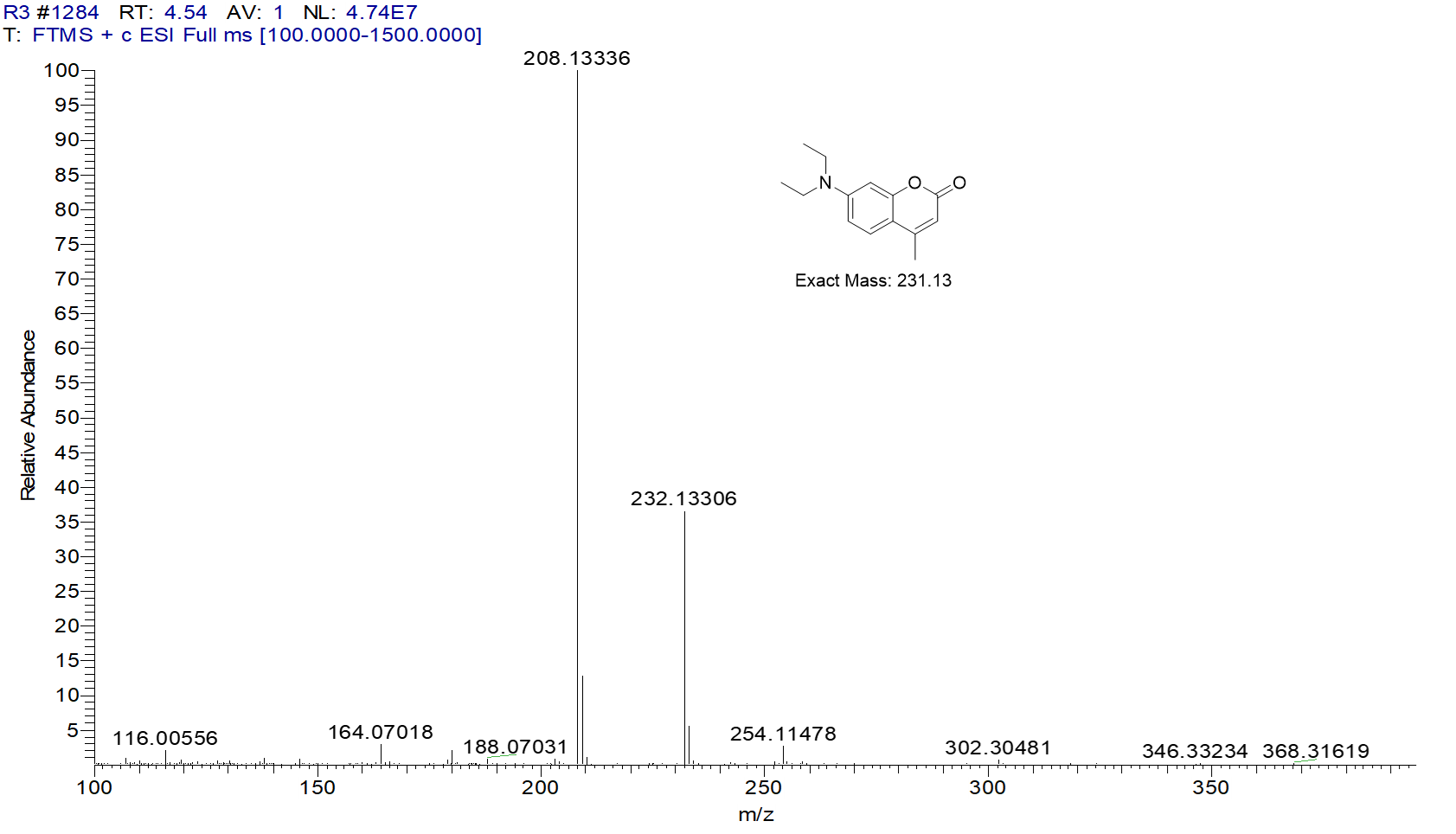


Ra probe:

We did not observe the product between Ra and superoxide anion.


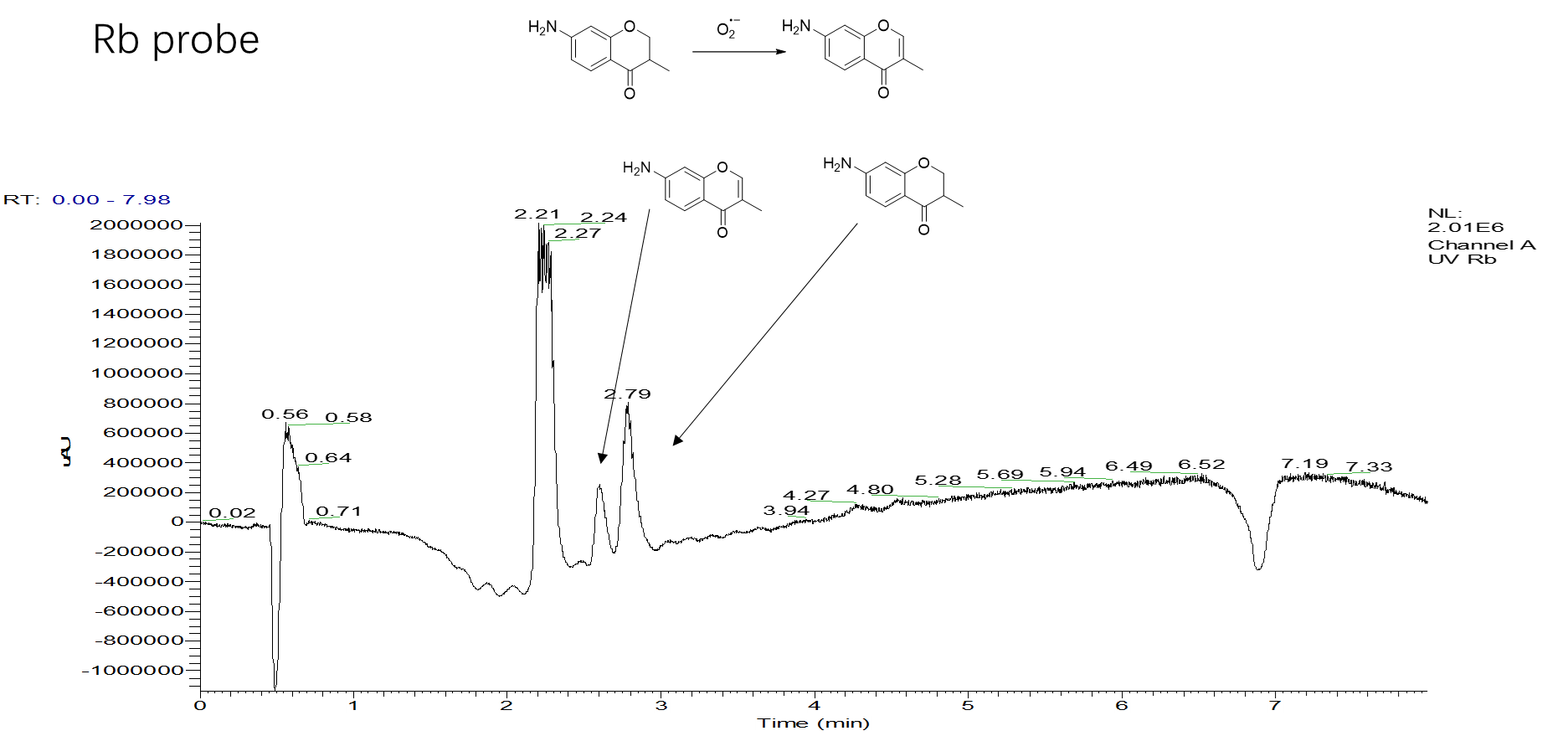


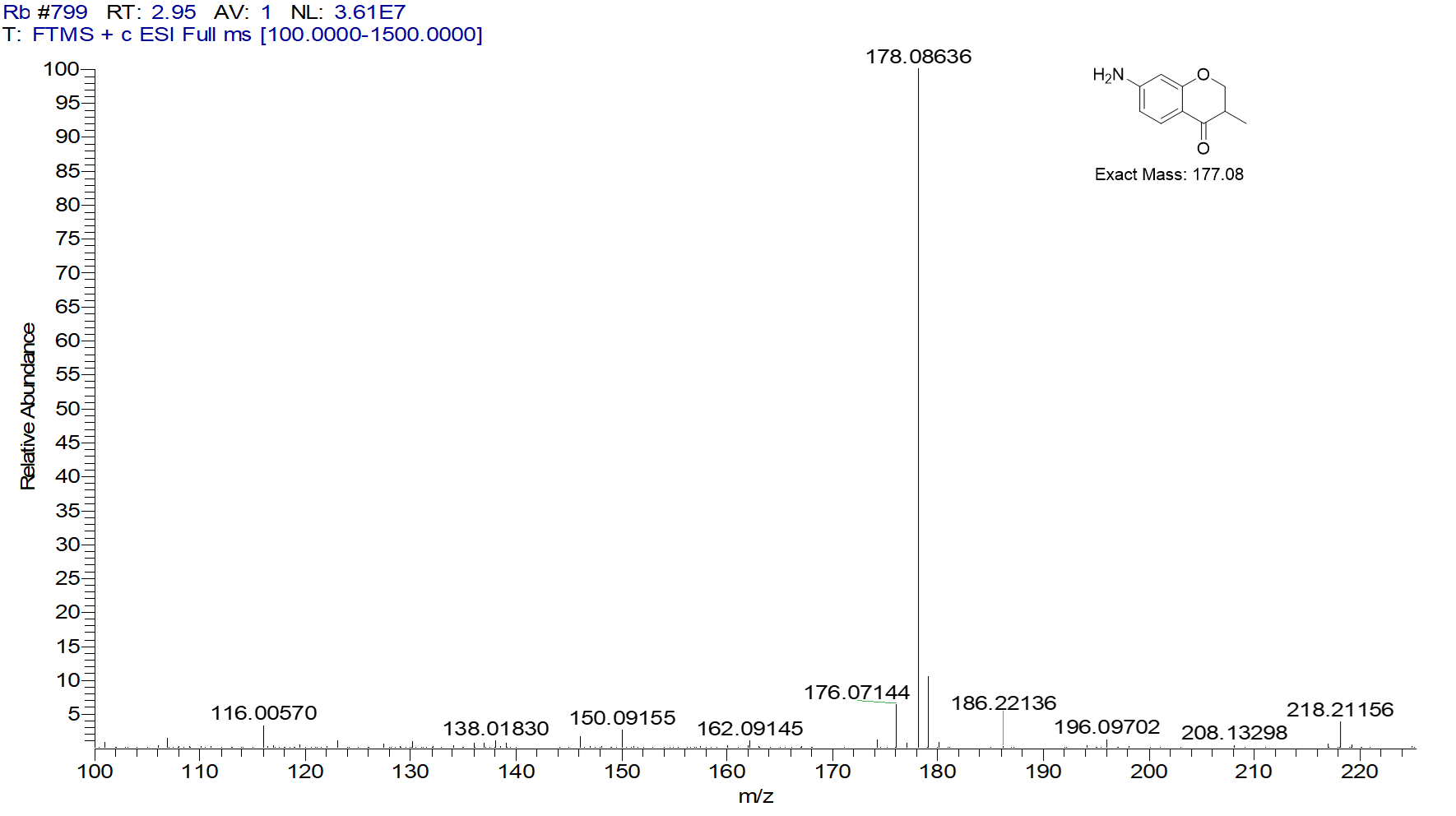


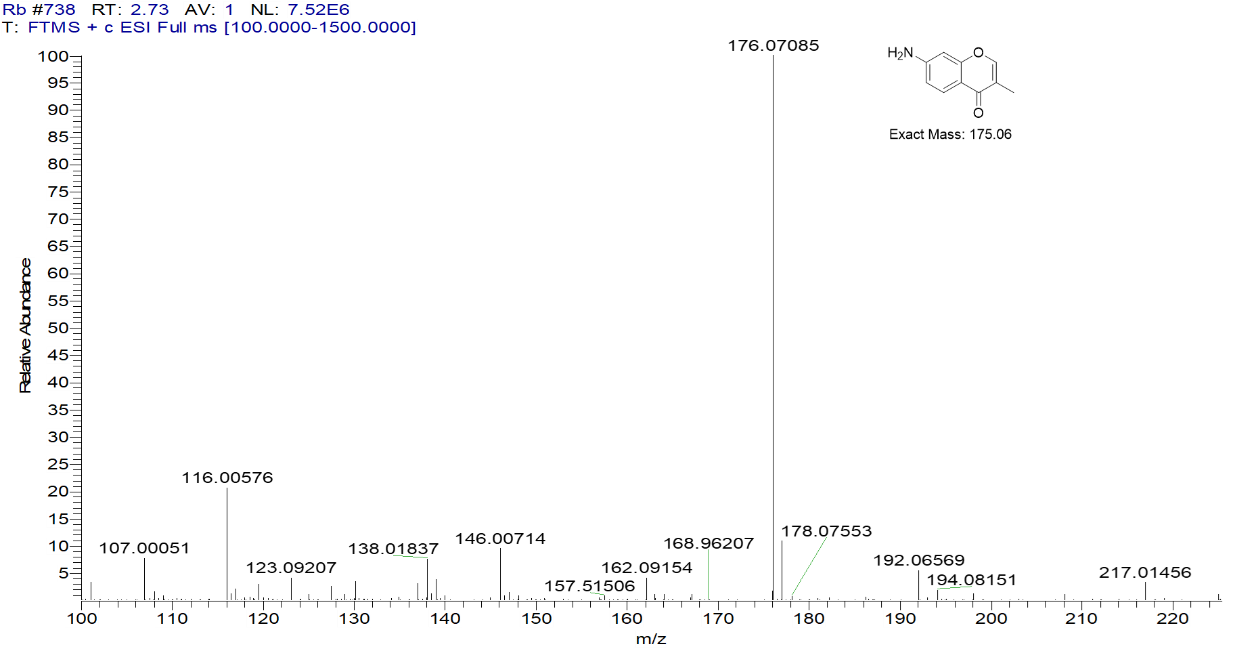


Rc probe:

We did not observe the product between Rc and superoxide anion.


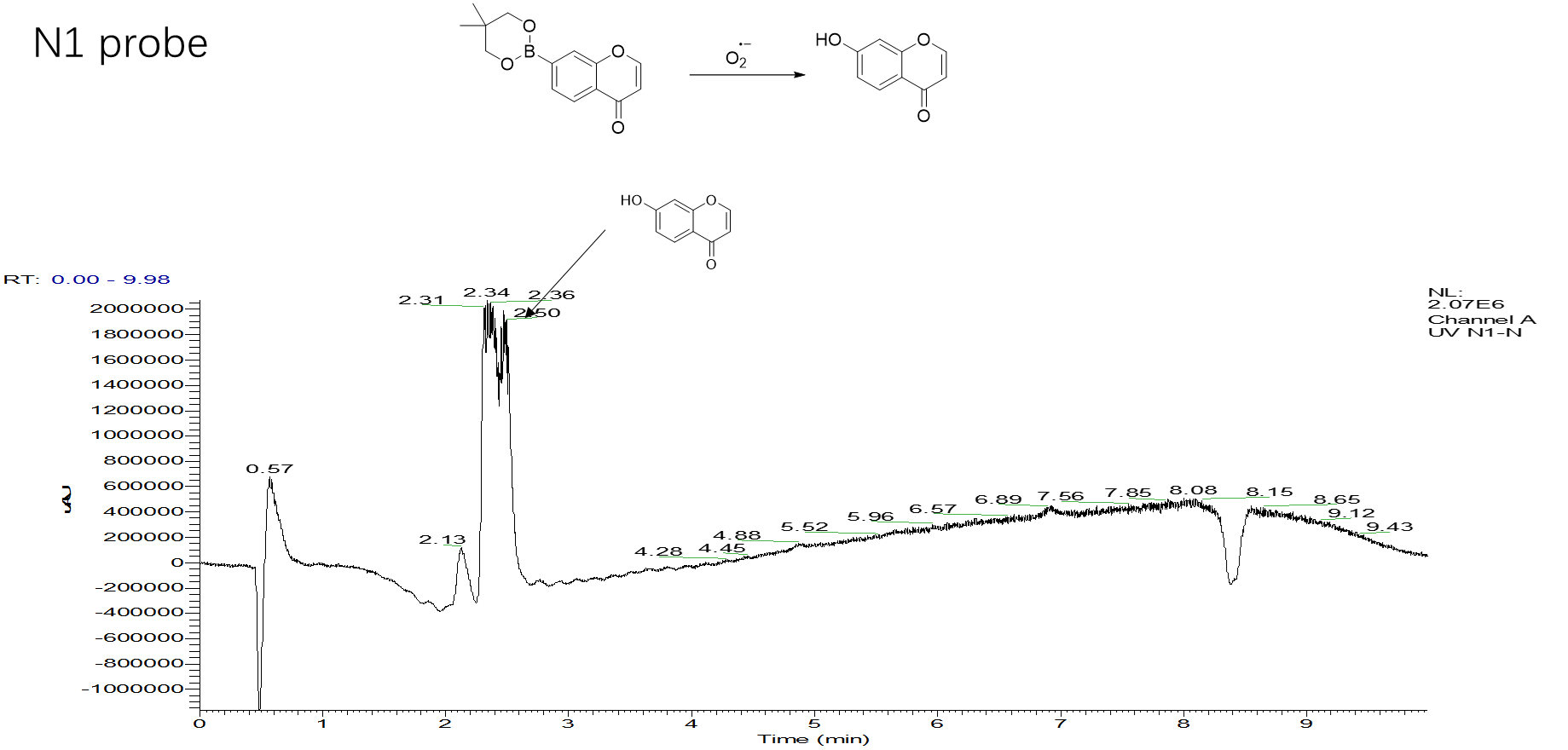


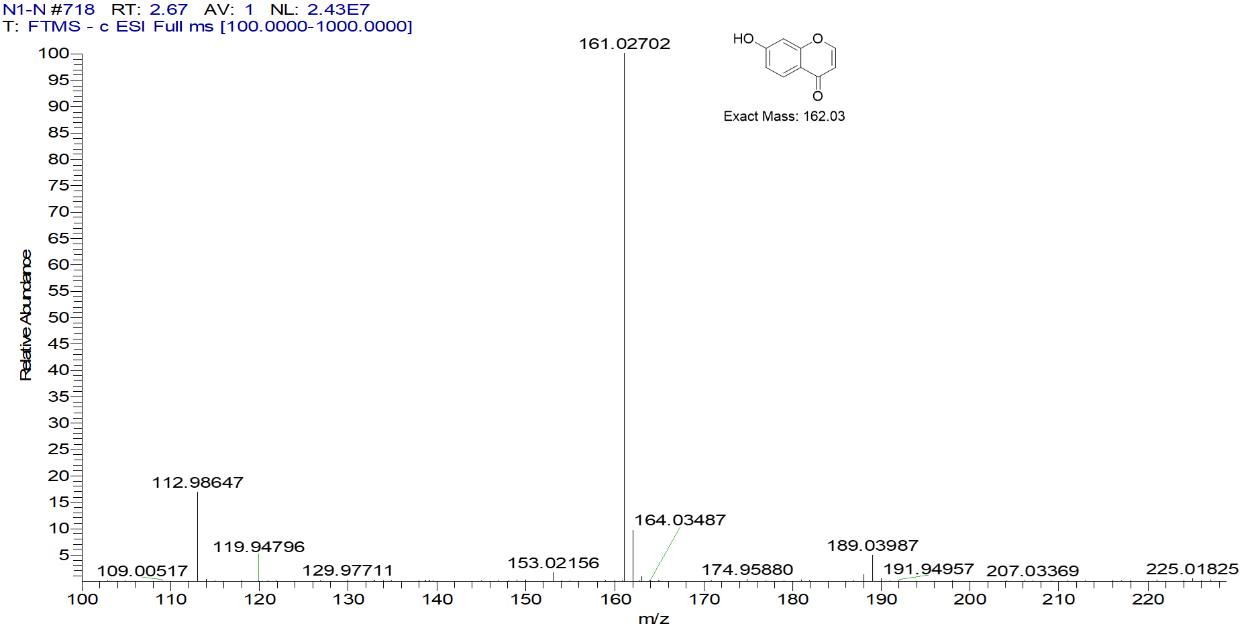


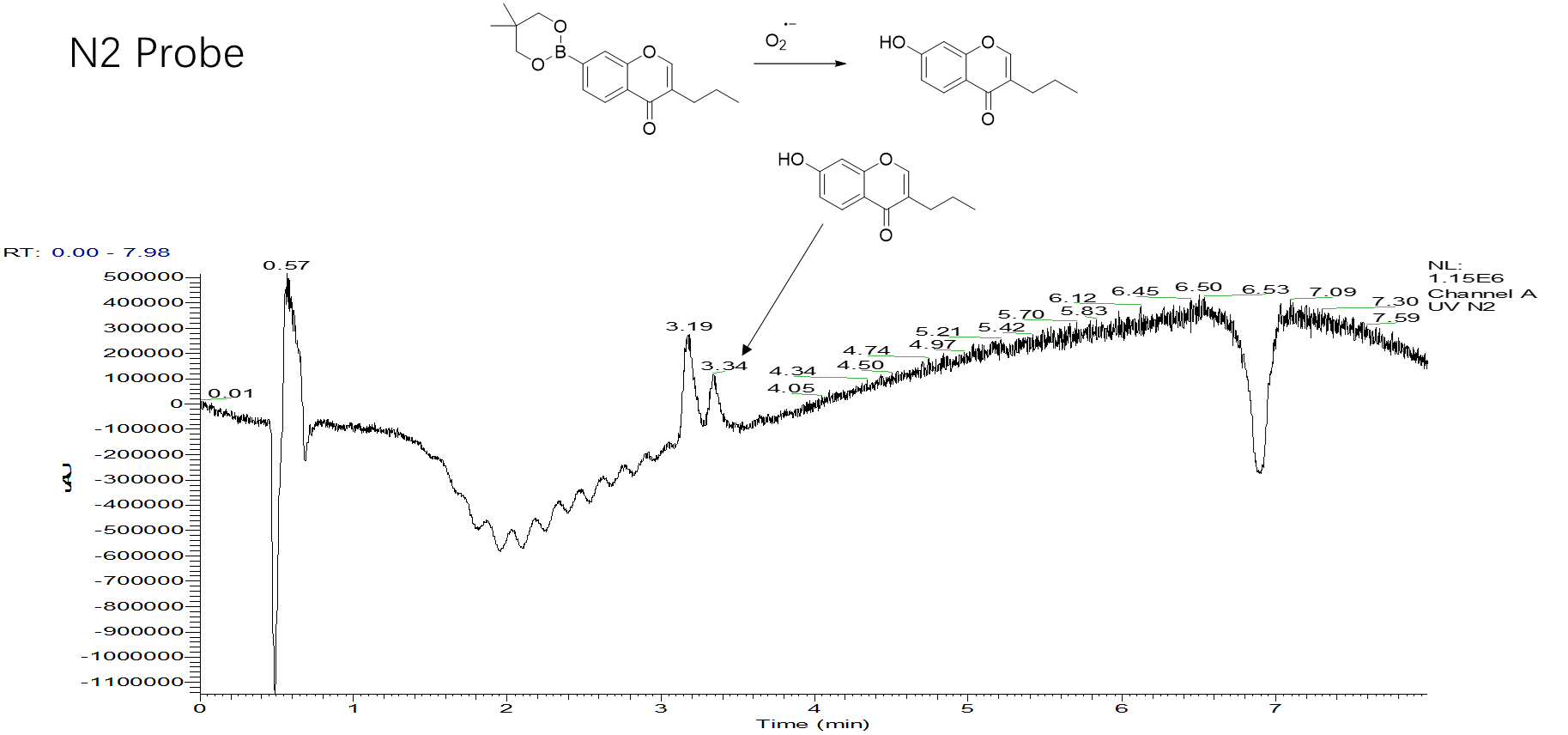


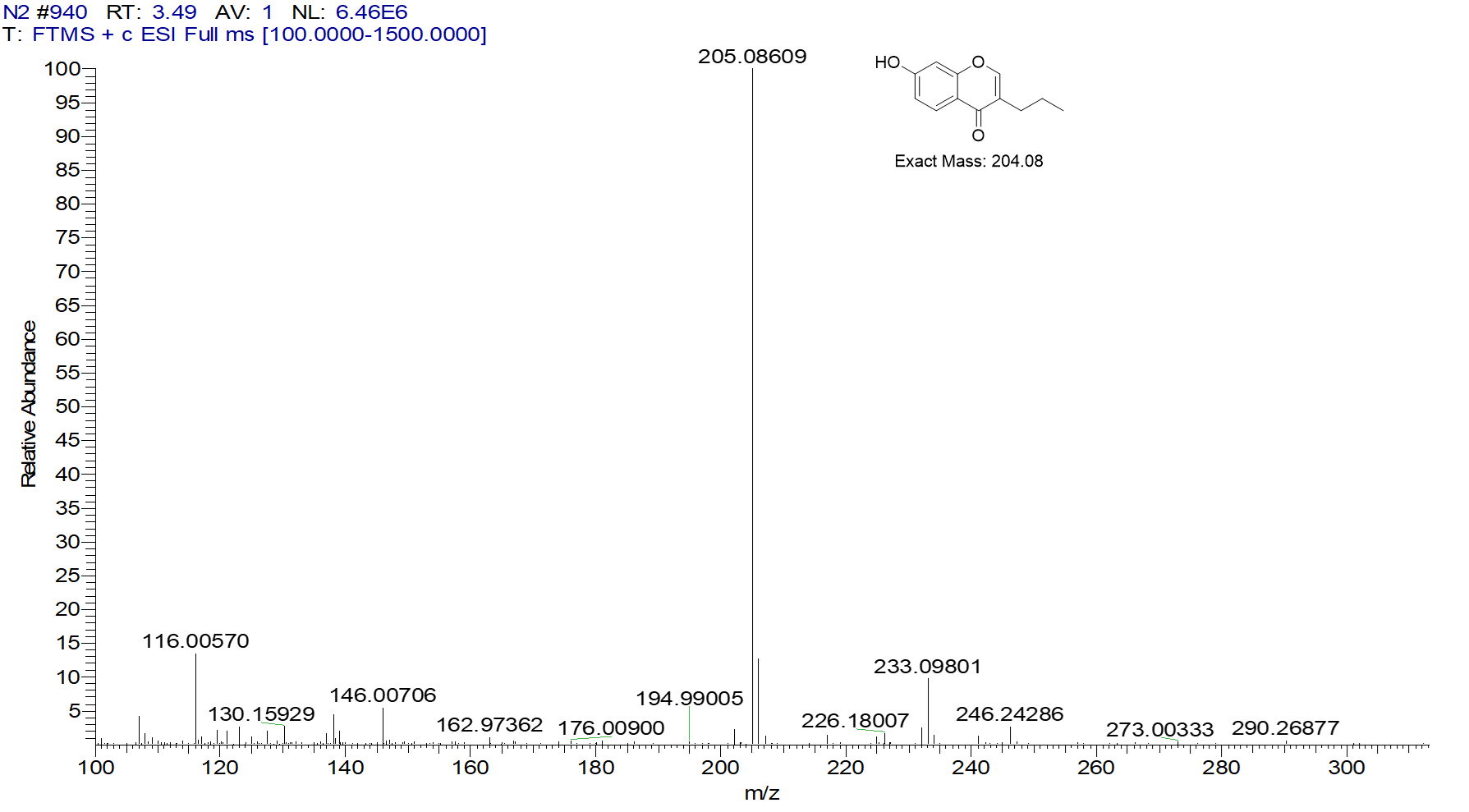


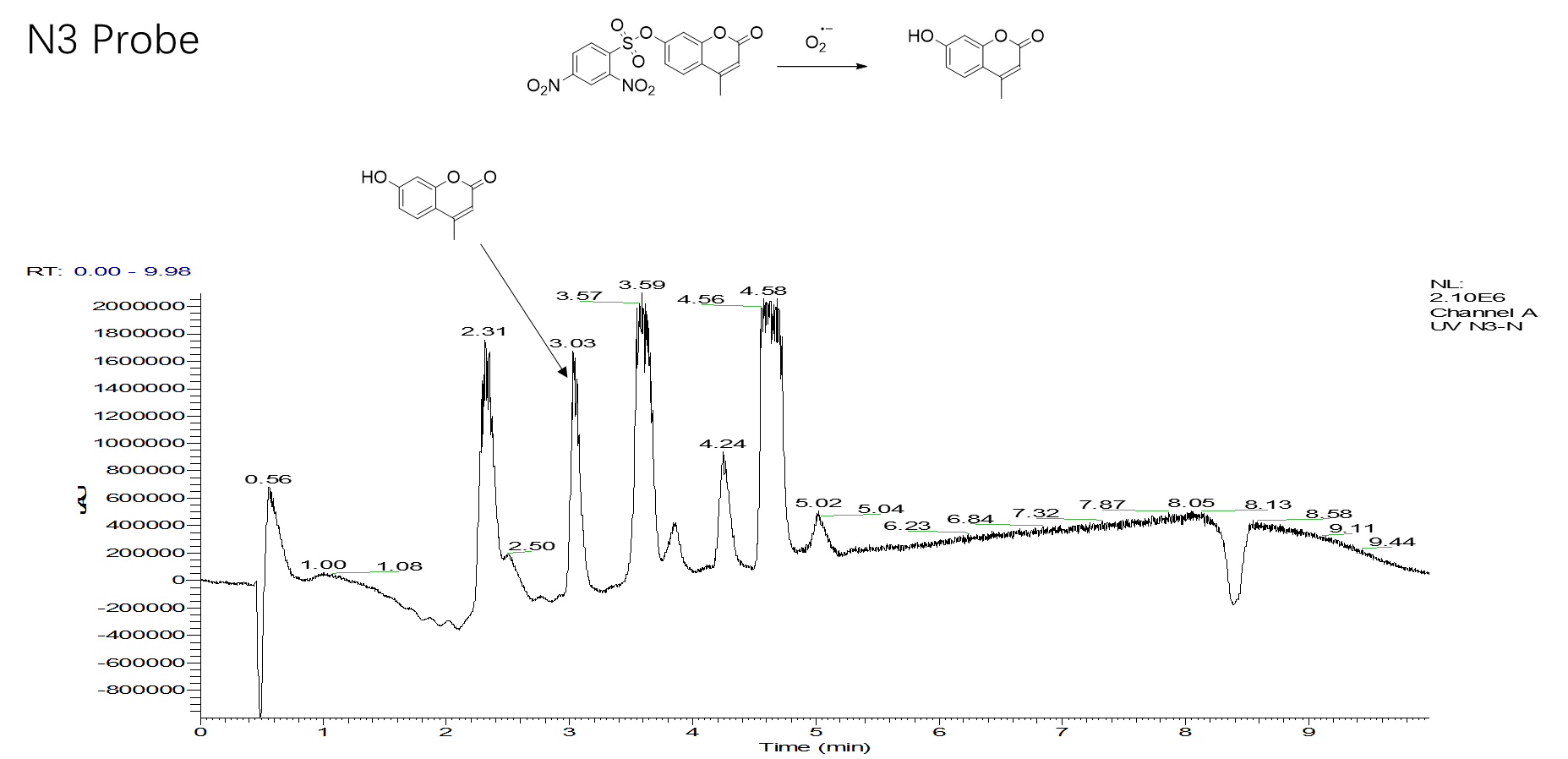


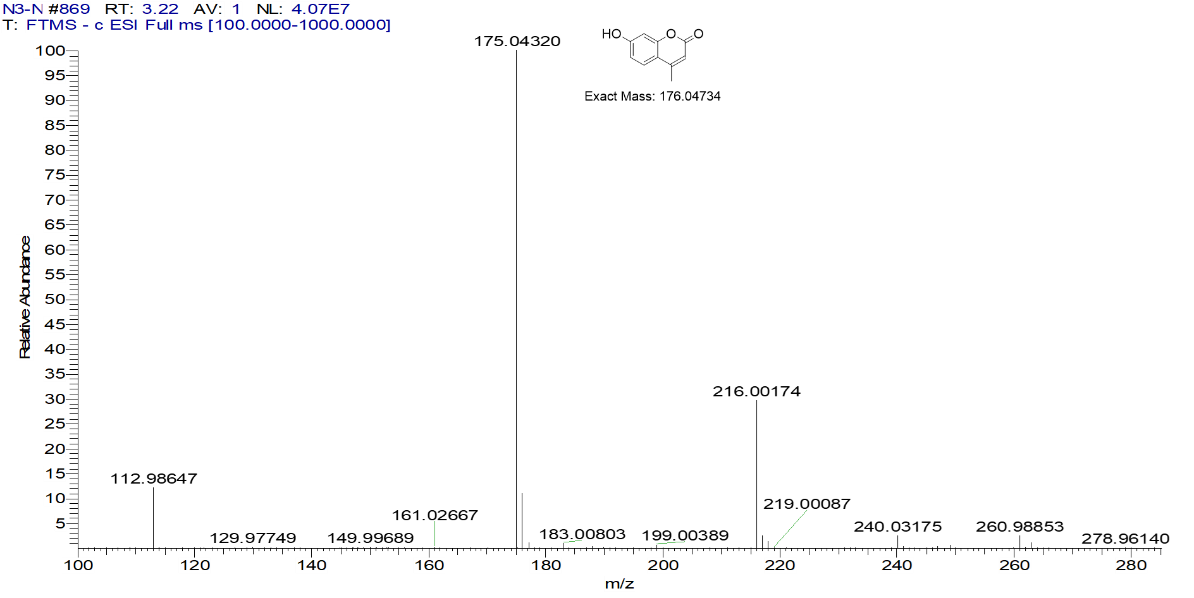


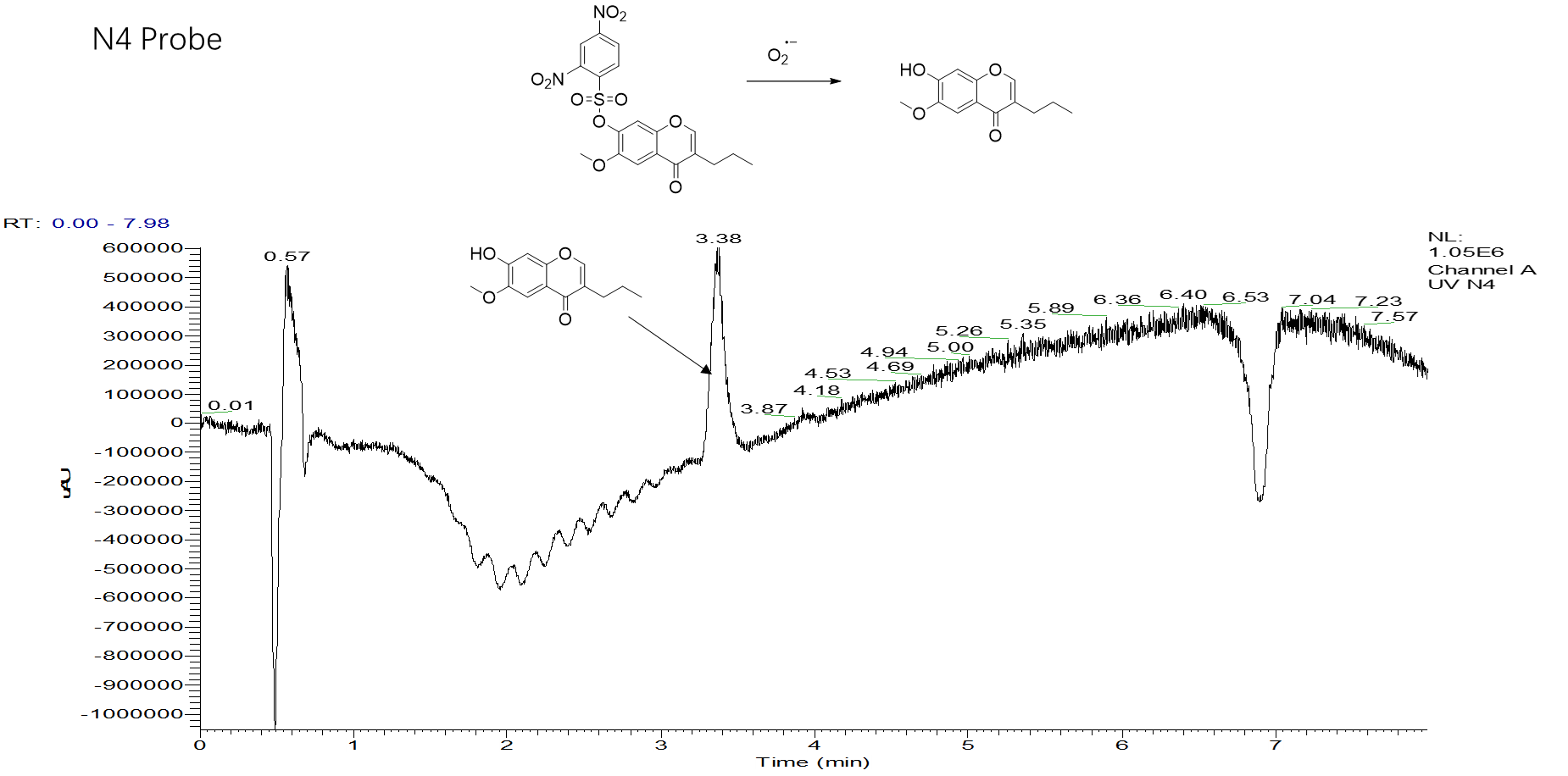


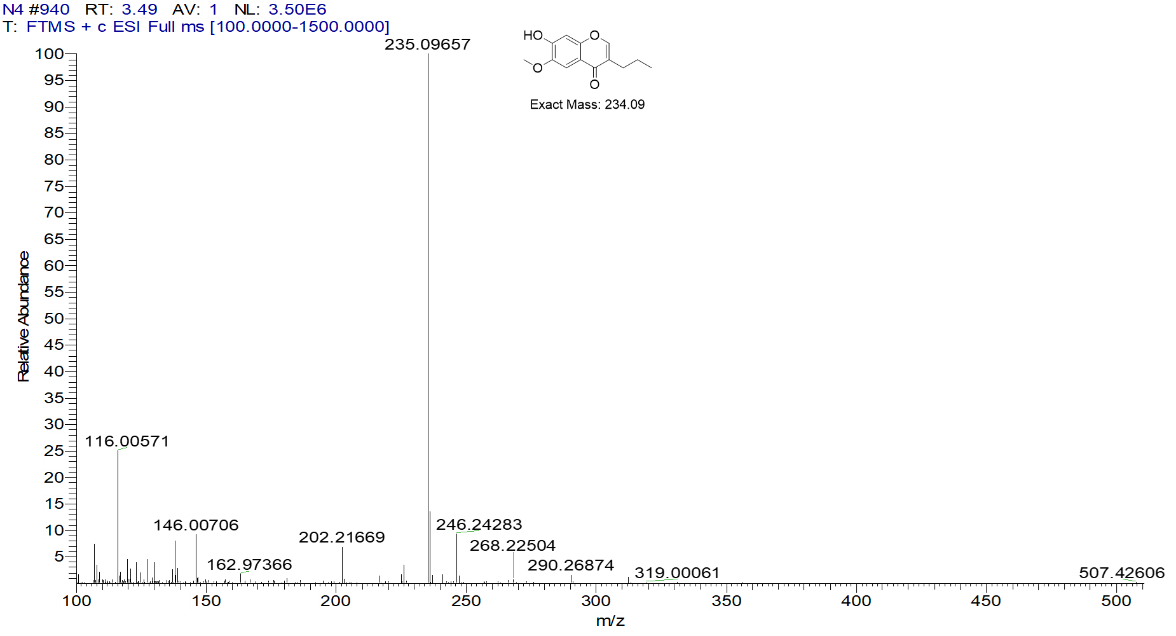


^1^H, ^13^C NMR spectra

^1^H NMR spectrum of compound **R1**


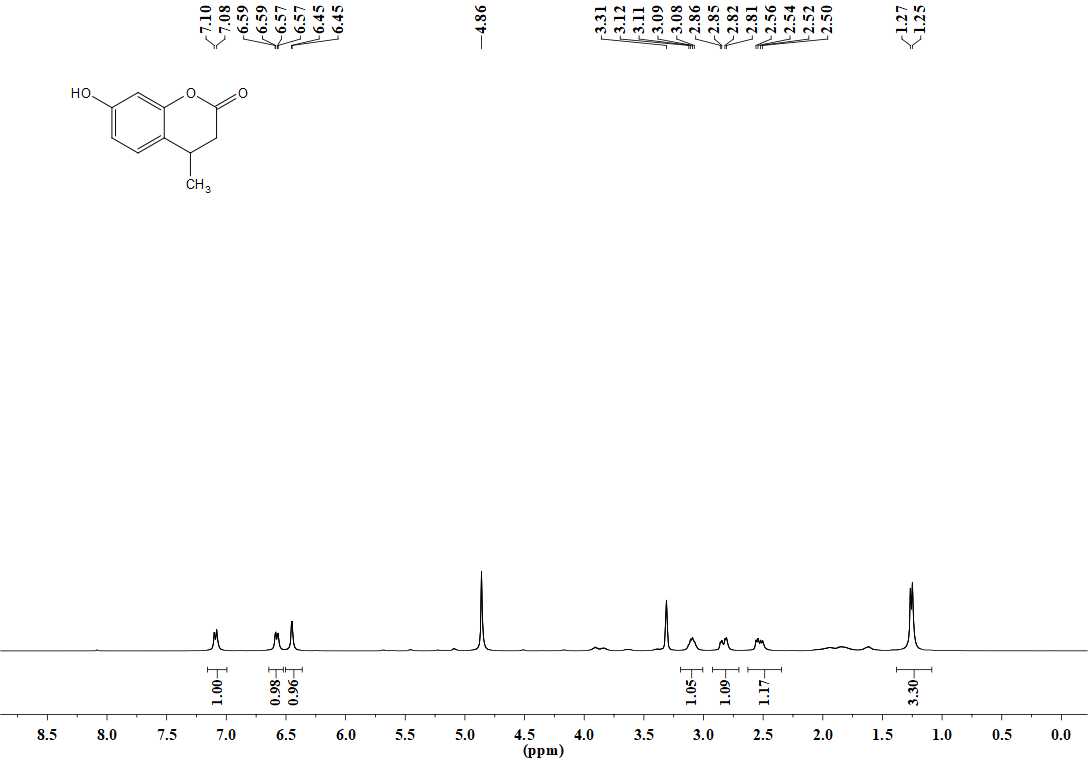


^13^C NMR spectrum of compound **R1**


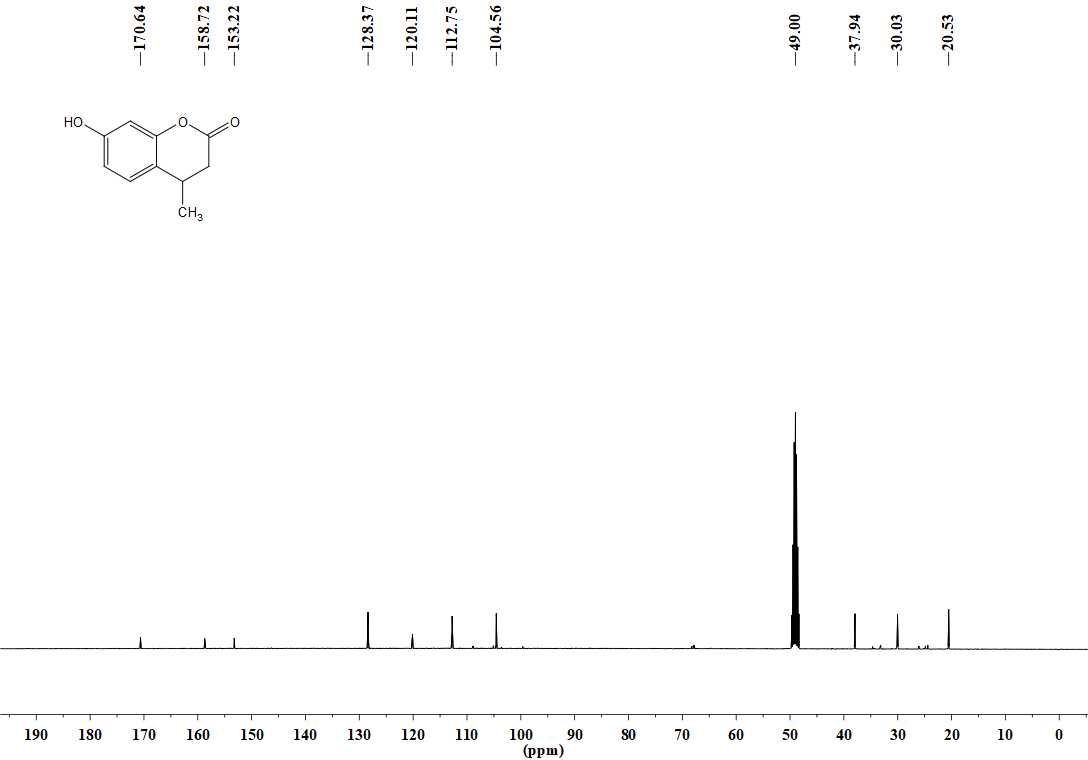


^1^H NMR spectrum of compound **R2**


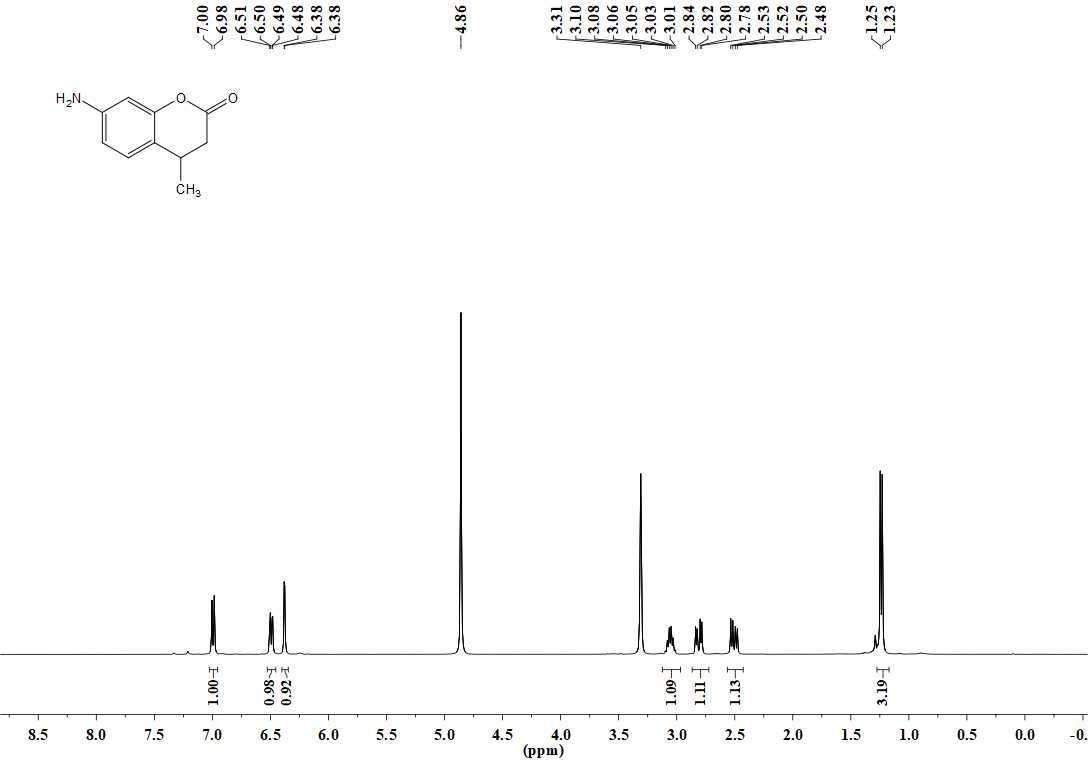


^13^C NMR spectrum of compound **R2**


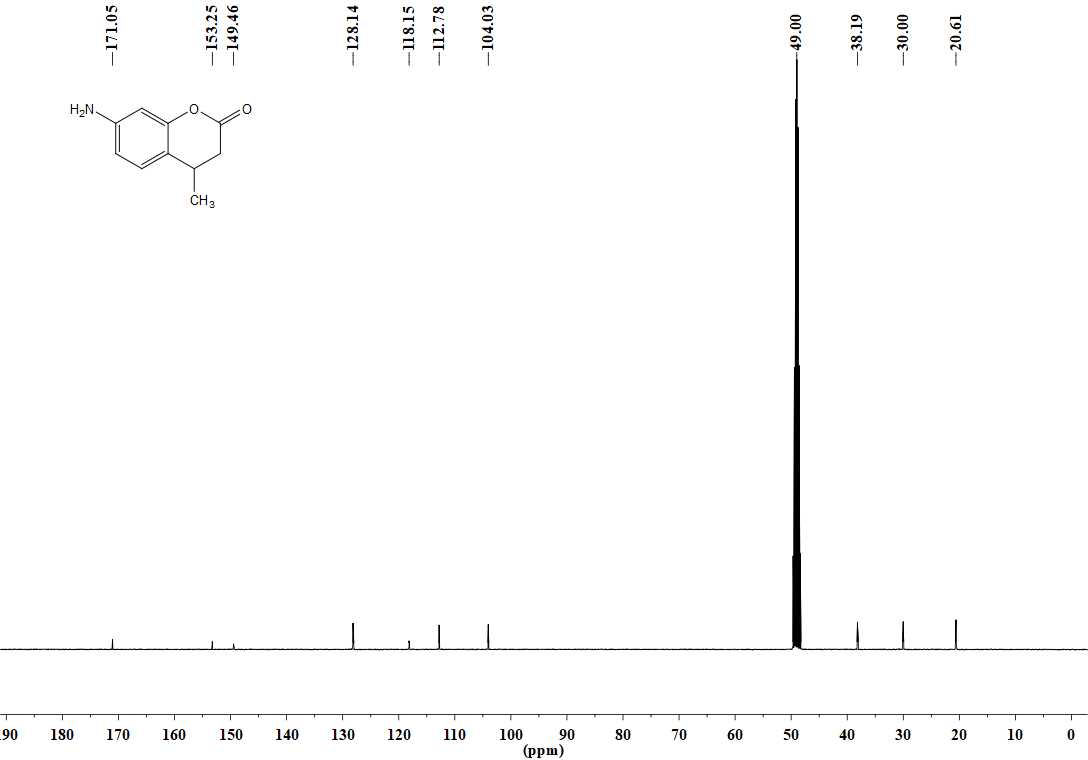


^1^H NMR spectrum of compound **R3**


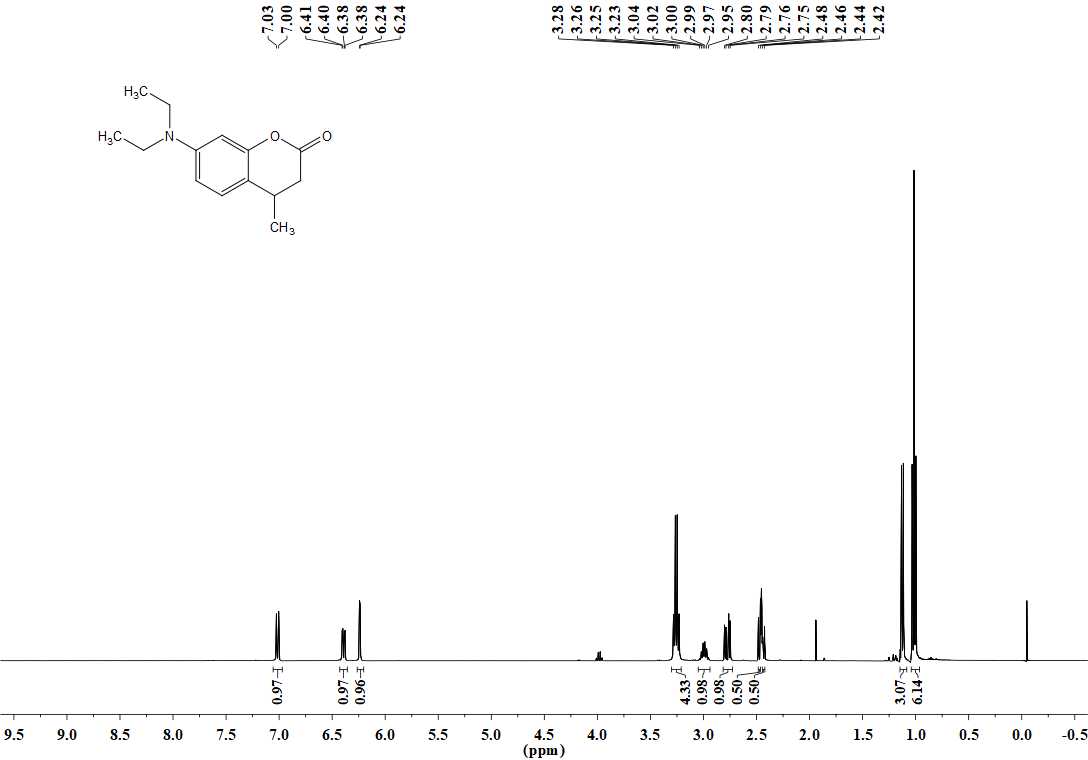


^13^C NMR spectrum of compound **R3**


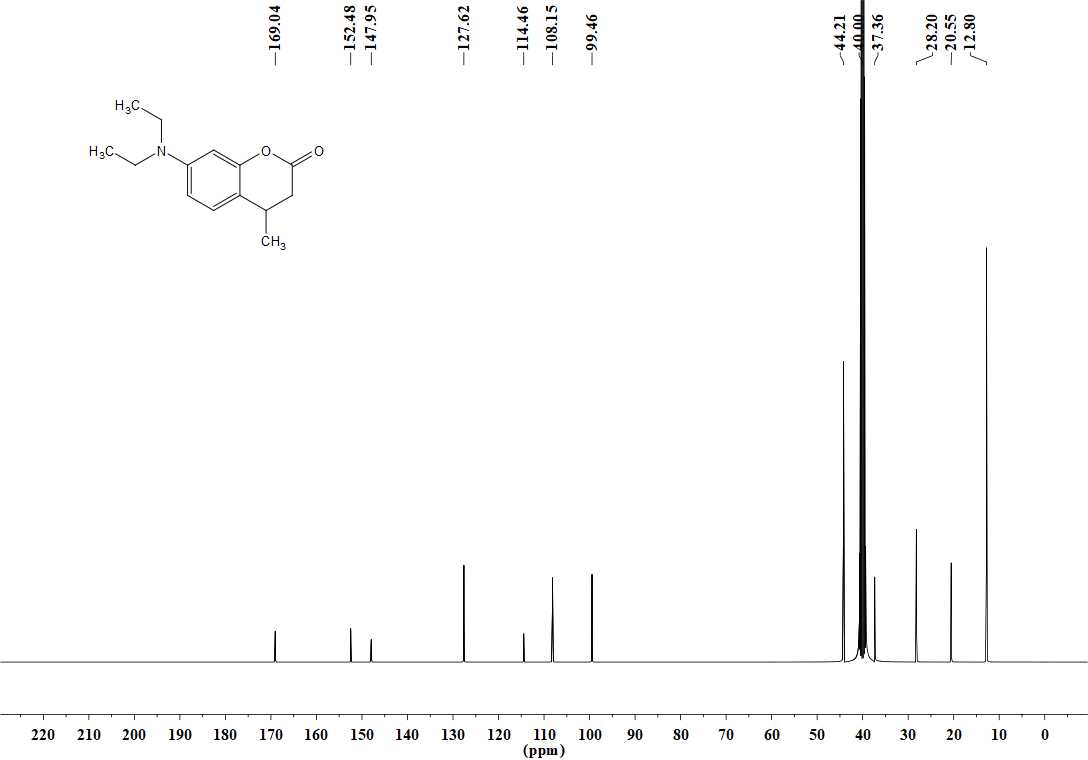


^1^H NMR spectrum of compound **Ra**


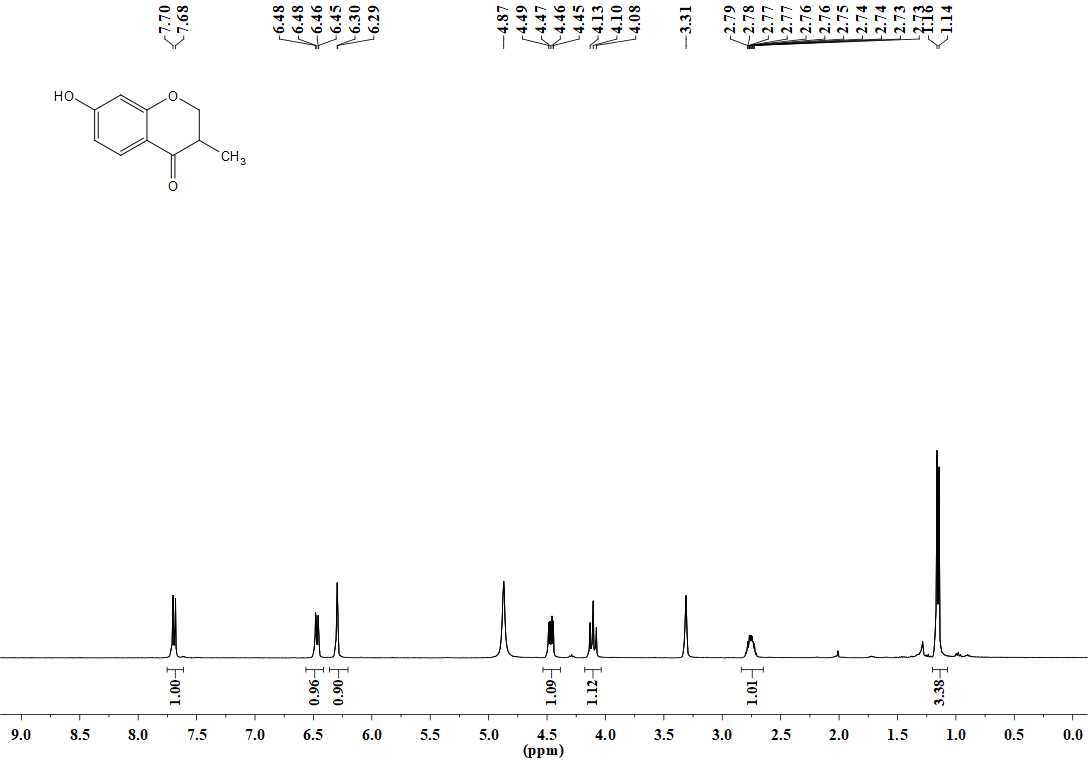


^13^C NMR spectrum of compound **Ra**


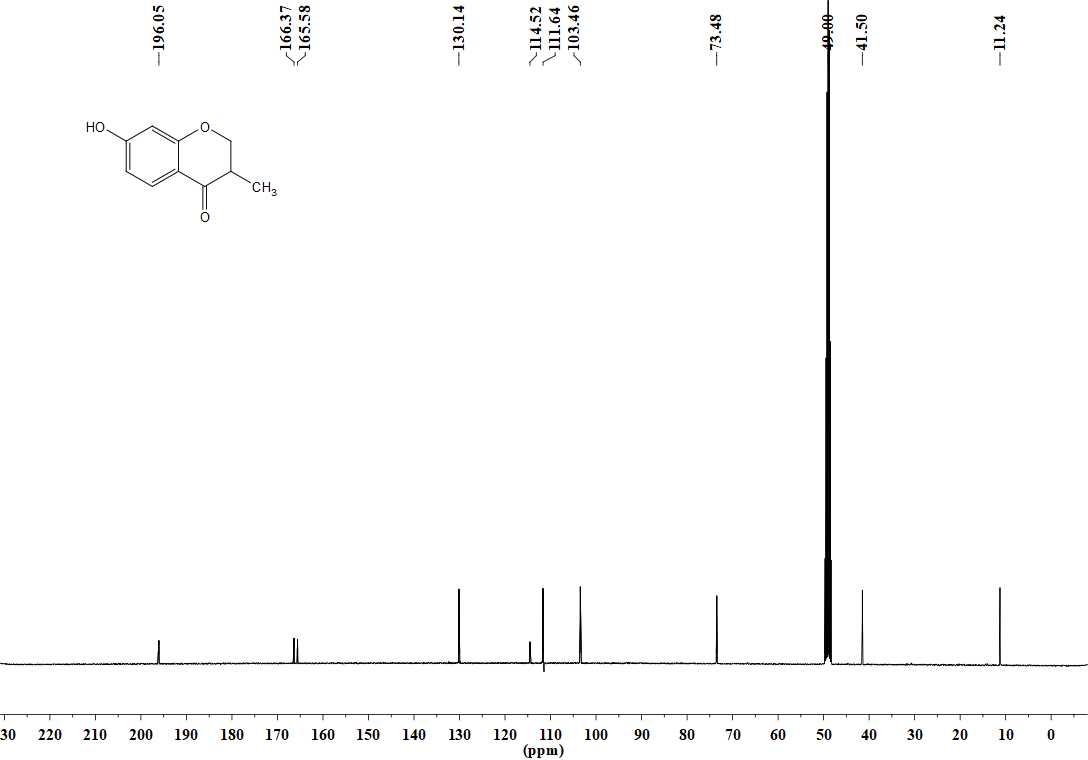


^1^H NMR spectrum of compound **Rb**


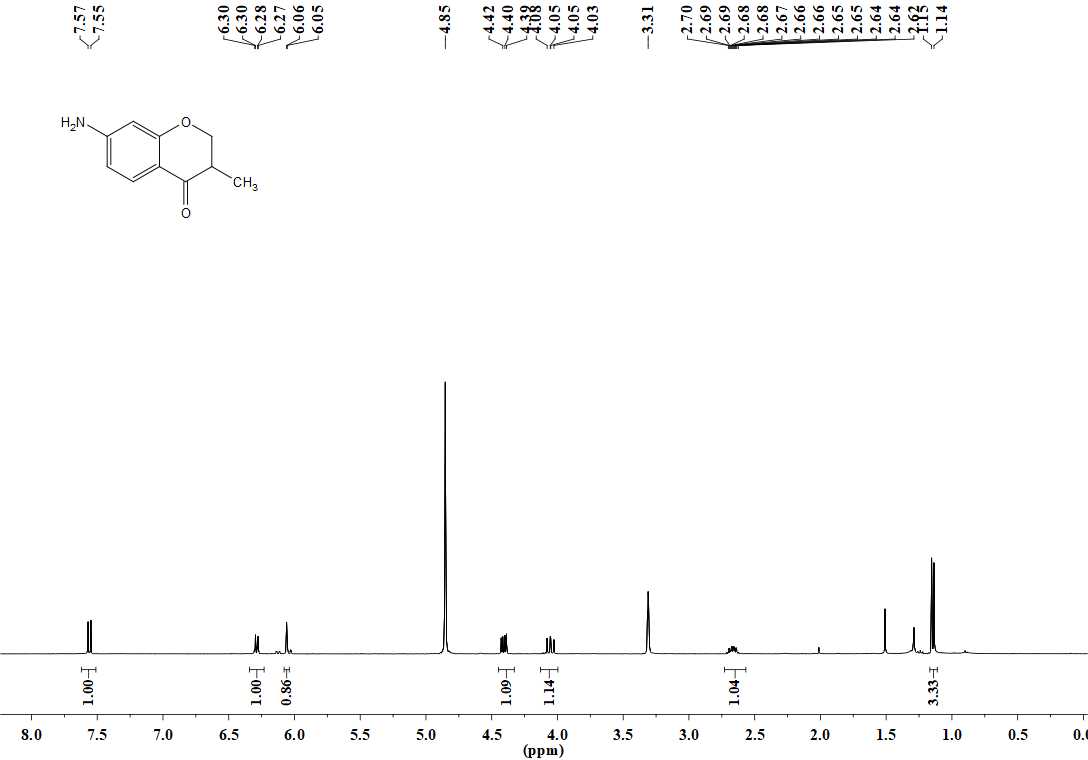


^13^C NMR spectrum of compound **Rb**


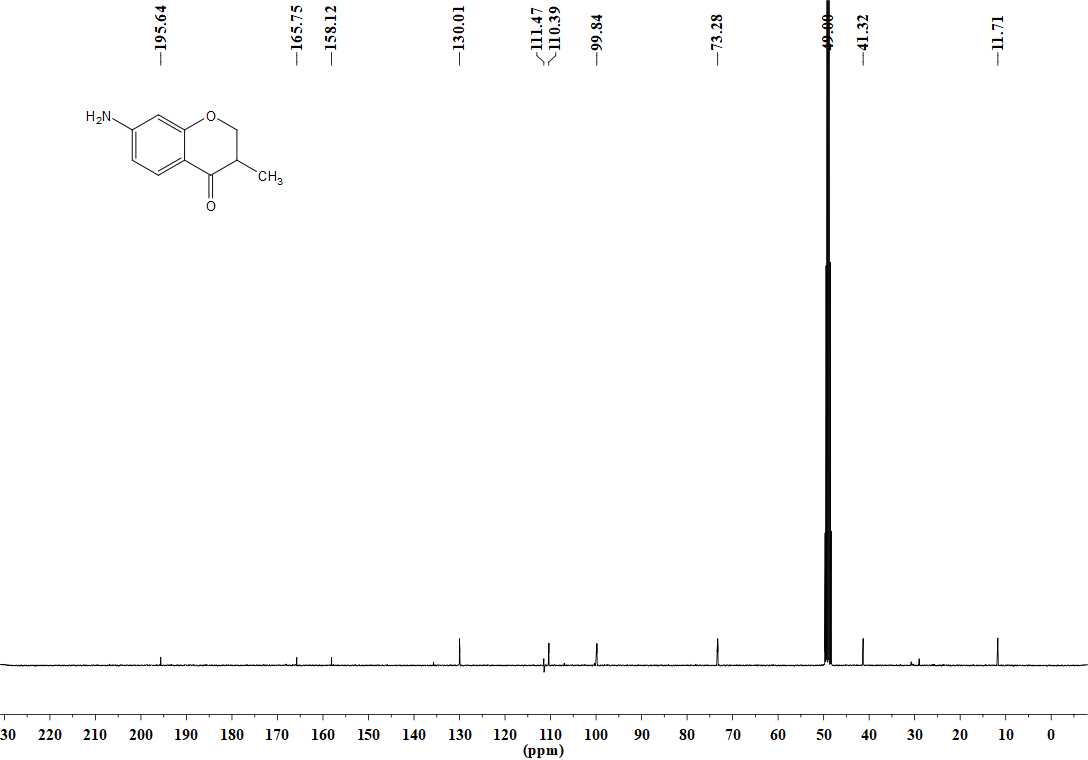


^1^H NMR spectrum of compound **Rc**


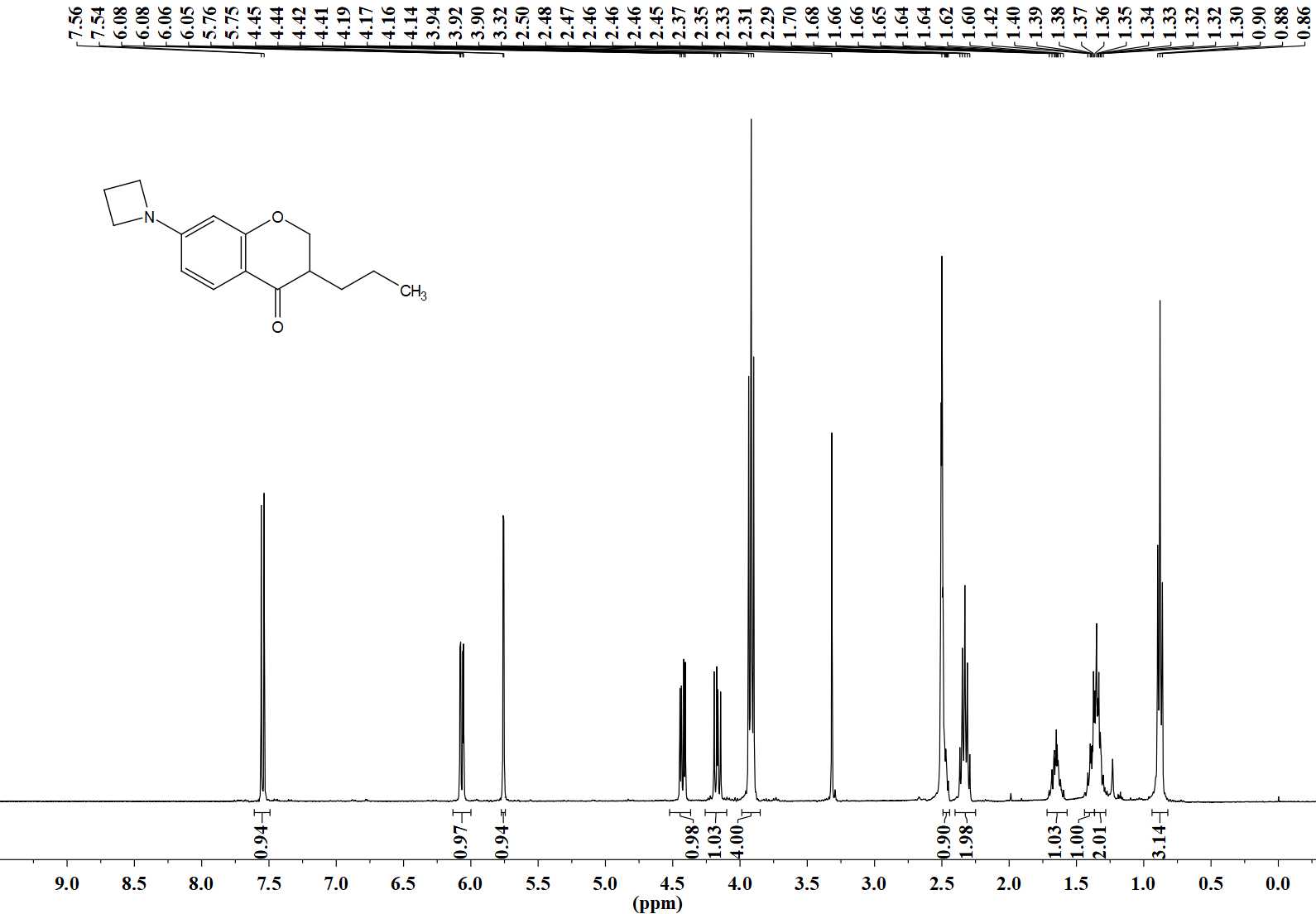


^13^C NMR spectrum of compound **Rc**


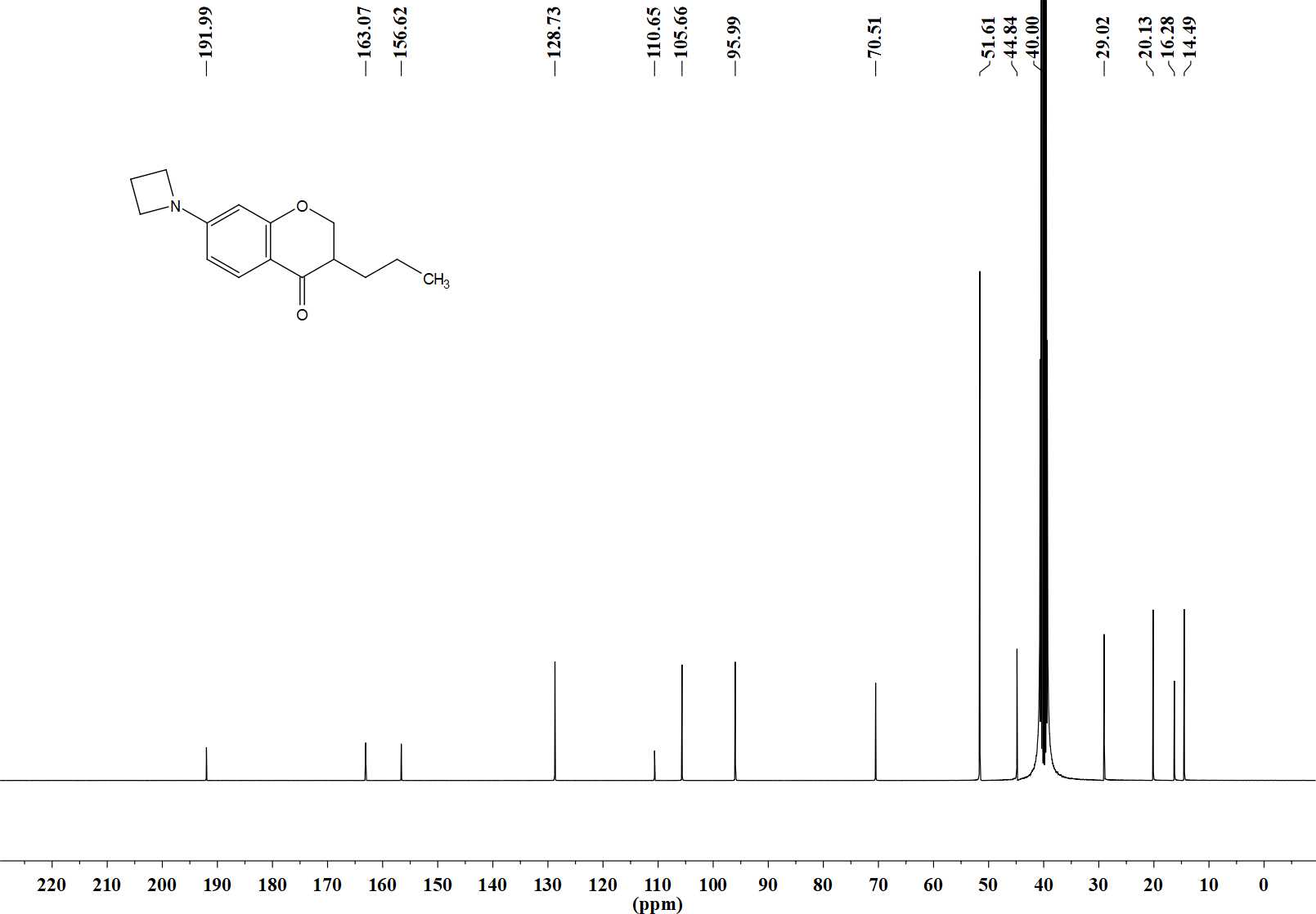


^1^H NMR spectrum of compound **N1**


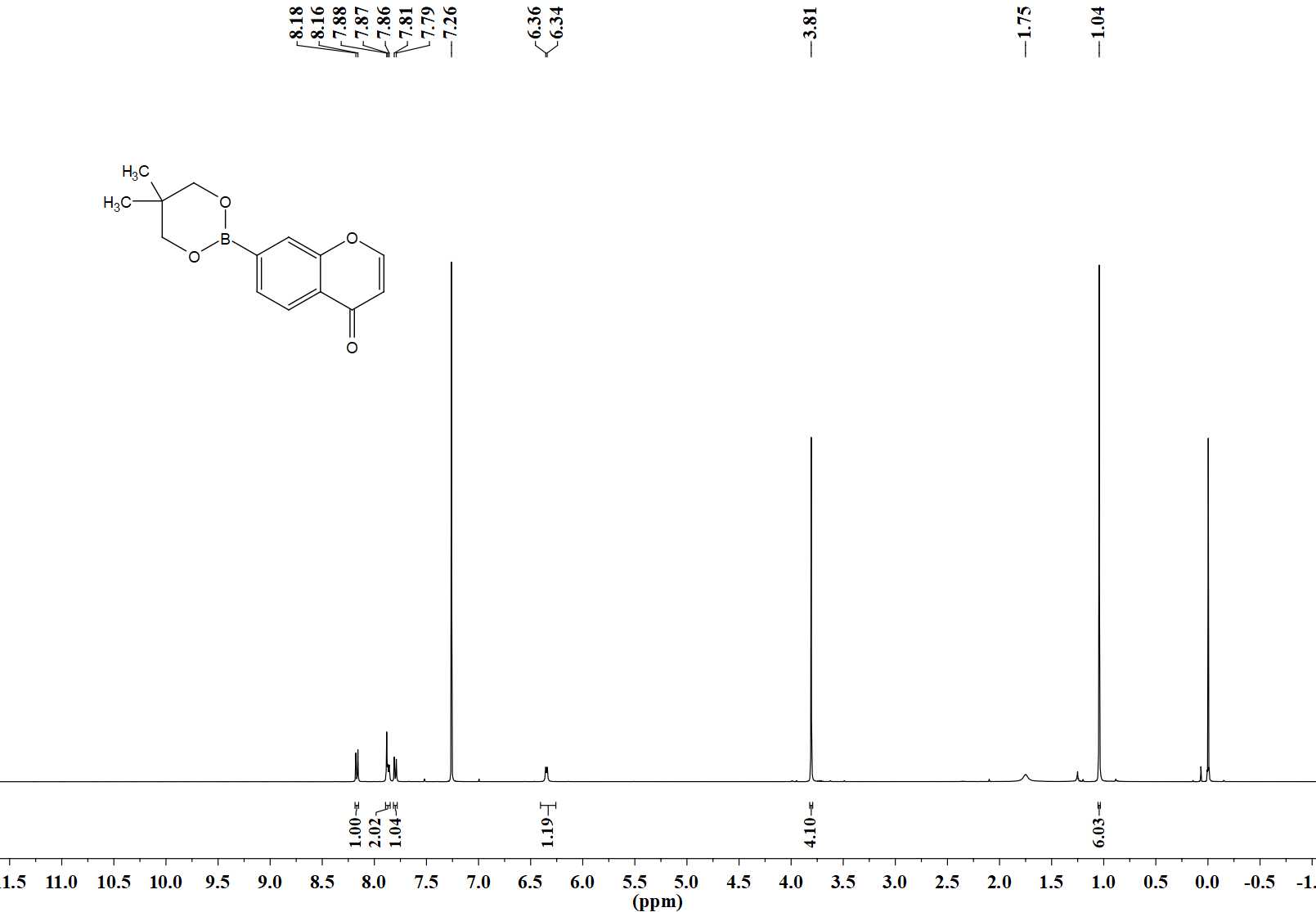


^13^C NMR spectrum of compound **N1**


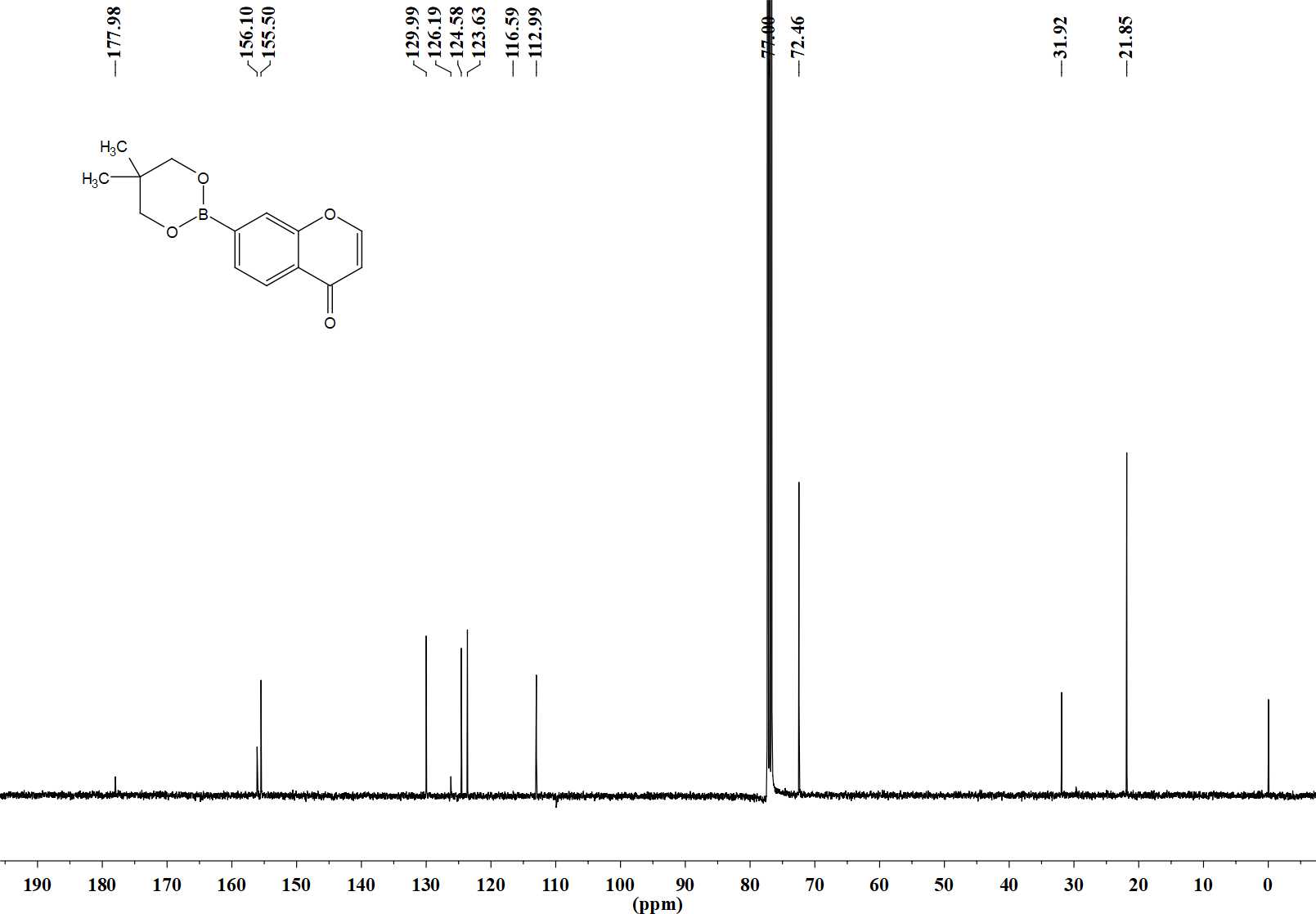


^1^H NMR spectrum of compound **N2**


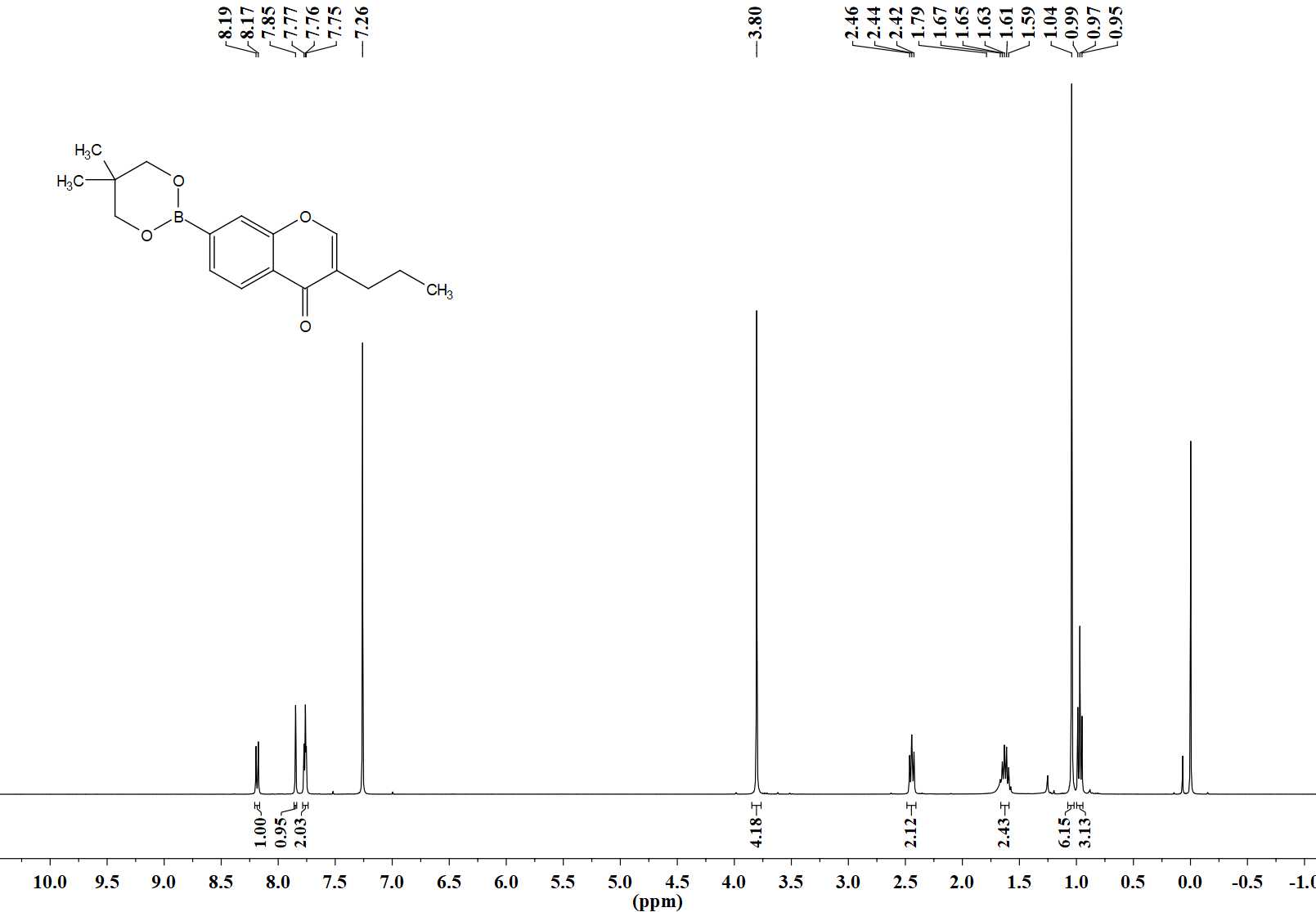


^13^C NMR spectrum of compound **N2**


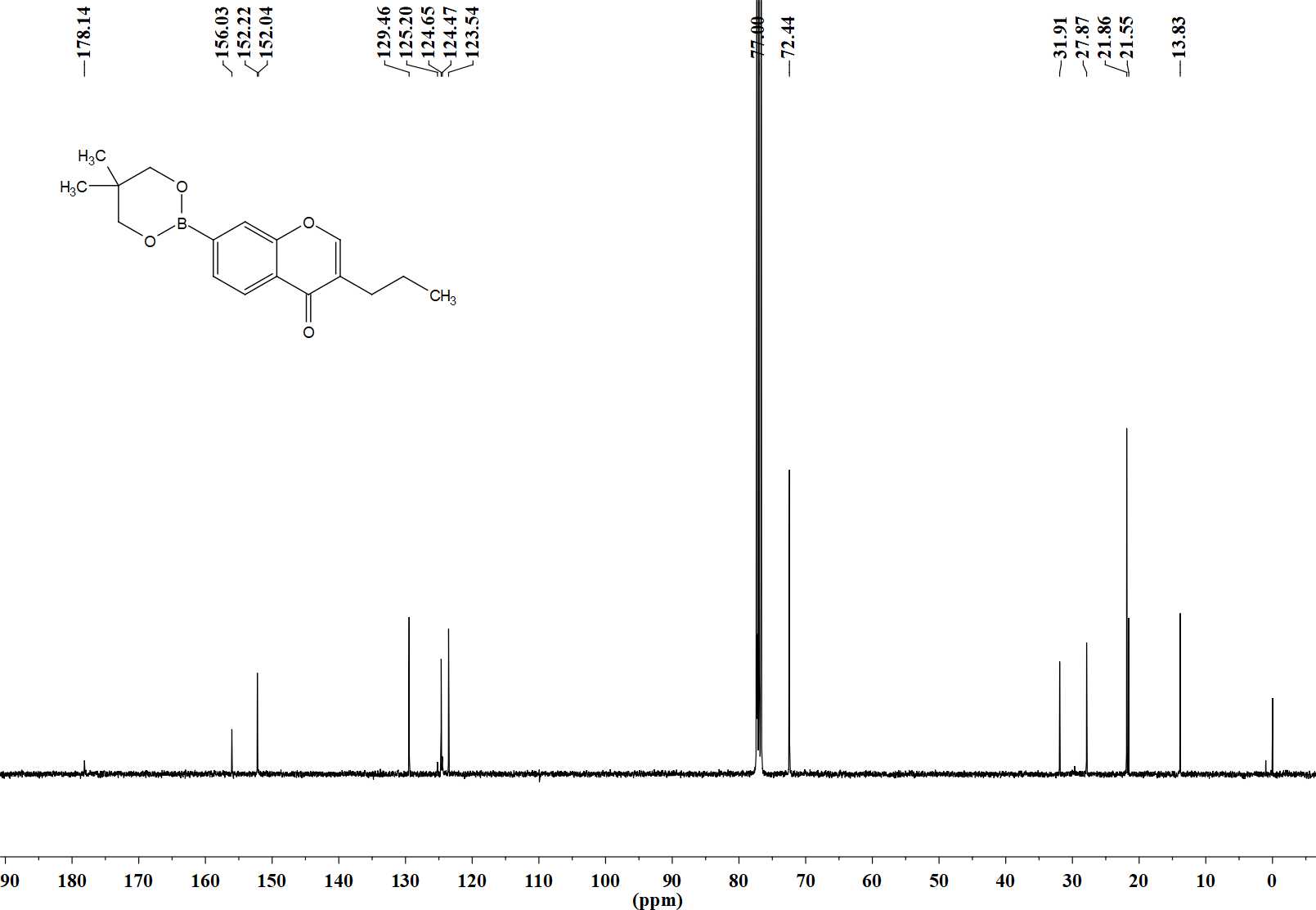


^1^H NMR spectrum of compound **N3**


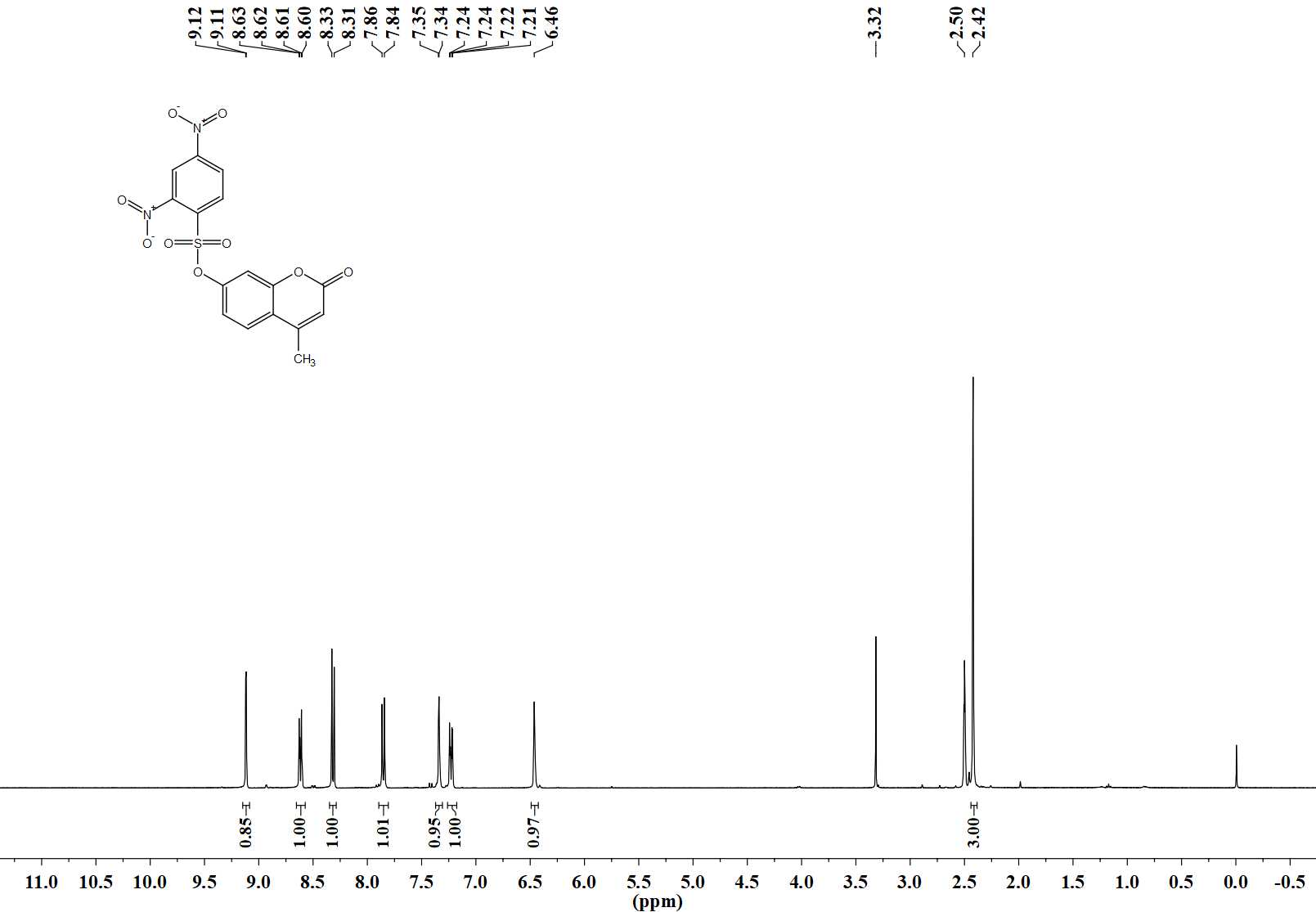


^13^C NMR spectrum of compound **N3**


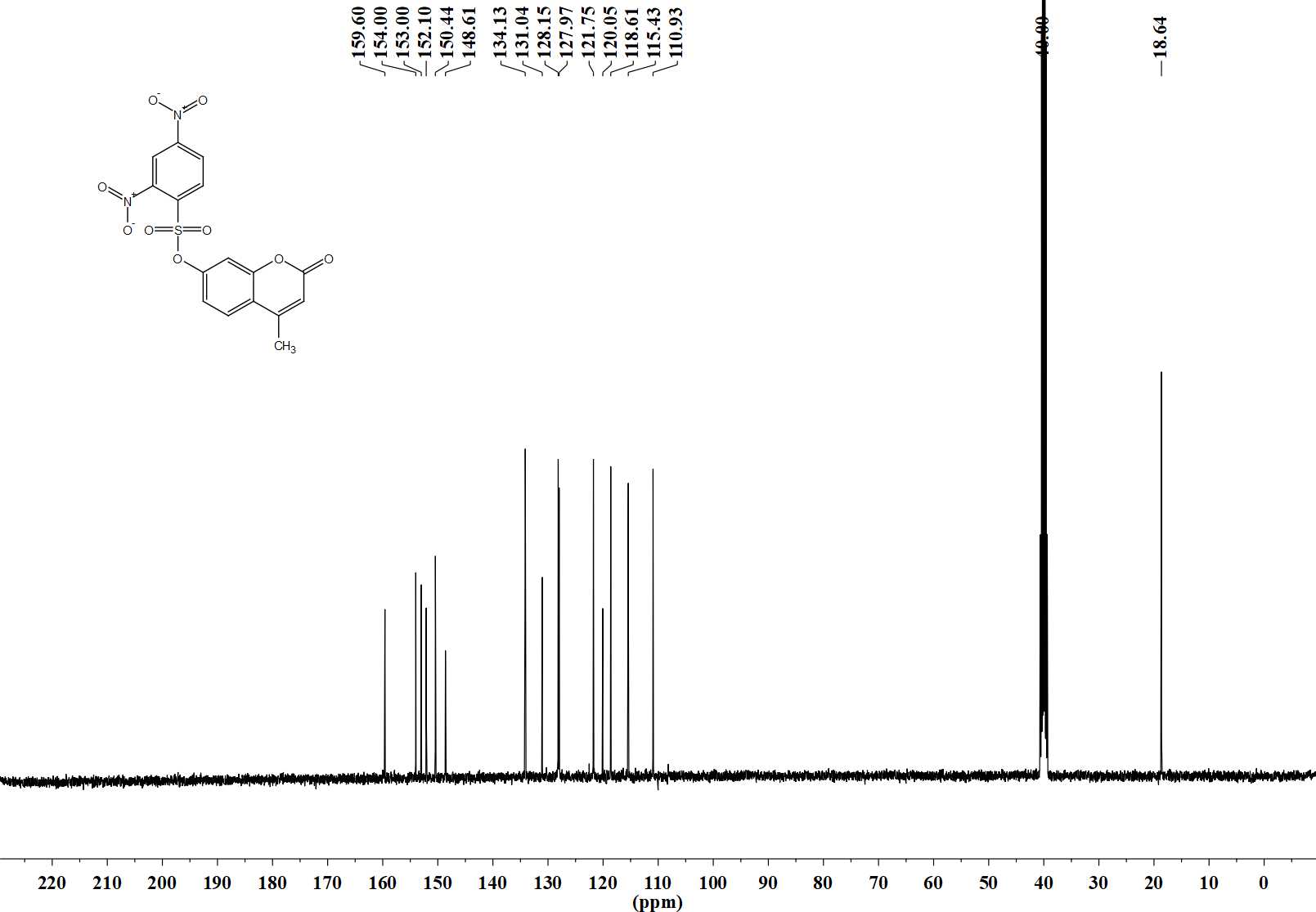


^1^H NMR spectrum of compound **N4**


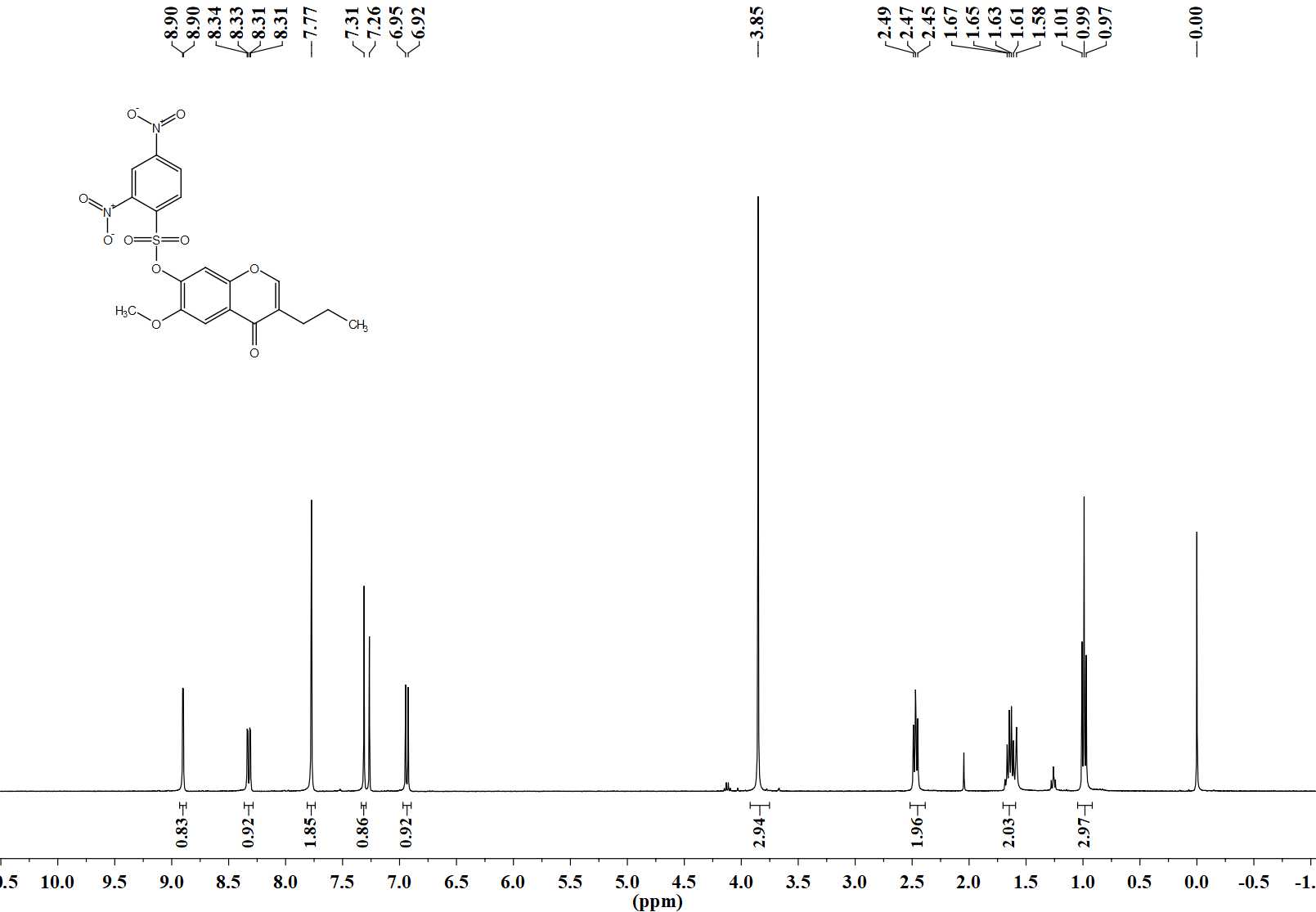


^13^C NMR spectrum of compound **N4**


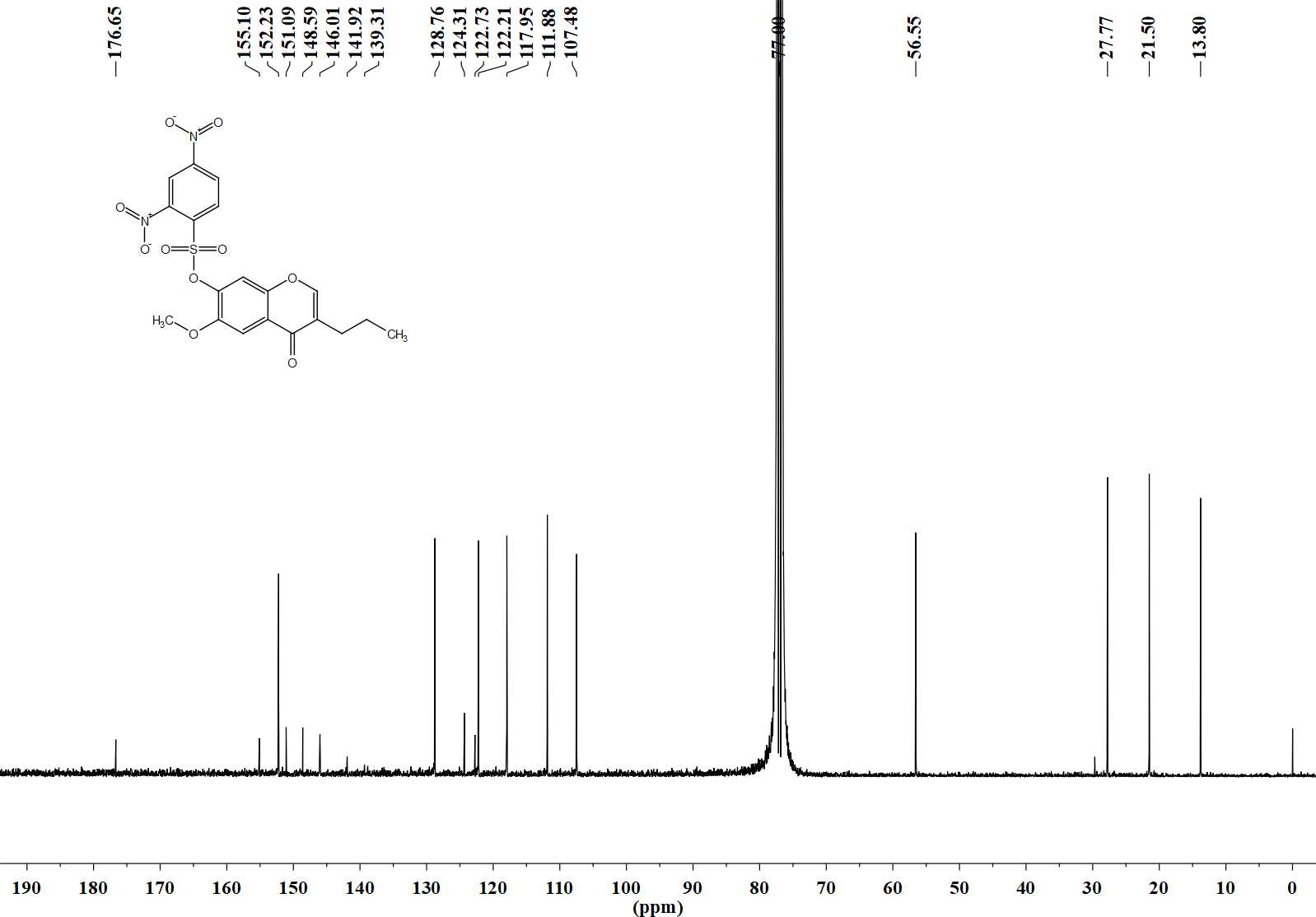


1. Chen Y, Gao Y, He Y, Zhang G, Wen H, Wang Y, Wu QP, Cui H: **Determining Essential Requirements for Fluorophore Selection in Various Fluorescence Applications Taking Advantage of Diverse Structure-Fluorescence Information of Chromone Derivatives**. *J Med Chem* 2021, **64**(2):1001-1017.

2. Wang Y, Han J, Xu Y, Gao Y, Wen H, Cui H: **Taking advantage of the aromatisation of 7-diethylamino-4-methyl-3,4-dihydrocoumarin in the fluorescence sensing of superoxide anion**. *Chem Commun (Camb)* 2020, **56**(68):9827-9829.
