# Supplementary figures and images for: Insights Into the Detection Selectivity of Redox and Non-redox Based Probes for the Superoxide Anion Using Coumarin and Chromone as the Fluorophores
Source: Front Chem. 2021 Nov 25;9:753621. doi: 10.3389/fchem.2021.753621 (PMC8667960; doi:10.3389/fchem.2021.753621)

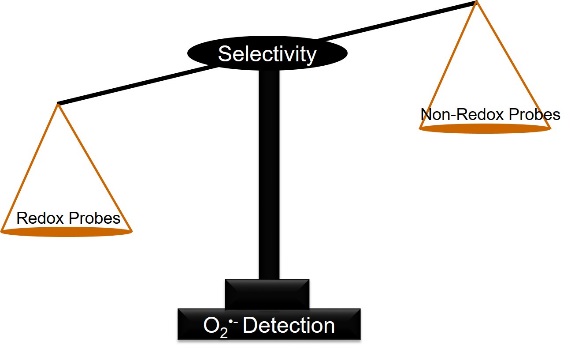

Supplement: Supplementary file 2 [file Table2.DOCX]
